# *Cryptosporidium* infections in terrestrial ungulates with focus on livestock: a systematic review and meta-analysis

**DOI:** 10.1186/s13071-019-3704-4

**Published:** 2019-09-14

**Authors:** Kareem Hatam-Nahavandi, Ehsan Ahmadpour, David Carmena, Adel Spotin, Berit Bangoura, Lihua Xiao

**Affiliations:** 1 Iranshahr University of Medical Sciences, Iranshahr, Iran; 2 Infectious and Tropical Diseases Research Center, Tabriz University of Medical Sciences, Tabriz, Iran; 3 Parasitology Reference and Research Laboratory, National Centre for Microbiology, Carlos III Health Institute, Ctra Majadahonda-Pozuelo Km 2, 28220 Majadahonda, Madrid, Spain; 4 Immunology Research Center, Tabriz University of Medical Sciences, Tabriz, Iran; 5 Drug Applied Research Center, Tabriz University of Medical Sciences, Tabriz, Iran; 6 Department of Veterinary Sciences, College of Agriculture and Natural Resources, University of Wyoming, Laramie, WY USA; 7 College of Veterinary Medicine, South China Agricultural University, Guangzhou, China

**Keywords:** Cryptosporidiosis, Livestock, Cattle, Sheep, Goat, Pig, Horse, Wildlife

## Abstract

**Background:**

*Cryptosporidium* spp. are causative agents of gastrointestinal diseases in a wide variety of vertebrate hosts. Mortality resulting from the disease is low in livestock, although severe cryptosporidiosis has been associated with fatality in young animals.

**Methods:**

The goal of this systematic review and meta-analysis was to review the prevalence and molecular data on *Cryptosporidium* infections in selected terrestrial domestic and wild ungulates of the families Bovidae (bison, buffalo, cattle, goat, impala, mouflon sheep, sheep, yak), Cervidae (red deer, roe deer, white-tailed deer), Camelidae (alpaca, camel), Suidae (boar, pig), Giraffidae (giraffes) and Equidae (horses). Data collection was carried out using PubMed, Scopus, Science Direct and Cochran databases, with 429 papers being included in this systematic analysis.

**Results:**

The results show that overall 18.9% of ungulates from the investigated species were infected with *Cryptosporidium* spp. Considering livestock species (cattle, sheep, goats, pigs, horses and buffaloes), analysis revealed higher *Cryptosporidium* infection prevalence in ungulates of the Cetartiodactyla than in those of the Perissodactyla, with cattle (29%) being the most commonly infected farm animal.

**Conclusions:**

Overall, the investigated domestic ungulates are considered potential sources of *Cryptosporidium* contamination in the environment. Control measures should be developed to reduce the occurrence of *Cryptosporidium* infection in these animals. Furthermore, literature on wild populations of the named ungulate species revealed a widespread presence and potential reservoir function of wildlife.

## Background

*Cryptosporidium*, the causative agent of cryptosporidiosis, is an ubiquitous protozoan parasite. It causes gastrointestinal disease in a wide variety of vertebrate hosts, including ungulates of the orders Artiodactyla and Perissodactyla, as well as humans. Several *Cryptosporidium* species are known to be zoonotic with animals as major reservoirs [1]. In resource-limited settings, cryptosporidiosis is a leading cause of diarrhoeal death in children younger than five years across the globe, only second to rotaviral enteritis [2]. Cryptosporidiosis is also a significant contributor to health care cost in developed countries. It is estimated that in the USA 748,000 cases of human cryptosporidiosis occur annually [3]. Residents of and travelers to developing countries may be at greater risk of infection due to poor water treatment and food sanitation [4, 5]. Cryptosporidiosis typically induces self-limiting diarrhea in immunocompetent individuals, but the infection can be severe and life-threatening in immunocompromised subjects [6]. It is one of the most important diseases in young ruminants, especially neonatal calves [7, 8]. The clinical presentation of cryptosporidiosis varies from asymptomatic to deadly, leading to important economic losses due to growth retardation, reduced productivity and mortality [9, 10]. Considering that an infected bovine calf can shed up to 1.1 × 10^8^ oocysts per gram of feces at the peak of the infection, cattle (and very likely wild ruminants) are significant contributors of environmental *Cryptosporidium* oocysts [11, 12], causing water-borne [13–15] and food-borne [16, 17] diarrhea outbreaks in humans worldwide. The worldwide annual excretion of *Cryptosporidium* spp. oocysts by livestock has been calculated to be 3.2 × 10^23^ [18], with cattle being the host species causing most environmental contamination. Cattle are able to carry different species including *C. hominis* which implies an associated significant public health risk [19]. In addition, *Cryptosporidium* oocysts are infective at the time they are passed in feces and are highly resilient to a wide range of environmental factors including disinfection and water treatment processes. Moreover, low infection doses are sufficient to cause disease in suitable hosts, e.g. 10‒100 oocysts are described to provoke diarrhea in humans [20, 21].

Over the past few decades, a major subject of debate and controversy in the epidemiology of *Cryptosporidium* is whether, and to what extent, domestic and wildlife species may act as natural reservoirs of human cryptosporidiosis [22, 23]. This is principally due to the fact that the genus *Cryptosporidium* encompasses nearly 40 valid species with marked differences in host range, among which over 10 (mainly *C. hominis*, *C. parvum* and *C. meleagridis*) have been reported in humans [24] with a variety of genotypes being zoonotic [1, 22, 25]. The public health significance of animal cryptosporidiosis varies greatly depending on factors such as geographical variation in prevalence and genotype distribution, seasonality, load of environmental contamination with oocysts and access to surface waters intended for human consumption or recreation [9, 26]. In particular, genotyping data from epidemiological surveys conducted globally indicate that infected calves are the major reservoir for zoonotic *C. parvum* in many areas [26, 27], with lambs, kids and foals being potential additional sources of *C. parvum* infection for humans in some areas of the world [28–31]. Pigs are only sporadically infected with zoonotic *Cryptosporidium* species and are therefore considered minor contributors to the zoonotic transmission of cryptosporidiosis in humans [32]. Adult livestock typically harbor low level and asymptomatic infections but are epidemiologically important as cryptic carriers of the parasite, enabling re-infections at the herd level. Little is known of the molecular epidemiology and transmission cycles of cryptosporidiosis in wild ungulates. However, recent surveys have revealed the presence of *C. parvum* in wild hoofed species including the American mustang (*Equus ferus caballus*) [33], Scottish roe deer (*Capreolus capreolus*) and red deer (*Cervus elaphus*) [34], and Spanish wild boars (*Sus scrofa scrofa*) [35], which may represent a threat to water quality and public health [34].

In the present study, we conducted a systematic review of publications on the prevalence of *Cryptosporidium* infections and *Cryptosporidium* species distribution in domestic and wild ungulates in order to ascertain the extent to which hoofed animals should be considered as relevant reservoirs of human infection.

## Methods

### Search strategy

**Fig. 1:**
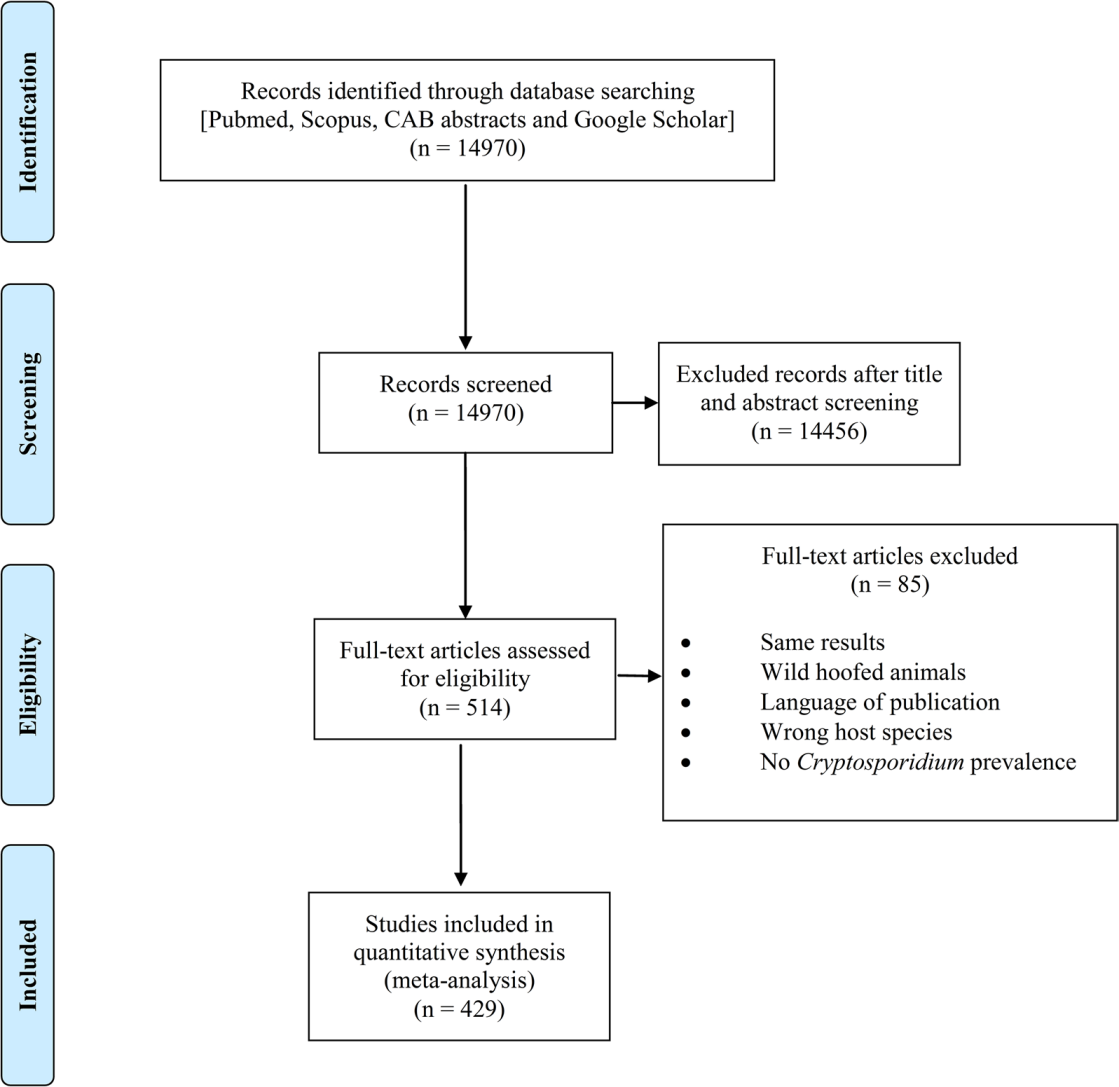
Flow diagram describing the paper selection process according to PRISMA guidelines

To evaluate the prevalence of *Cryptosporidium* infection in hoofed animals, we performed a comprehensive review of literatures (full text or abstracts) published online. English databases including PubMed, Scopus, Science Direct and Cochran were searched for publications related to *Cryptosporidium* infection of animals worldwide, from 1984 to 2016. We used the following MeSH terms alone or in combination: “*Cryptosporidium*” or “cryptosporidiosis” and “prevalence” and “livestock” or “cattle” or “buffaloes” or “sheep” or “pigs” or “camels” or “alpacas” or “horses” or “ruminants” or “wildlife”. To identify additional published articles, we used the PubMed option of “related articles” and checked the reference lists of the original and review articles. The more agricultural and veterinary focused database CAB abstracts was searched using the following search terms: “*Cryptosporidium*” or “cryptosporidiosis” and “prevalence” and “cattle” or “cows” or “calves” or “buffaloes” or “sheep” or “lambs” or “goats” or “kids” or “camels” or “alpacas” or “crias” or “llamas” or “pigs” or “piglets” or “horses” or “foals” or “deer” or “fawns” or “farm animals” or “ruminants” or “livestock” or “wildlife”. A protocol for the literature review was devised (Fig. 1) in accordance with the PRISMA guidelines [36] (Additional file 1: Table S1).

### Inclusion and exclusion criteria

As part of the eligibility for inclusion, titles that suggested the topic *Cryptosporidium* in domestic and wild hoofed animals were selected. The abstracts from the selected reference titles were reviewed by two independent reviewers to determine if the studies met the inclusion criteria and, if so, the entire articles were reviewed in full. If more than one report was published from the same study, only one was included. Exclusion criteria included studies only on human cryptosporidiosis or case reports. Studies on epidemiology of *Cryptosporidium* spp. in groups unrelated to hoofed animals, or studies presenting overall prevalence estimates, where samples were collected from the ground, and data from each animal were not independently retrievable, were also excluded. The language of data collection was limited to English. In order to provide contemporaneous and representative estimates, studies were excluded if they presented data collected prior to 1984. On several occasions, we contacted the authors for the collection of raw data.

### Data extraction and tabulation

A data extraction form was used to collect the following data from each study: first author, year of publication, location of study, period of study, host species, age range, clinical signs (diarrhoeic *versus* non-diarrhoeic), population nature (e.g. domestic, captive or wild), total number of fecal samples, utilized detection method (conventional microscopy, CM; immunofluorescence antibody test, IFA; enzyme-linked immunosorbent assay, ELISA; immunochromatographic test, ICT; quantitative latex agglutination, QLAT; and polymerase chain reaction, PCR), number of *Cryptosporidium-*positive samples and identity of *Cryptosporidium* species and genotypes.

### Retrieving sequences and phylogenetic analyses

To examine the genetic relationships among *Cryptosporidium* spp. (*C. hominis*, *C. felis*, *C. parvum*, *C. erinacei*, *C. xiaoi*, *C. ryanae*, *C. scrofarum*, *C. muris*, *C. andersoni*, *C. ubiquitum*, *C. bovis* and *C. suis*) in ungulates, a phylogenetic tree was constructed using the program Splits Tree v.4.0 based on the Neighbor-Net method and Median-Joining analysis of sequences of the *18S* rRNA gene [37]. For this purpose, the sequences of the *18S* rRNA gene of these *Cryptosporidium* spp. were retrieved from the GenBank database in the FASTA format. These sequences were initially obtained from various herbivores, including cattle, buffaloes, yaks, camels, goats, sheep and deer, as well as pigs.

### Meta-analysis

A meta-analysis was performed for studies describing *Cryptosporidium* infection prevalence in domestic animals that are common in many parts of the world, i.e. cattle, sheep, goats, buffaloes, horses and pigs. This analysis was performed to enhance knowledge on the potential role of livestock in zoonotic *Cryptosporidium* transmission since these animals feature a close contact to humans. The pooled prevalence of *Cryptosporidium* infection as well as its 95% confidence interval (CI) was calculated for each study. A forest plot was generated to display the summarized results and heterogeneity among the included studies. To ensure comparable sensitivity of tests used in analyzed studies, only results from studies based on PCR as a diagnostic method were included. Studies using PCR methods only for molecular *Cryptosporidium* species/genotype identification but utilizing alternative diagnostic methods to determine prevalence were not included. The heterogeneity was expected in advance and statistical analyses including *I*^2^ and Cochrane’s Q test (with a significance level of *P *< 0.1) were used to quantify these variations. The meta-analysis considering the random effects model [38] was performed using the Stats Direct statistical software (http://www.statsdirect.com).

## Results

The initial database search retrieved 14,970 publications. The screening of these records enabled us to exclude 14,456 studies due to not meeting the inclusion criteria. Altogether, 514 studies were retained for further investigation. During the secondary assessment of these papers, another 85 were excluded because of one of the following reasons: other host species including wild hoofed animals; report of the same results as another paper published by the same author; and language of publication (e.g. Chinese, Spanish, etc.). Papers evaluating cryptosporidiosis in camels, yaks, donkeys, alpacas and llamas were excluded in the secondary analysis of data, as the meta-analysis focused on *Cryptosporidium* infection in cattle, sheep, goats, pigs, buffaloes and horses. Eventually, 429 studies which evaluated *Cryptosporidium* infection during three decades met our eligibility criteria and were retained for analysis (Fig. 1).

Different diagnostic procedures were used for the detection of *Cryptosporidium* oocysts to a varying extent in the different studies. The included publications featured CM examination (*n* = 371), IFA (*n* = 107), ELISA (*n* = 25), ICT (*n* = 9), quantitative latex agglutination (QLAT) (*n* = 1) and polymerase chain reaction (PCR) (*n* = 99) (Additional file 2: Table S2).

In total, 196,638 stool samples from Artiodactyla and Perissodactyla ungulates were evaluated, of which 37,206 (18.9%) subjects were positive for *Cryptosporidium* infection. Among the 196,638 stool samples, 90,744 were associated with the domestic hoofed animals (including camels, yaks, donkeys, alpacas and llamas), displaying a *Cryptosporidium* infection prevalence of 13.6% (*n* = 12,377) (Table 1 and Additional file 2: Table S2).

**Table 1:** Summarized *Cryptosporidium* prevalence data for major domestic farmed animals. Data for wild populations of the given species not included (see for full datasets and other host species in Additional file 2: Table S2)

Host species	Region	No. of studies	Utilized diagnostic methods	Retrieved minimum prevalence (%)	Retrieved maximum prevalence (%)
Buffalo (*Bubalus bubalis*)	Africa	6	CM, PCR	1.3 (CM)	52.0 (CM)
	Asia	16	CM, ICT, PCR	3.6 (CM)	50.0 (CM)
	Australia	2	PCR	13.1 (PCR)	30.0 (PCR)
	Europe	1	ELISA	14.7 (ELISA)	
	South America	2	CM, PCR	9.4 (CM)	48.2 (PCR)
Cattle (*Bos taurus*)	Africa	29	CM, ELISA, PCR	0.5 (CM)	86.7 (CM)
	Asia	74	CM, ICT, IFA, PCR	1.5 (CM)	93.0 (CM)
	Australia	7	CM, IFA, PCR	3.6 (IFA)	73.5 (PCR)
	Europe	60	CM, ELISA, ICT, IFA, PCR, QLAT	0.0 (CM)	71.7 (CM)
	New Zealand	5	CM, IFA	2.6 (IFA)	21.2 (CM)
	North America	29	CM, IFA, PCR	1.1 (IFA)	78.0 (CM)
	South America	11	CM, ICT, PCR	3.0 (CM)	56.1 (CM)
Goat (*Capra hircus*)	Africa	10	CM, ELISA	0.0 (CM)	76.5 (ELISA)
	Asia	15	CM, ICT, IFA	0.0 (IFA)	42.9 (CM)
	Australia	1	PCR	4.4 (PCR)	
	Europe	22	CM, ELISA, IFA	0.0 (CM)	93.0 (IFA)
	North America	3	CM	20.0 (CM)	72.5 (CM)
	South America	3	CM	4.8 (CM)	100 (CM)
Sheep (*Ovis aries*)	Africa	10	CM, ELISA, PCR	1.3 (CM)	41.8 (ELISA)
	Asia	17	CM, ELISA, ICT, PCR	1.8 (CM)	66.6 (CM)
	Australia	7	PCR	2.2 (PCR)	81.3 (PCR)
	Europe	22	CM, IFA, ELISA	1.4 (CM)	100.0 (CM)
	North America	9	CM, IFA, PCR	20.0 (CM)	77.4 (PCR)
	South America	5	CM, PCR	0.0 (CM)	25.0 (PCR)
Pig (*Sus scrofa*)	Africa	5	CM, ELISA, IFA, PCR	13.6 (CM)	44.9 (ELISA)
	Asia	13	CM, IFA, PCR	0.4 (IFA)	55.8 (PCR)
	Australia	3	CM, PCR	0.3 (CM)	22.1 (PCR)
	Europe	13	CM, IFA, PCR	0.1 (CM)	40.9 (IFA)
	North America	6	CM, IFA	2.8 (ns)	19.6 (CM)
	South America	3	CM, PCR	0.0 (CM)	2.2 (PCR)
Horse (*Equus caballus*)	Africa	3	CM, PCR	0.0 (CM)	2.9 (PCR)
	Asia	7	CM, PCR	2.7 (PCR)	37.0 (CM)
	Europe	10	CM, ELISA, IFA, PCR	3.4 (PCR)	25.0 (IFA)
	New Zealand	2	CM	18.0 (CM)	83.3 (CM)
	North America	6	CM, IFA, PCR	0.0 (IFA/PCR^a^)	17.0 (IFA)
	South America	7	CM	0.0 (CM)	100.0 (CM)

^a^Multiple studies revealed the same prevalence data

*Abbreviation*: ns, not stated

All subsequent analyses included only the studies that focused on *Cryptosporidium* infection in cattle, sheep, goats, pigs, buffaloes and horses (*n* = 429). Among them, 201 provided data on cattle, 66 on sheep, 55 on goats, 39 on pigs, 37 on horses and 28 on buffaloes (Additional file 2: Table S2).

A total of 105,894 samples from 245 studies on common livestock, defined as cattle, sheep, goats, pigs, horses and buffaloes, were examined for *Cryptosporidium* infection, with 24,829 (23.4%) being positive for *Cryptosporidium* spp. using CM and PCR methods. Most of the studies were conducted on cattle (*n* = 163) and sheep (*n* = 46).

The pooled prevalence rates using the CM method were 22.5% (95% CI: 19.6–25.6%), 20.7% (95% CI: 15.2–26.8%), 18.7% (95% CI: 12.36–26.2%), 15.5% (95% CI: 10.5–21.3%), 13.8% (95% CI: 6.6–22.9%) and 18.6% (95% CI: 11.1–27.4%) for cattle, sheep, goats, pigs, horses and buffaloes, respectively (Table 2). The pooled prevalence rates using the PCR method were 29.1% (95% CI: 23.1–35.6%), 24.4% (95% CI: 16.4–33.4%), 8.2% (95% CI: 3.7–14.3%), 22.6% (95% CI: 13.7–33%), 4.7% (95% CI: 2–8.4%) and 26.0% (95% CI: 12.2–42.8%) for cattle, sheep, goats, pigs, horses and buffaloes, respectively (Table 2). Analysis of available data by regions (continents and New Zealand) showed a moderate geographical variation of observed prevalence (Table 1). Although diagnostic tests varied among regions, the observed prevalence mostly fell within the 5–30% range (Table 2). Regarding cattle, a considerably lower maximum prevalence was seen in New Zealand compared to other regions. *Cryptosporidium* prevalence in goat tended to be lower in Asia; however, only one study was available for Australia. For sheep it was the highest in the regions with most intensive sheep production, i.e. Australia, Europe and North America (Table 1). *Cryptosporidium* prevalence in pigs was the highest in Asia, Africa and Europe. In horses, studies in South America reported the highest *Cryptosporidium* prevalence.

**Table 2:** Statistical analysis of *Cryptosporidium* infection prevalence in domestic ungulates using CM and PCR methods

Method/host	CM						PCR					
	Pooled (%)	OR (95% CI)	Heterogeneity			Publication bias	Pooled (%)	OR (95% CI)	Heterogeneity			Publication bias
			Q statistic	*df*	I^2^ (%)	Egger bias (*P-*value)			Q statistic	*df*	I^2^ (%)	Egger bias (*P-*value)
Cattle	22.5	19.6–25.6	11,038.9	127	98.8	10.51 (*P *< 0.0001)	29.1	23.1–35.6	1591.1	34	97.9	11.52 (*P *< 0.0001)
Sheep	20.7	15.2–26.8	1391.9	30	97.8	6.77 (*P* = 0.0086)	24.4	16.4–33.4	916.7	14	98.5	8.18 (*P* = 0.014)
Goat	18.7	12.36–26.2	1852.1	28	98.5	9.01 (*P* = 0.0004)	8.2	3.7–14.3	11.2	2	82.2	–
Pig	15.5	10.5–21.3	1545.4	21	98.6	12.42 (*P* = 0.0485)	22.6	13.7–33.0	99.8	5	95.0	2.36 (*P* = 0.6452)
Horse	13.8	6.6–22.9	621.6	16	97.4	6.71 (*P* = 0.0002)	4.7	2.0–8.4	22.5	4	82.3	3.67 (*P* = 0.0452)
Buffalo	18.6	11.1–27.4	991.4	17	98.3	8.76 (*P* = 0.0004)	26.0	12.2–42.8	152.4	4	97.4	9.28 (*P* = 0.1434)

**Fig. 2:**
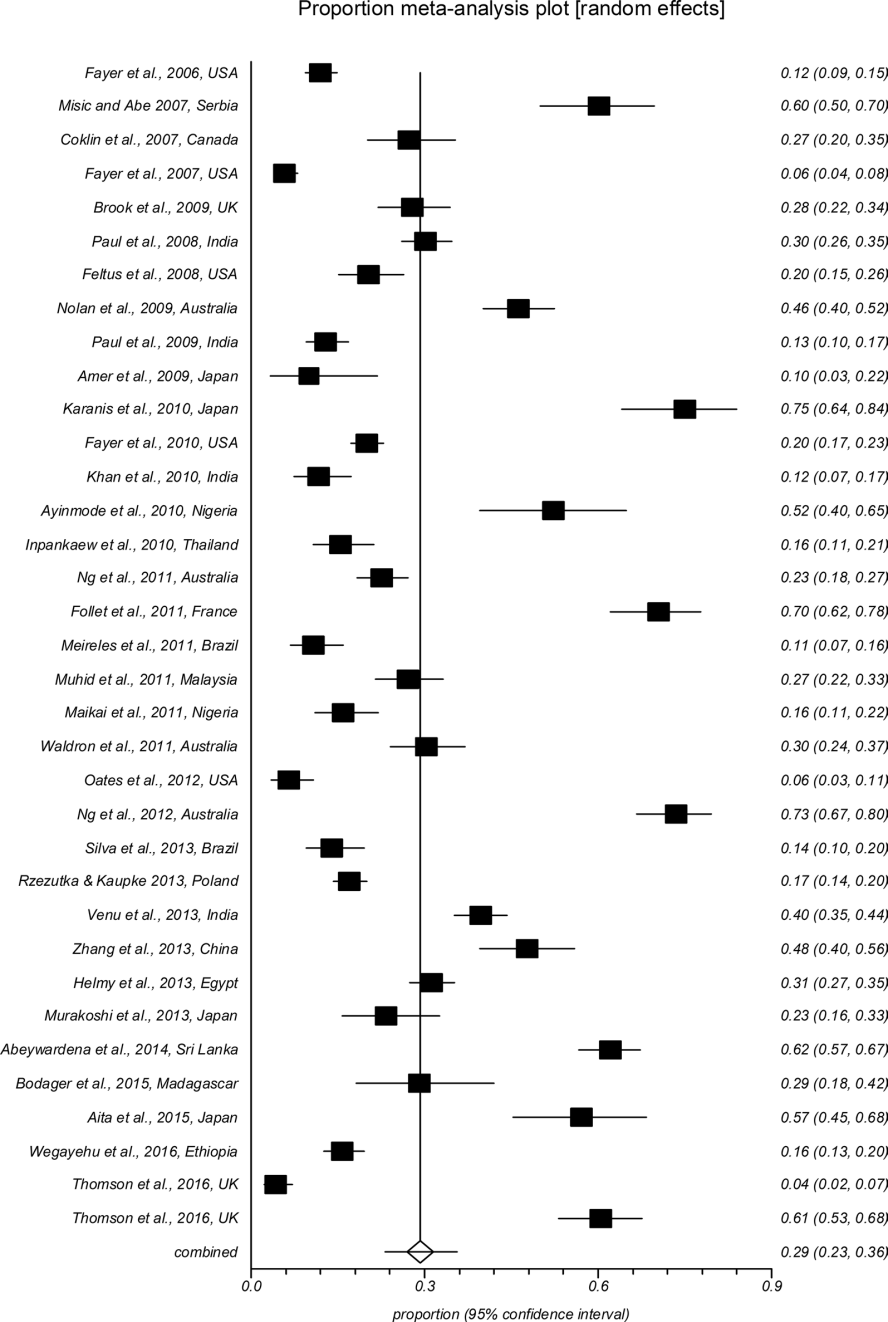
Forest plot of prevalence of *Cryptosporidium* spp. infection in cattle using molecular methods (first author, year and country)

**Fig. 3:**
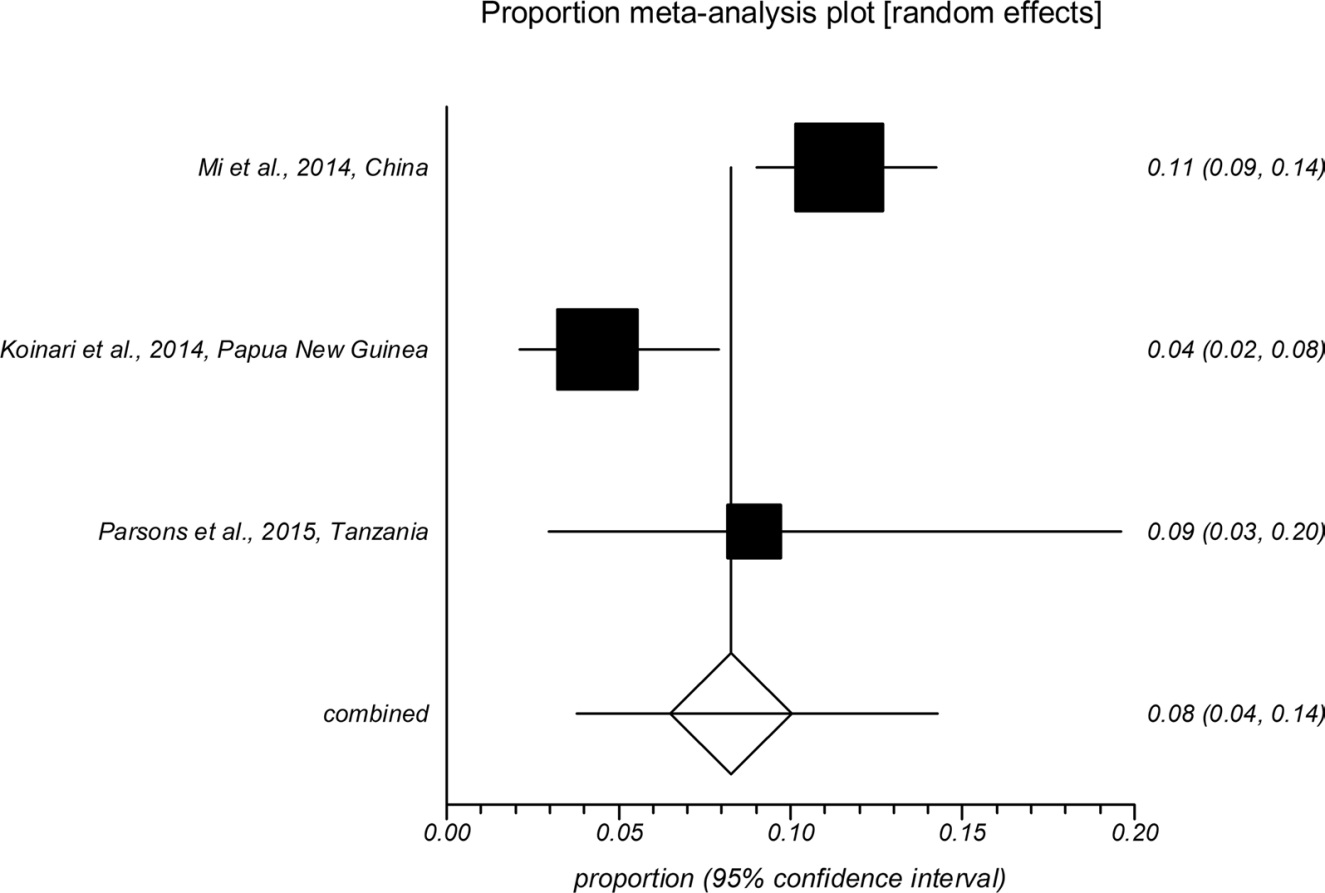
Forest plot of prevalence of *Cryptosporidium* spp. infection in goats using molecular methods (first author, year and country)

**Fig. 4:**
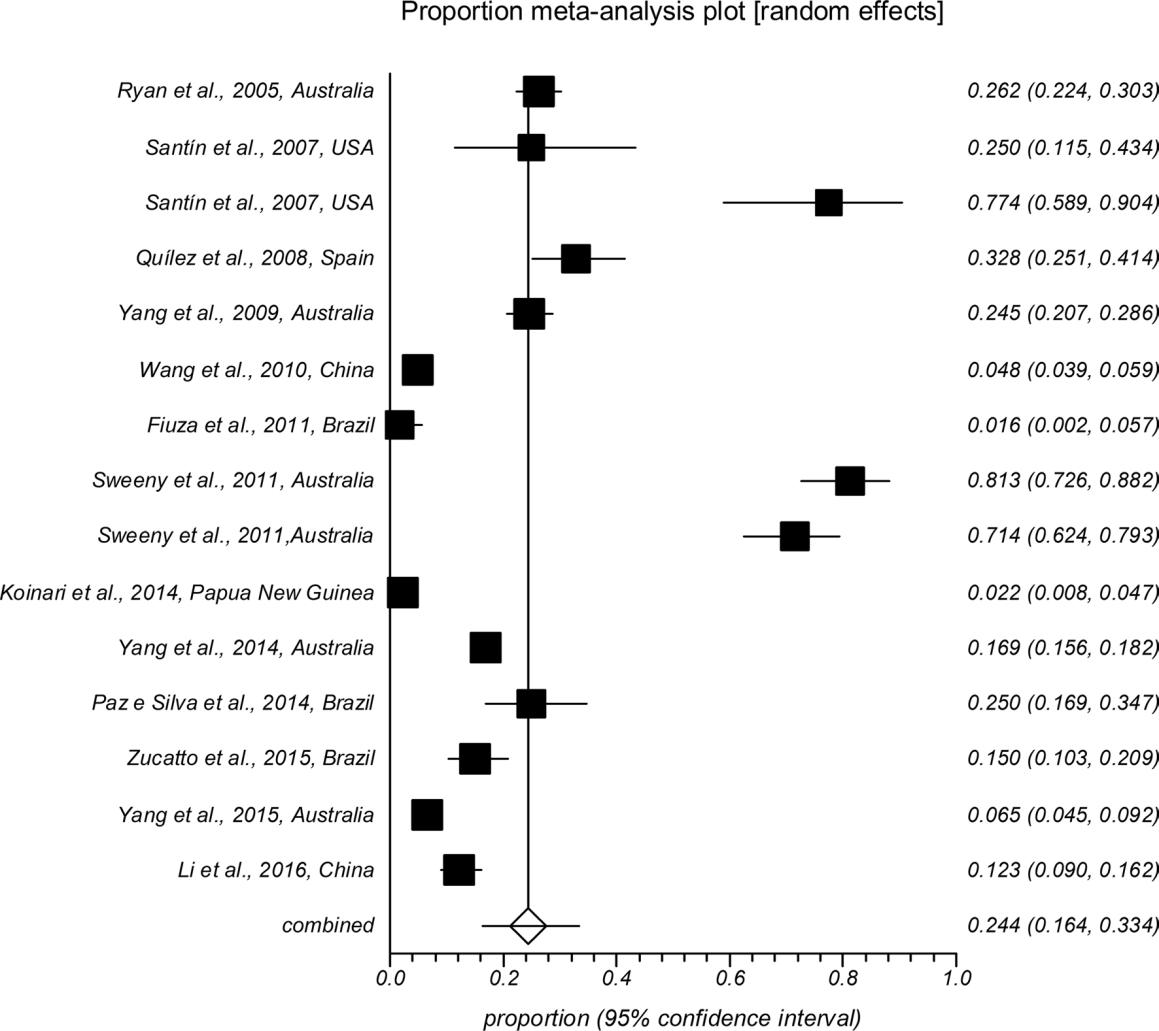
Forest plot of prevalence of *Cryptosporidium* spp. infection in sheep using molecular methods (first author, year and country)

**Fig. 5:**
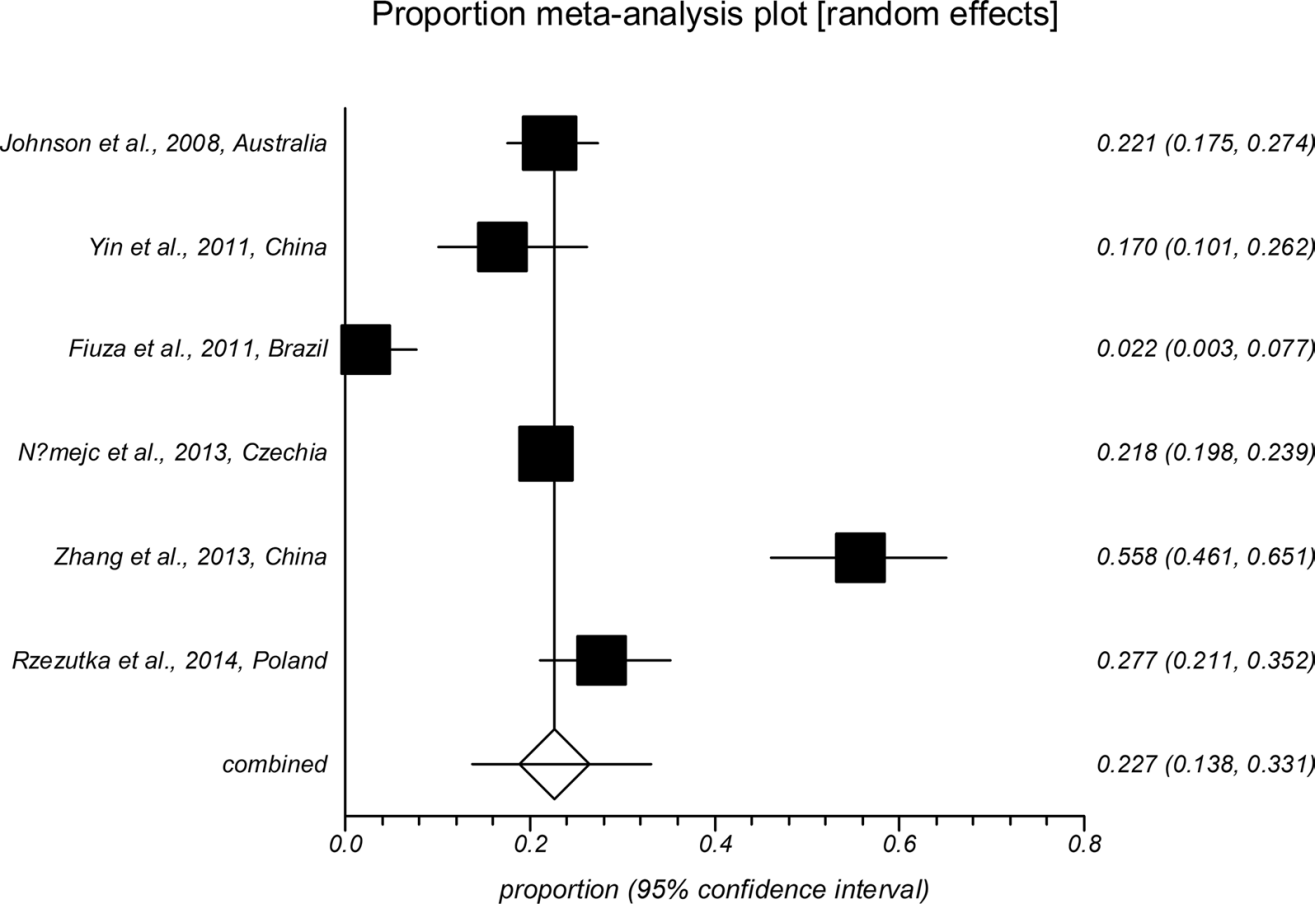
Forest plot of prevalence of *Cryptosporidium* spp. infection in pigs using molecular methods (first author, year and country)

**Fig. 6:**
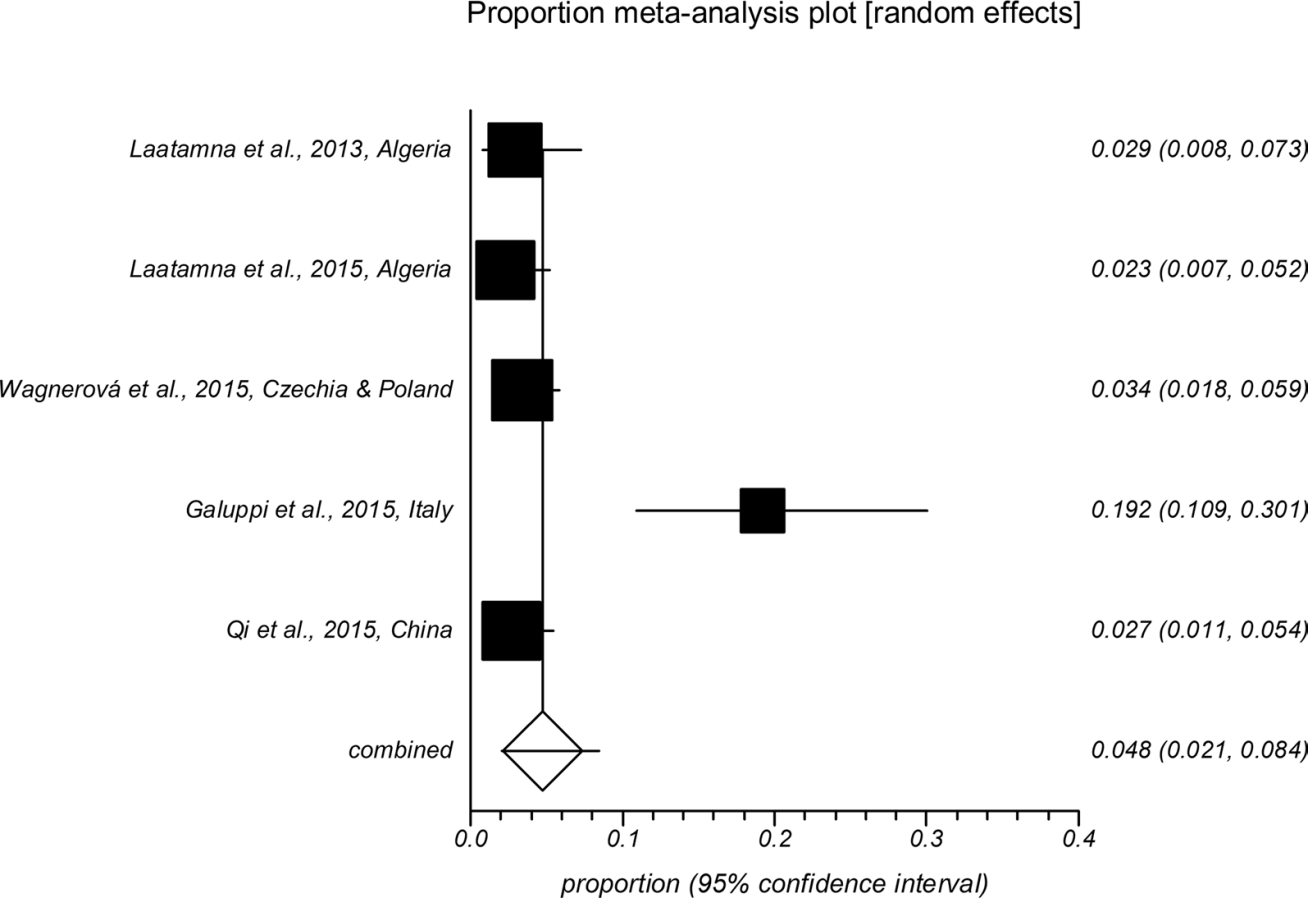
Forest plot of prevalence of *Cryptosporidium* spp. infection in horses using molecular methods (first author, year and country)

**Fig. 7:**
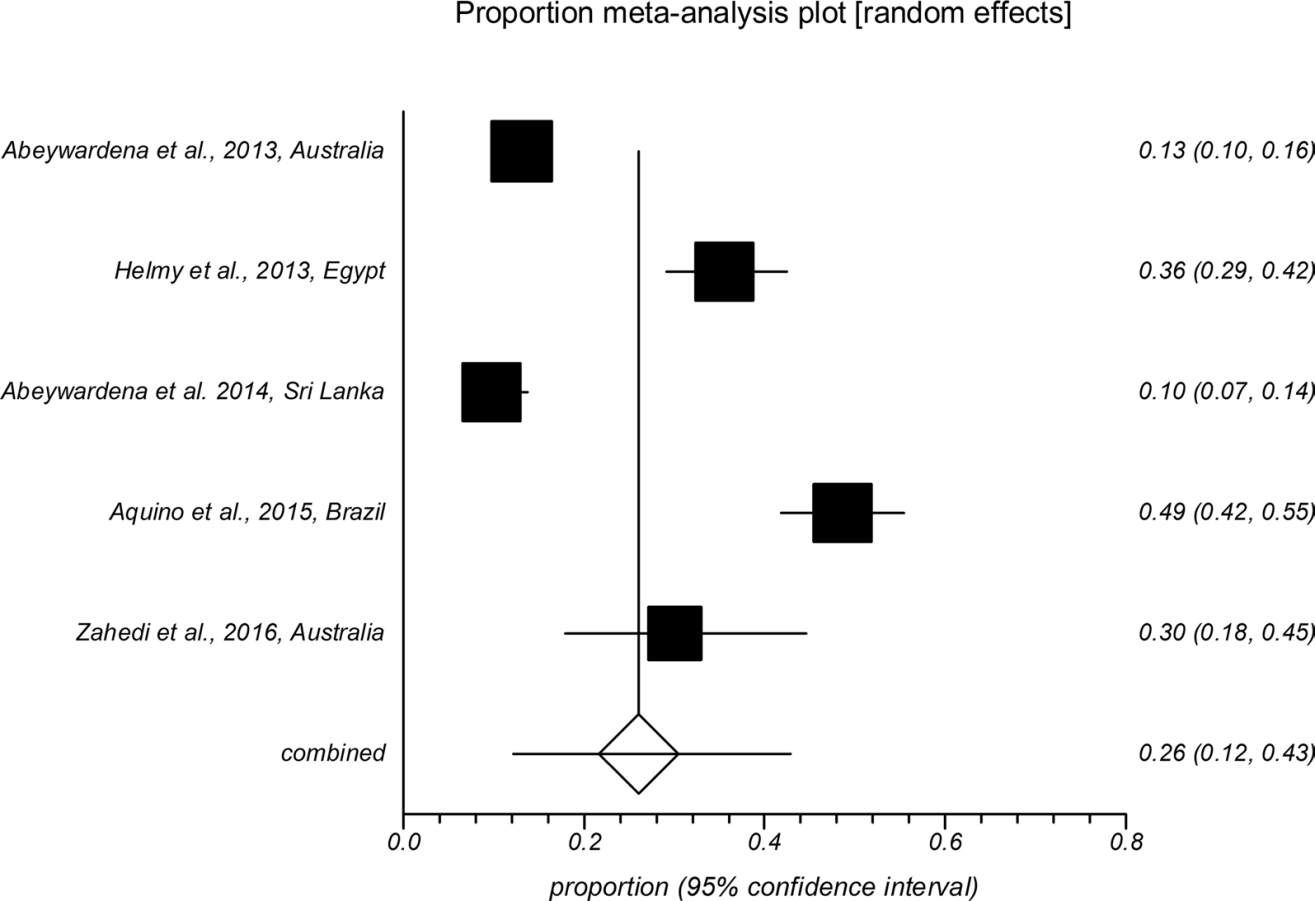
Forest plot of prevalence of *Cryptosporidium* spp. infection in buffaloes using molecular methods (first author, year and country)

The forest plot diagrams of prevalence of *Cryptosporidium* infection in domestic hoofed animals derived from studies using a PCR method are shown in Figs. 2, 3, 4, 5, 6, 7. As forest plots show, there is a considerable variation of study numbers and observed prevalence in a given host species within each defined geographical region, even if only studies based on PCR methodology are included. Considering a wider range of studies, i.e. studies that use either CM or PCR (Table 2), cattle are most commonly infected globally while horses feature the lowest *Cryptosporidium* prevalence.

**Fig. 8:**
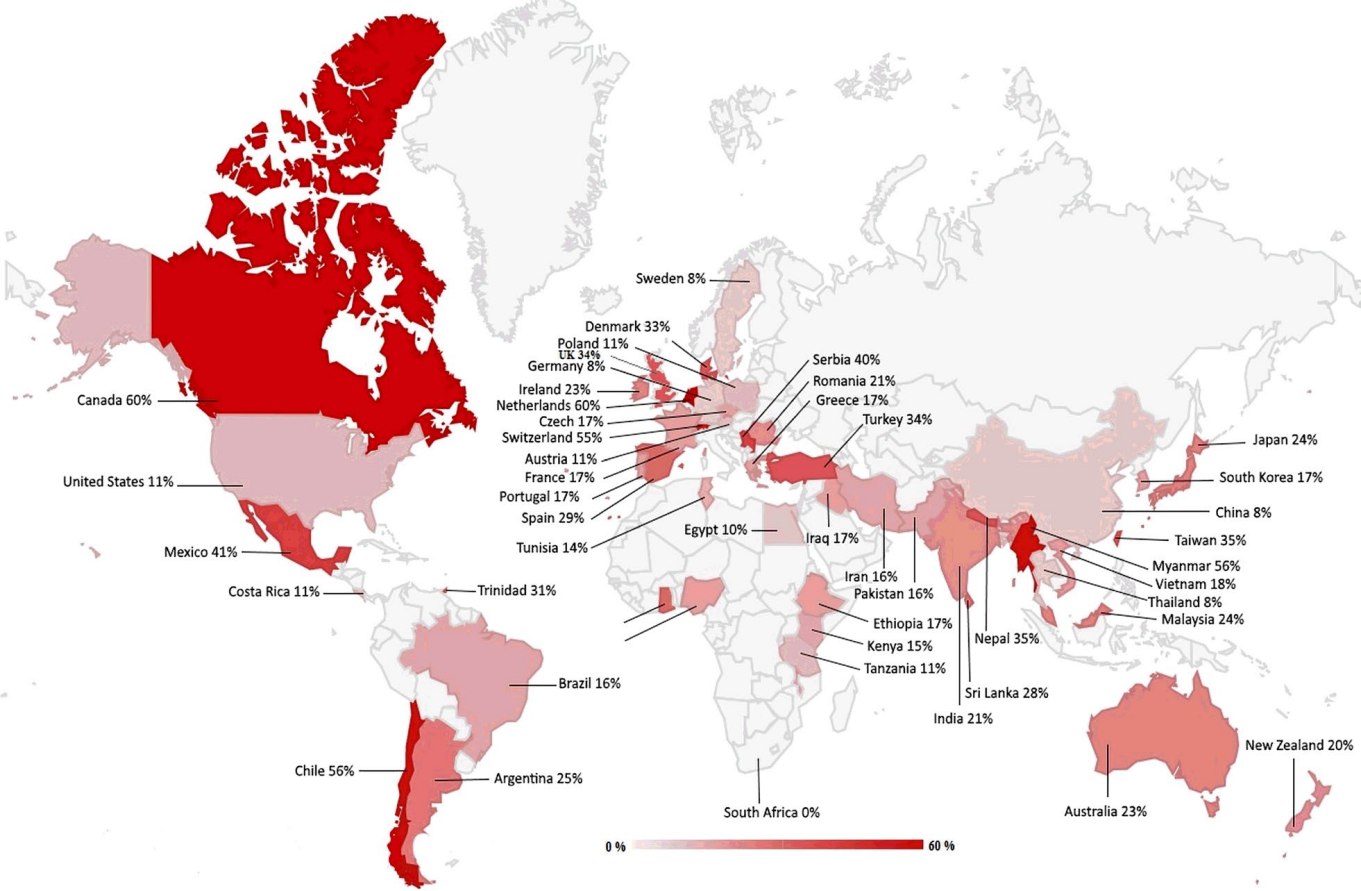
Overall prevalence of *Cryptosporidium* in different geographical regions in the world. The prevalence in each country was determined from conventional microscopy data in farmed animals (cattle, sheep, goats, pigs, horses and buffaloes)

The highest and lowest prevalence rate of *Cryptosporidium* infection in domestic hoofed animals was observed in America (26%) and Africa (14%) continents, respectively (Table 3, Fig. 8). Among 53 countries with data, Canada (60%) showed the highest infection rate whereas China, Thailand and Germany (8%) had the lowest infection rate (Table 3, Fig. 8).

**Table 3:** The prevalence of *Cryptosporidium* infection in terrestrial ungulates (cattle, sheep, goat, pig, horse and buffalo) using conventional microscopic methods. Data are presented separately by continent and country

Continent	Country	Prevalence, pooled proportion (95% CI) (%)
Africa (43 studies; 17,424 samples)	Egypt	10 (4.44–19.32)
	Ethiopia	17 (7.15–30.13)
	Ghana	29^a^
	Kenya	15 (10.72–21.30)
	Malawi	18 (10.48–28.78)
	Nigeria	17 (13.07–22.33)
	South Africa	0.5^a^
	Tanzania	11 (1.59–29.29)
	Tunisia	14 (2.09–44.93)
	Total prevalence in Africa: 14 (11.12–18.31)	
America (37 studies; 15,860 samples)	Argentina	25 (18.83–33.58)
	Brazil	16 (5.82–30.23)
	Canada	60 (23.32–91.14)
	Chile	56^a^
	Costa Rica	11^a^
	Mexico	41 (31.81–52.23)
	Trinidad	32 (6.47–67.24)
	USA	11 (2.84–24.39)
	Total prevalence in America: 26 (18.41–34.67)	
Asia (90 studies; 37,458 samples)	Bangladesh	9 (2.93–20.36)
	China	8 (5.62–12.95)
	India	21 (16.02–28.47)
	Iran	16 (11.96–20.68)
	Iraq	17 (11.36–25.23)
	Japan	24 (0.02–72.52)
	Malaysia	24 (8.43–46.55)
	Myanmar	56^a^
	Nepal	35 (28.81–43.45)
	Pakistan	16 (9.05–25.96)
	South Korea	17 (11.53–23.57)
	Sri Lanka	28^a^
	Taiwan	35 (32.44–38.15)
	Thailand	8 (3.08–17.41)
	Vietnam	18^a^
	Total prevalence in Asia: 17 (14.94–20.30)	
Australia (4 studies; 923 samples)	Australia	23 (0.00–71.85)
	New Zealand	20 (15.42–25.92)
	Total prevalence in Australia: 21 (7.28–40.02)	
Europe (71 studies, 34,229 samples)	Austria	11^a^
	Czech Republic	17 (9.87–27.11)
	Denmark	33 (14.90–55.60)
	France	17 (2.56–41.08)
	Germany	8 (3.62–48.31)
	Greece	17 (9.87–27.11)
	Ireland	23 (3.84–52.25)
	Netherlands	60^a^
	Poland	11 (3.62–21.85)
	Portugal	17^a^
	Romania	21 (15.02–27.97)
	Serbia	40 (31.95–49.48)
	Spain	29 (19.80–39.75)
	Sweden	8^a^
	Switzerland	55^a^
	Turkey	34 (19.82–50.61)
	UK	34 (0.59–85.50)
	Total prevalence in Europe: 23 (20.37–27.68)	

^a^One study was performed in these countries

**Fig. 9:**
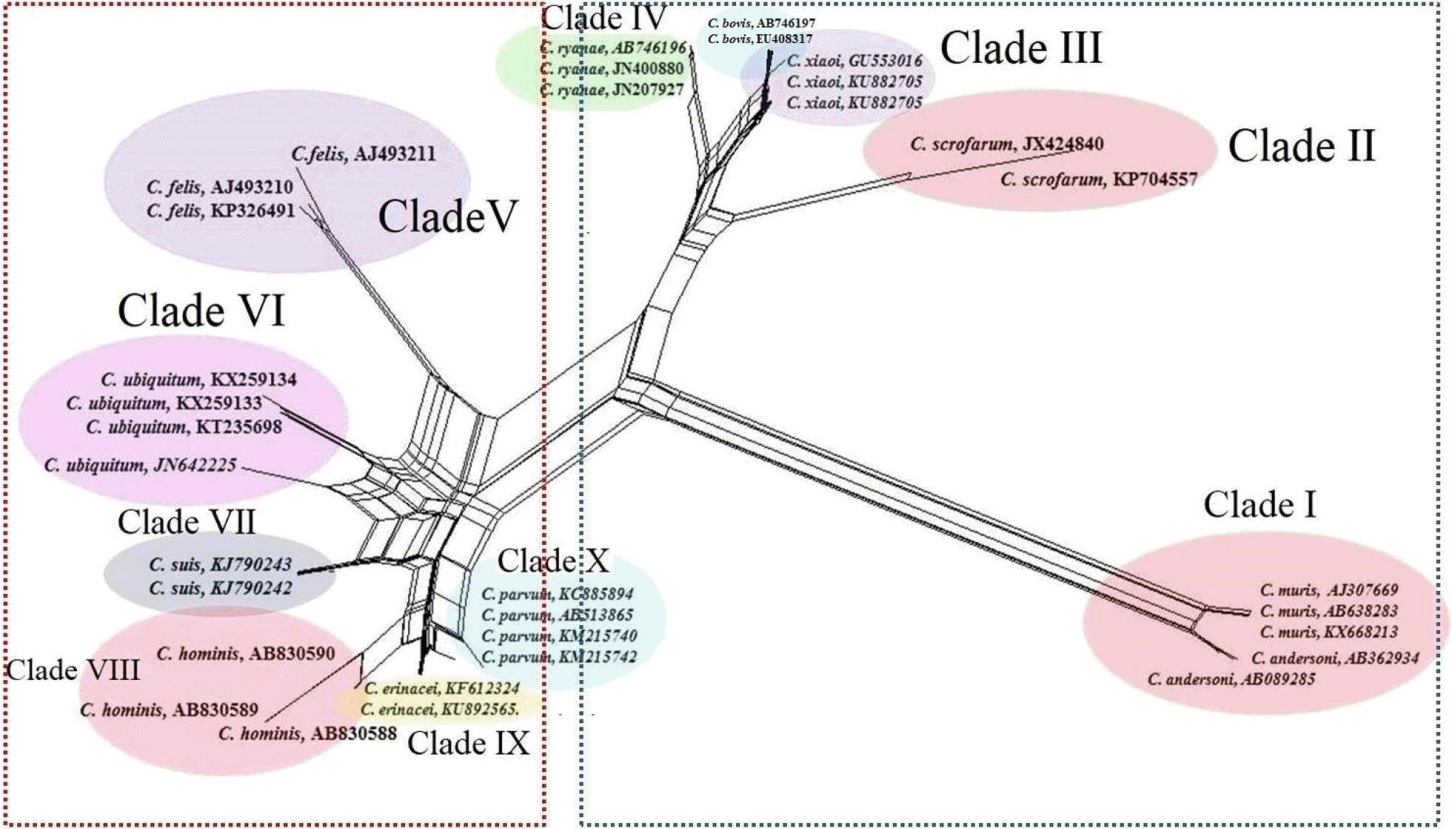
The phylogeny of *Cryptosporidium* spp

The distribution of *Cryptosporidium* species/genotypes by host and geographical region is summarized in Table 4. *Cryptosporidium parvum* (monoinfections 4172/10,583; 39.4%) and *C. andersoni* (monoinfections 1992/10,583; 18.8%) were the most commonly detected *Cryptosporidium* species (Table 4). A phylogenetic network was constructed based on sequences of *Cryptosporidium* spp. (Fig. 9) using the Neighbor-Net method. On the basis of this phylogenetic analysis, 10 clades (I, II, III, IV, V, VI, VII, VIII, IX and X) containing 12 *Cryptosporidium* spp. were identified (Fig. 9). Interestingly, *C. andersoni* and *C. muris* were placed together in Clade I, and *C. xiaoi* and *C. bovis* were both placed in Clade III. It further demonstrated a pairwise sister relationship between clades III and IV (clustering *C. xiaoi*, *C. bovis*, and *C. ryanae*), VI and VII (containing *C. ubiquitum* and *C. suis*) and VIII and IX (containing *C. hominis* and *C. erinacei*), respectively. Interestingly, the result of the phylogenetic analysis indicated that clades II (*C. scrofarum*), III (*C. bovis* and *C. xiaoi*) and IV (*C. ryanae*) could have originated from a common ancestor. The distribution of *Cryptosporidium* spp. in a wide range of domestic and wild ungulates is presented in Table 4. The *C. parvum* is the most common genotype in cattle (54.1%), goats (42.1%) and horses (40.2%), followed by *C. ryanae* in buffaloes (66.6%), *C. suis* in pigs (54.1%), and *C. xiaoi* in sheep (48.9%). In terms of transmission dynamics and clinical importance of zoonotic *Cryptosporidium* spp., *C. hominis*, *C. parvum*, *C. andersoni*, *C. bovis* and *C. ubiquitum* were identified in sheep/goats, cattle/goats/horses/pigs/sheep, cattle/camels/sheep/yaks, buffaloes/cattle/sheep/pigs/red deer and alpacas/buffaloes/cattle/goats/impalas/sheep/red deers, respectively (Table 4).

**Table 4:** Worldwide occurrence of *Cryptosporidium* species or genotypes in selected domestic and wild populations of ungulate species; where applicable, available data are summarized from different sources per country

Host	Country	No. of isolates	No. of *Cryptosporidium* species/genotypes		Reference
			Monoinfection (*n*)	Mixed infection (*n*)	
Alpaca	Peru	3	*C. parvum* (2); *C. ubiquitum* (1)	*–*	Gómez-Couso et al. [51]
Alpaca	UK	9	*C. parvum* (9)	*–*	Twomey et al. [52]; Wessels et al. [53]
Bison	Portugal	1	*C. tyzzeri* (1)	*–*	Alves et al. [54]
Boar	Czech Republic	32	*C. suis* (13); *C. scrofarum* (7)	*C. suis* + *C. scrofarum* (12)	Němejc et al. [55]
Buffalo	Egypt	70	*C. parvum* (41); *C. ryanae* (17); *C. bovis* (2)	*C. parvum* + *C. ryanae* (7); *C. parvum* + *C. bovis* (3)	Amer et al. [56]; Helmy et al. [57]; Mahfouz et al. [58]; Ibrahim et al. [59]
Buffalo	South Africa	2	*C. ubiquitum* (1); *C. bovis* (1)	*–*	Abu Samra et al. [60]
Buffalo	Australia	72	*C. parvum* (9); *C. ryanae* (58); *C. scrofarum* (1); *C. bovis* (4)	*–*	Abeywardena et al. [61]; Zahedi et al. [62]
Buffalo	Italy	6	*C. parvum* (6)	*–*	Caccio et al. [63]
Buffalo	Brazil	63	*C. parvum* (1); *C. ryanae* (60); unknown genotype (2)	*–*	Aquino et al. [64]
Camel	China	3	*C. andersoni* (3)	*–*	Wang et al. [65]; Liu et al. [66]
Cattle	Egypt	238	*C. parvum* (146); *C. andersoni* (7); *C. ryanae* (35); *C. bovis* (15)	*C. parvum* + *C. ryanae* (15); *C. parvum* + *C. bovis* (10); *C. parvum* + *C. andersoni* (3); *C. ryanae* + *C. bovis* (7)	Amer et al. [56]; Helmy et al. [57]; Mahfouz et al. [58]; Ibrahim et al. [59]
Cattle	Ethiopia	71	*C. andersoni* (54); *C. ryanae* (3); *C. bovis* (14)	*–*	Wegayehu et al. [67]
Cattle	Kenya	27	*C. parvum* (17); *C. andersoni* (3); *C. ryanae* (6); *C. ubiquitum* (1)	*–*	Szonyi et al. [68]; Kangʼethe et al. [69]
Cattle	Madagascar	17	*C. suis* (17)	*–*	Bodager et al. [70]
Cattle	Nigeria	65	*C. andersoni* (5); *C. ryanae* (13); *C. bovis* (32)	*C. ryanae* + *C. bovis* (11); *C. bovis* + *C. andersoni* (4)	Ayinmode et al. [71]; Maikai et al. [72]
Cattle	South Africa	6	*C. parvum* (1); *C. andersoni* (2); *C. ubiquitum* (3)	*–*	Abu Samra et al. [60]; Abu Samra [73]
Cattle	Tunisia	7	*C. parvum* (7)	*–*	Soltane et al. [74]
Cattle	Zambia	45	*C. parvum* (29); *C. ubiquitum* (1); *C. bovis* (15)	*–*	Geurden et al. [75]
Cattle	China	299	*C. parvum* (69); *C. andersoni* (100); *C. ryanae* (19); *C. bovis* (89)	*C. parvum* + *C. bovis* (6); *C. parvum* + *C. ryanae* (4); *C. parvum* + *C. andersoni* (3); *C. bovis* + *C. ryanae* (9)	Wang et al. [76, 77]; Huang et al. [78]
Cattle	India	21	*C. parvum* (6); *C. andersoni* (3); *C. ryanae* (3); *C. bovis* (8); *C. occultus* (1)	*–*	Khan et al. [79]
Cattle	Iran	54	*C. parvum* (50); *C. andersoni* (4)	*–*	Meamar et al. [80]; Fotouhi et al. [81]; Pirestani et al. [82]
Cattle	Israel	61	*C. parvum* (61)		Tanriverdi et al. [83]
Cattle	Japan	33	*C. parvum* (32); *C. bovis* (1)		Karanis et al. [84]
Cattle	Malaysia	14	*C. parvum* (11); *C. ryanae* (3)		Halim et al. [85]
Cattle	Australia	439	*C. parvum* (297); *C. andersoni* (20); *C. ryanae* (30); *C. bovis* (72); *C. hominis* (3)	*C. parvum* + *C. bovis* (12); *C. parvum* + *C. ryanae* (4); *C. bovis* + *C. ryanae* (1)	Waldron et al. [86]; Nolan et al. [87]; Ferguson et al. [88]; Ng et al. [89]; McCarthy et al. [90]; O’Brien et al. [91]; Ralston et al. [92]
Cattle	New Zealand	127	*C. parvum* (85); *C. bovis* (42)	*–*	Learmonth et al. [93]; Grinberg et al. [94]; Al-Mawly et al. [95]
Cattle	Belgium	114	*C. parvum* (105); *C. suis* (1); *C. bovis* (8)		Geurden et al. [96]
Cattle	Czech Republic	2019	*C. parvum* (699); *C. andersoni* (1315); *C. bovis* (5)	*–*	Kvac et al. [97]; Kvac et al. [98]; Ondrackova et al. [99]
Cattle	Denmark	244	*C. parvum* (100); *C. andersoni* (59); *C. ryanae* (11); *C. bovis* (57); *C. occultus* (3); unknown genotype (4)	*C. parvum* + *C. andersoni* (10)	Langkjaer et al. [100]; Enemark et al. [101]
Cattle	France	91	*C. parvum* (32); *C. ryanae* (14); *C. ubiquitum* (1); *C. bovis* (11)	*C. parvum* + *C. ryanae* (12); *C. parvum* + *C. bovis* (11); *C. ryanae* + *C. bovis* (8); *C. parvum* + *C. ryanae* + *C. parvum* (2)	Follet et al. [102]
Cattle	Hungary	22	*C. parvum* (21); *C. ryanae* (1)	*–*	Plutzer et al. [103]
Cattle	UK (Northern Ireland)	224	*C. parvum* (213); *C. ryanae* (3); *C. bovis* (8)	*–*	Thompson et al. [104]
Cattle	Italy	101	*C. parvum* (101)	*–*	Duranti et al. [105]
Cattle	Poland	113	*C. parvum* (36); *C. andersoni* (17); *C. ryanae* (8); *C. bovis* (52)	*–*	Rzeżutka & Kaupke [106]
Cattle	Portugal	82	*C. parvum* (82)	*–*	Mendonca et al. [107]
Cattle	Romania	65	*C. parvum* (65)	*–*	Imre et al. [108]
Cattle	Scotland	411	*C. parvum* (409); *C. hominis* (2)	*–*	Smith et al. [109]
Cattle	Serbia	62	*C. parvum* (62)	*–*	Misic & Abe [110]
Cattle	Spain	267	*C. parvum* (255); *C. andersoni* (1); *C. bovis* (4); *C. felis* (4); unknown genotype (3)	*–*	Mendonca et al. [107]; Quilez et al. [111]; Cardona et al. [112]
Cattle	Sweden	359	*C. parvum* (33); *C. andersoni* (4); *C. ryanae* (40); *C. bovis* (262); *C. ubiquitum* (1)	*C. parvum* + *C. bovis* (13); *C. parvum* + *C. ryanae* (6)	Silverlas et al. [113]; Silverlas et al. [114]; Silverlas et al. [115]; Bjorkman et al. [116]
Cattle	Switzerland	81	*C. parvum* (81)	*–*	Uhde et al. [117]
Cattle	Turkey	15	*C. parvum* (15)	*–*	Tanriverdi et al. [83]
Cattle	UK	306	*C. parvum* (240); *C. andersoni* (20); *C. ryanae* (1); *C. bovis* (31)	*C. parvum* + *C. ryanae* + *C. bovis* (1); *C. ryanae* + *C. bovis* (5); *C. parvum* + *C. bovis* (1); *C. parvum* + *C. ryanae* (1); *C. andersoni* + *C. ryanae* (6)	Thompson et al. [104]; Brook et al. [118]; Featherstone et al. [119]; Moriarty et al. [120]; Smith et al. [121]
Cattle	Canada	134	*C. parvum* (51); *C. andersoni* (38); *C. ryanae* (11); *C. bovis* (34)	*–*	Coklin et al. [122]; Coklin et al. [123]; Budu-Amoako et al. [124]; Budu-Amoako et al. [125]
Cattle	USA	698	*C. parvum* (240); *C. andersoni* (203); *C. ryanae* (83); *C. bovis* (171); *C. suis* (1)		Santín et al. [126]; Fayer et al. [127–129]; Szonyi et al. [130]
Cattle	Brazil	57	*C. parvum* (15); *C. andersoni* (33); *C. ryanae* (4); *C. bovis* (5)		Meireles et al. [131]; Sevá et al. [132]; Silva et al. [133]
Giraffe	Czech Republic	1	*C. muris* (1)	*–*	Kodádková et al. [134]
Goat	Tanzania	5	*C. xiaoi* (5)	*–*	Parsons et al. [135]
Goat	Zambia	1	*C. parvum* (1)	*–*	Goma et al. [136]
Goat	China	44	*C. andersoni* (16); *C. ubiquitum* (24); *C. xiaoi* (4)	*–*	Wang et al. [137]
Goat	Papua New Guinea	10	*C. parvum* (2); *C. hominis* (6); *C. xiaoi* (1); rat genotype II (1)	*–*	Koinari et al. [138]
Goat	Belgium	11	*C. parvum* (11)		Geurden et al. [139]
Goat	France	31	*C. parvum* (1); *C. ubiquitum* (12); *C. xiaoi* (18)	*–*	Rieux et al. [140]; Paraud et al. [141]
Goat	Greece	14	*C. parvum* (2); *C. ubiquitum* (5); *C. xiaoi* (7)	*–*	Tzanidakis [142]
Goat	Spain	68	*C. parvum* (61); *C. xiaoi* (7)	*–*	Díaz et al. [143]; Díaz et al. [144]
Goat	UK	1	*C. hominis* (1)	*–*	Giles et al. [46]
Horse	Algeria	4	*C. erinacei* (4)	*–*	Laatamna et al. [145]
Horse	China	2	*C. andersoni* (2)	*–*	Liu et al. [146]
Horse	New Zealand	9	*C. parvum* (9)	*–*	Grinberg et al. [31]
Horse	Czech Republic	12	*C. parvum* (1); *C. muris* (9); *C. ryanae* (1); horse genotype (1)	*–*	Wagnerová et al. [33]
Horse	Italy	35	*C. parvum* (5); horse genotype (21)	Horse genotype + *C. parvum* (9)	Galuppi et al. [147]
Horse	UK	3	*C. parvum* (3)	*–*	Smith et al. [121]; Chalmers et al. [148]
Horse	USA	29	*C. parvum* (20); horse genotype (9)	*–*	Wagnerová et al. [][33]; Burton et al. [149]
Impala	South Africa	2	*C. ubiquitum* (2)	*–*	Abu Samra et al. [60]
Mouflon sheep	Czech Republic	1	*C. muris* (1)	*–*	Kotková et al. [150]
Pig	Madagascar	4	*C. parvum* (1); *C. suis* (3)	*–*	Bodager et al. [70]
Pig	Australia	87	*C. scrofarum* (48); *C. suis* (35); *C, bovis* (4)	*–*	McCarthy et al. [90]; [Morgan et al. [151]; Johnson et al. [152]; Ryan et al. [153]
Pig	Czech Republic	1031	*C. parvum* (2); *C. muris* (5); *C. scrofarum* (374); *C. suis* (621)	*C. suis* + *C. scrofarum* (29)	Vitovec et al. [154]; Kváč et al. [155, 156]; Němejc et al. [157]
Pig	Denmark	239	*C. scrofarum* (171); *C. suis* (68)		Langkjaer et al. [100]; Petersen et al. [158]
Pig	Ireland	28	*C. parvum* (2); *C. muris* (1); *C. scrofarum* (11); *C. suis* (14)	*–*	Zintl et al. [32]
Pig	UK	42	*C. parvum* (11); *C. scrofarum* (25); *C. suis* (6)	*–*	Smith et al. [121]; Featherstone et al. [159]
Pig	Brazil	2	*C. scrofarum* (2)	*–*	Fiuza et al. [160]
Red deer	Czech Republic	6	*C. muris* (1); *C. ubiquitum* (5)	*–*	Kotková et al. [150]
Roe deer	Spain	6	*C. ryanae* (3); *C. bovis* (3)	*–*	García-Presedo et al. [161]
Sheep	Egypt	3	*C. xiaoi* (3)	*–*	Mahfouz et al. [58]
Sheep	Tanzania	2	*C. xiaoi* (2)	*–*	Parsons et al. [135]
Sheep	Tunisia	3	*C. bovis* (3)	*–*	Soltane et al. [74]
Sheep	Zambia	6	*C. parvum* (5); *C. ubiquitum* (1)	*–*	Goma et al. [136]
Sheep	China	125	*C. andersoni* (4); *C. ubiquitum* (78); *C. xiaoi* (43)	*–*	Wang et al. [162]; Li et al. [163]
Sheep	Australia	1005	*C. parvum* (78); *C. andersoni* (6); *Sheep genotype I* (7); *C. scrofarum* (8); *C. suis* (2); *C. ubiquitum* (148); *C. hominis* (1); *C. xiaoi* (641); *C. bovis* (66); *C. macropodum* (4); unknown genotype (1)	*C. parvum* + *C. xiaoi* (42); *C. parvum* + *C. ubiquitum* (1)	Sweeny et al. [43]; Yang et al. [164]; Ryan et al. [165]; Yang et al. [166, 167]
Sheep	Papua New Guinea	6	*C. parvum* (4); *C. andersoni* (1); *C. scrofarum* (1)	*–*	Koinari et al. [138]
Sheep	Belgium	9	*C. parvum* (9)		Geurden et al. [139]
Sheep	Greece	10	*C. parvum* (7); *C. ubiquitum* (3)	*–*	Tzanidakis [142]
Sheep	Romania	24	*C. parvum* (20); *C. ubiquitum* (2); *C. xiaoi* (2)	*–*	Imre et al. [168]
Sheep	Scotland	16	*C. parvum* (16)	*–*	Galuppi et al. [147]
Sheep	Spain	57	*C. parvum* (46); *C. ubiquitum* (11)	*–*	Díaz et al. [144, 169]
Sheep	UK	133	*C. parvum* (121); *C. hominis* (2); *C. bovis* (10)	*–*	Mueller-Doblies et al. [28]; Giles et al. [46]; Smith et al. [121]; Pritchard et al. [170]
Sheep	Brazil	42	*C. parvum* (3); *C. ubiquitum* (24); *C. xiaoi* (15)	*–*	Fiuza et al. [171]; Paz e Silva et al. [172]; Zucatto et al. [173]
White-tailed deer	Czech Republic	3	*C. muris* (1); *C. ryanae* (2)		Kotková et al. [150]
Yak	China	158	*C. andersoni* (72); *C. ryanae* (37); *C. bovis* (47); *C. occultus* (2)	*–*	Yang et al. [164]

*Notes: C. suis* (previously known as pig genotype I); *C. scrofarum* (previously known as pig genotype II); *C. ryanae* (previously deer-like genotype); *C. erinacei* (previously described as hedgehog genotype); *C. bovis* (previously bovine genotype B); *C. macropodum* (previously marsupial genotype II); *C. xiaoi* (previously bovis-like genotype); *C. hominis* (synonym: *C. parvum* genotype 1); *C. parvum* (synonym: *C. parvum* genotype 2); *C. ubiquitum* (previously identified as *Cryptosporidium* cervine genotype)

*Abbreviations*: *n*, numbers in parentheses are number of positive samples genotypes for each species or genotype

## Discussion

In this systematic review and meta-analysis, we found that 18.9% of the overall populations of the investigated ungulate species were infected with *Cryptosporidium* spp. Our study showed that although the prevalence of *Cryptosporidium* infection was higher in ungulates of the Cetartiodactyla than in Perissodactyla, the prevalence in the latter was not negligible and needs to be considered in terms of pathogen transmission and cycling. From the data collected and summarized on wild animals (as included in Table 4, and Additional file 2: Table S2), it is obvious that sylvatic cycles play a major role in *Cryptosporidium* transmission. Wild terrestrial ungulates are likely serving as important reservoir for the parasite, and certainly the infection of livestock and humans may occur by contact to wildlife feces. For meta-analysis, worldwide *Cryptosporidium* prevalence and species/genotype identity common livestock species have been scrutinized. Overall, *Cryptosporidium* prevalence in farmed animals is the highest in the Americas and Europe (Table 3) which could be attributed to the intensive farm animal production in these regions. More specifically, considering domestic farm animals, the pooled prevalence of equine *Cryptosporidium* infection was 4.7%, compared to the pooled prevalence of 29.1%, 26.0%, 24.4%, 22.6% and 8.2% in cattle, buffaloes, sheep, pigs and goats, respectively. Regarding the number of studies published for the different geographical regions, our analysis does not support under investigation of certain regions (e.g. Asia) as cause of a detection bias. This reinforces the suggestion that animal production intensity affects the prevalence of *Cryptosporidium* spp. Concentrated animal feeding operations (CAFOs) are most common in cattle and pigs. For example, in the USA, in 2002 more than 71% of all produced beef were derived from operations holding more than 5000 heads of cattle each. It is known that CAFOs pose a major problem due to the high amounts of manure that are released to the environment, facilitating potential pathogen transmission to humans, wildlife and other agricultural operations [39]. Furthermore, pathogen transmission within a CAFO seems much more likely than in more extensive farming systems. Accordingly, a high prevalence of *Cryptosporidium* was observed in animals from countries with many CAFO operations, especially in studies in Asia and Europe, with both regions harboring the majority of the commercial pig raising industry [40]. High prevalences in pigs in Africa may be attributed to the opposite effect of extensive farming with high exposure to environmental contamination. Other host animals displaying a high prevalence, such as buffaloes and sheep, are also generally kept in larger groups on commercial operations. The comparatively low prevalence rates in equines and goats may potentially result from smaller animal groups and free-range nature of the animal management.

Between wild and domestic animals, it appears that *Cryptosporidium* prevalence is lower in wild populations than in farmed populations in the same host species. For example, Zahedi et al. [41] reported *Cryptosporidium* infection rates of 30% in farmed buffalo but 12% in wild buffalo. This suggests that animal density and confinement to the same (contaminated) environment facilitate *Cryptosporidium* transmission in domestic animals, and there is no clear host species disposition in terms of general susceptibility to infection with the genus *Cryptosporidium* despite the observed variation in *Cryptosporidium* infection rates among host species (Table 4).

Cryptosporidiosis in ungulates, especially ruminants, has several economic and health implications. Cryptosporidiosis in neonatal calves can lead to profuse watery diarrhea, loss of appetite, lethargy, dehydration and even death, thus may require costly treatments [42]. Moreover, as shown in sheep and goats, cryptosporidiosis can exhibit long-term effects on the growth of animals [43, 44]. Additionally, infected calves can shed over 1 × 10^10^ oocysts each day, which can survive in the environments for months. The ingestion of very few oocysts can cause infection in susceptible hosts, including humans [23, 45]. It has been shown that the median infection dose of *C. parvum* for humans range from below 10 to over 1000 oocysts [22]. Zoonotic transmission of *Cryptosporidium* spp. can easily occur seasonally from young animals such as bovine calves to humans, frequently as an occupational hazard [45, 46].

Nearly 40 *Cryptosporidium* species have been recognized based on molecular, morphological and biological characteristics of the parasites. Previous studies have shown that four major species are responsible for bovine cryptosporidiosis, namely *C. parvum*, *C. andersoni*, *C. bovis* and *C. ryanae* [1]. We showed that the most prevalent *Cryptosporidium* species in ungulates are *C. parvum* and *C. andersoni*, comprising 39.4% and 18.8% of detected parasites, respectively.

The data also suggest that some *Cryptosporidium* species are shared among ungulate hosts (Table 4). This indicates the occurrence of some inter-species transmission of *Cryptosporidium* spp. among ungulate species, making wildlife an important reservoir for infections in domestic animals. Currently, most data on the distribution of *Cryptosporidium* species and genotypes are available on domestic animal populations. Amazingly, there are clear differences in the distribution of *Cryptosporidium* species within the same host species among geographical regions. For example, studies from Ethiopia and Nigeria indicate that *C. andersoni* and *C. bovis* are the most prevalent species in cattle. In contrast, in countries with concentrated animal feeding operations (CAFO) such as Australia, Iran, Japan and New Zealand, as well as many European and North American countries, *C. parvum* is prevalent in cattle (Table 4). Similarly, alpacas in their region of origin are mostly infected with *C. parvum* and *C. ubiquitum*, while alpacas in the UK only tested positive for *C. parvum* (Table 4). Calves, lambs and goat kids in areas with more human activities can even have *C. hominis* infections [19, 41, 47, 48]. Thus, it might be speculated that husbandry systems and contact to other livestock and humans strongly influence the distribution of *Cryptosporidium* species in an ungulate population.

Our meta-analysis had several limitations. We observed a substantial heterogeneity among the included studies. Heterogeneity in the meta-analyses of prevalence is not uncommon, and the random-effect model implicitly incorporates some of the heterogeneity [49]. Nevertheless, we investigated several factors that can contribute to the observed heterogeneity. The diagnostic method used for the detection of *Cryptosporidium* infection was one of the main confounding variables. For example, the pooled prevalence of bovine *Cryptosporidium* infection was estimated 29.1% using PCR compared to 22.5% using conventional microscopy. This seems to indicate that molecular methods such as PCR are highly sensitive and specific for the detection of *Cryptosporidium* infection, but compared with conventional microscopic methods, they are more expensive and require a higher degree of expertise [50].

There are geographical differences in the estimated pooled prevalence of *Cryptosporidium* infection. The prevalence was highest in the continent of America, followed by Europe, Australia, Asia and Africa. Canada had the highest prevalence among countries. Study design, time of sampling, age of animals, and conditions of keeping animals are other factors that can contribute to the observed heterogeneity in cryptosporidiosis prevalence, in addition to the nature of animal management.

The outcome of our study is probably affected by the publication bias. Publication bias occurs when the results of studies affect the likelihood of their inclusion in the systematic review and meta-analysis [49]. Our systematic review was limited to studies published after 1984 in English. Moreover, many studies did not provide enough information to be included in the meta-analysis.

## Conclusions

Results of the meta-analysis suggest that *Cryptosporidium* infection is highly prevalent in ungulates, especially ruminants. Geographical differences in *Cryptosporidium* prevalence and distribution of *Cryptosporidium* species are seen for most domestic ungulate hosts. These within-host-species differences could be partially attributed to differences in animal management among geographical regions. The highest prevalence in farmed ungulates occurs in America and Europe where CAFO is widely practiced. The major farm animal hosts of *Cryptosporidium* spp. appear to be cattle, buffalo, sheep and pigs. These farm animals are potent reservoirs for a variety of *Cryptosporidium* species. *Cryptosporidium* prevalence is also clearly higher in farmed animals than in wild ungulate populations. Inter-species transmission of *Cryptosporidium* spp. appears to be affected by contact with other host species (humans or other animals) and infection pressure (intensive farming), rendering the investigated ungulate hosts capable of propagating both zoonotic and non-zoonotic *Cryptosporidium* species.

## Supplementary information

**Additional file 1: Table S1.** PRISMA checklist.

**Additional file 2: Table S2.** Worldwide prevalence of *Cryptosporidium* spp. in herbivorous animals.

## Data Availability

Data supporting the conclusions of this article are included within the article and its additional files.

## Abbreviations

CM: conventional microscopy
ELISA: enzyme-linked immunosorbent assay
ICT: immunochromatographic test
PCR: polymerase chain reaction
PRISMA: Preferred Reporting Items for Systematic Reviews and Meta-Analyses
QLAT: quantitative latex agglutination test

## References

[1] Feng Y, Ryan UM, Xiao L (2018). Genetic diversity and population structure of *Cryptosporidium*. Trends Parasitol..

[2] Khalil IA, Troeger C, Rao PC, Blacker BF, Brown A, Brewer TG (2018). Morbidity, mortality, and long-term consequences associated with diarrhoea from *Cryptosporidium* infection in children younger than 5 years: a meta-analysis study. Lancet Glob Health..

[3] Scallan E, Hoekstra RM, Angulo FJ, Tauxe RV, Widdowson M-A, Roy SL (2011). Foodborne illness acquired in the United States—major pathogens. Emerg Infect Dis..

[4] DuPont HL (2016). Persistent diarrhea: a clinical review. JAMA..

[5] Hatam-Nahavandi K, Mohebali M, Mahvi AH, Keshavarz H, Khanaliha K, Tarighi F (2015). Evaluation of *Cryptosporidium* oocyst and *Giardia* cyst removal efficiency from urban and slaughterhouse wastewater treatment plants and assessment of cyst viability in wastewater effluent samples from Tehran, Iran. J Water Reuse Desal..

[6] Marcos LA, Gotuzzo E (2013). Intestinal protozoan infections in the immunocompromised host. Curr Opin Infect Dis..

[7] Cho YI, Han JI, Wang C, Cooper V, Schwartz K, Engelken T (2013). Case–control study of microbiological etiology associated with calf diarrhea. Vet Microbiol..

[8] Meganck V, Hoflack G, Opsomer G (2014). Advances in prevention and therapy of neonatal dairy calf diarrhoea: a systematical review with emphasis on colostrum management and fluid therapy. Acta Vet Scand..

[9] Thompson RA, Palmer CS, O’Handley R (2008). The public health and clinical significance of *Giardia* and *Cryptosporidium* in domestic animals. Vet J..

[10] Santin M (2013). Clinical and subclinical infections with *Cryptosporidium* in animals. N Z Vet J..

[11] Oates SC, Miller MA, Hardin D, Conrad PA, Melli A, Jessup DA (2012). Prevalence, environmental loading, and molecular characterization of *Cryptosporidium* and *Giardia* isolates from domestic and wild animals along the Central California Coast. Appl Environ Microbiol..

[12] Silverlås C, Bosaeus-Reineck H, Näslund K, Björkman C (2013). Is there a need for improved *Cryptosporidium* diagnostics in Swedish calves?. Int J Parasitol..

[13] Karanis P, Plutzer J, Halim NA, Igori K, Nagasawa H, Ongerth J (2007). Molecular characterization of *Cryptosporidium* from animal sources in Qinghai province of China. Parasitol Res..

[14] Efstratiou A, Ongerth JE, Karanis P (2017). Waterborne transmission of protozoan parasites: review of worldwide outbreaks—an update 2011–2016. Water Res..

[15] Hatam-Nahavandi K, Mohebali M, Mahvi AH, Keshavarz H, Najafian HR, Mirjalali H (2016). Microscopic and molecular detection of *Cryptosporidium andersoni* and *Cryptosporidium xiaoi* in wastewater samples of Tehran Province, Iran. Iran J Parasitol..

[16] Budu-Amoako E, Greenwood SJ, Dixon BR, Barkema HW, McClure J (2011). Foodborne illness associated with *Cryptosporidium* and *Giardia* from livestock. J Food Prot..

[17] Ryan U, Hijjawi N, Xiao L (2018). Foodborne cryptosporidiosis. Int J Parasitol..

[18] Vermeulen LC, Benders J, Medema G, Hofstra N (2017). Global *Cryptosporidium* loads from livestock manure. Environ Sci Technol..

[19] Razakandrainibe R, Costa D, Le Goff L, Lemeteil D, Ballet JJ, Gargala G (2018). Common occurrence of *Cryptosporidium hominis* in asymptomatic and symptomatic calves in France. PLoS Negl Trop Dis..

[20] Roberts CL, Morin C, Addiss DG, Wahlquist SP, Mshar PA, Hadler JL (1996). Factors influencing *Cryptosporidium* testing in Connecticut. J Clin Microbiol..

[21] Chappell CL, Okhuysen PC, Sterling CR, DuPont HL (1996). *Cryptosporidium parvum*: intensity of infection and oocyst excretion patterns in healthy volunteers. J Infect Dis..

[22] Ryan U, Fayer R, Xiao L (2014). *Cryptosporidium* species in humans and animals: current understanding and research needs. Parasitoloy..

[23] Thompson R, Ash A (2016). Molecular epidemiology of *Giardia* and *Cryptosporidium* infections. Infect Genet Evol..

[24] Nichols GL, Chalmers RM, Hadfield SJ (2014). Molecular epidemiology of human cryptosporidiosis. *Cryptosporidium*: parasite and disease.

[25] Xiao L (2010). Molecular epidemiology of cryptosporidiosis: an update. Exp Parasitol..

[26] Gong C, Cao XF, Deng L, Li W, Huang XM, Lan JC (2017). Epidemiology of *Cryptosporidium* infection in cattle in China: a review. Parasite..

[27] Xiao L, Feng Y (2008). Zoonotic cryptosporidiosis. FEMS Immunol Med Microbiol..

[28] Mueller-Doblies D, Giles M, Elwin K, Smith RP, Clifton-Hadley FA, Chalmers RM (2008). Distribution of *Cryptosporidium* species in sheep in the UK. Vet Parasitol..

[29] Quílez J, Torres E, Chalmers RM, Hadfield SJ, del Cacho E, Sánchez-Acedo C (2008). *Cryptosporidium* genotypes and subtypes in lambs and goat kids in Spain. Appl Environ Microbiol..

[30] Robertson LJ (2009). *Giardia* and *Cryptosporidium* infections in sheep and goats: a review of the potential for transmission to humans via environmental contamination. Epidemiol Infect..

[31] Grinberg A, Learmonth J, Kwan E, Pomroy W, Lopez Villalobos N, Gibson I, Widmer G (2008). Genetic diversity and zoonotic potential of *Cryptosporidium parvum* causing foal diarrhea. J Clin Microbiol..

[32] Zintl A, Neville D, Maguire D, Fanning S, Mulcahy G, Smith H (2007). Prevalence of *Cryptosporidium* species in intensively farmed pigs in Ireland. Parasitology..

[33] Wagnerová P, Sak B, McEvoy J, Rost M, Sherwood D, Holcomb K (2016). *Cryptosporidium parvum* and *Enterocytozoon bieneusi* in American mustangs and Chincoteague ponies. Exp Parasitol..

[34] Wells B, Shaw H, Hotchkiss E, Gilray J, Ayton R, Green J (2015). Prevalence, species identification and genotyping *Cryptosporidium* from livestock and deer in a catchment in the Cairngorms with a history of a contaminated public water supply. Parasit Vectors..

[35] García-Presedo I, Pedraza-Díaz S, González-Warleta M, Mezo M, Gómez-Bautista M, Ortega-Mora LM (2013). Presence of *Cryptosporidium scrofarum*, *C. suis* and *C. parvum* subtypes IIaA16G2R1 and IIaA13G1R1 in Eurasian wild boars (*Sus scrofa*). Vet Parasitol..

[36] Moher D, Liberati A, Tetzlaff J, Altman DG (2009). Preferred reporting items for systematic reviews and meta-analyses: the PRISMA statement. PLoS Med..

[37] Huson DH, Bryant D (2006). Application of phylogenetic networks in evolutionary studies. Mol Biol Evol..

[38] DerSimonian R, Laird N (1986). Meta-analysis in clinical trials. Control Clin Trials..

[39] Hribar A. Understanding concentrated animal feeding operations and their impact on communities. 2010. https://www.cdc.gov/nceh/ehs/docs/understanding_cafos_nalboh.pdf.

[40] Statista. Number of pigs worldwide in 2018, by leading country (in million head). 2019. https://www.statista.com/statistics/263964/number-of-pigs-in-selected-countries/.

[41] Zahedi A, Monis P, Aucote S, King B, Paparini A, Jian F (2016). Zoonotic *Cryptosporidium* species in animals inhabiting Sydney water catchments. PLoS ONE..

[42] Jacobson C, Al-Habsi K, Ryan U, Williams A, Anderson F, Yang R (2018). *Cryptosporidium* infection is associated with reduced growth and diarrhoea in goats beyond weaning. Vet Parasitol..

[43] Sweeny JP, Ryan U, Robertson I, Jacobson C (2011). *Cryptosporidium* and *Giardia* associated with reduced lamb carcase productivity. Vet Parasitol..

[44] Thomson S, Hamilton CA, Hope JC, Katzer F, Mabbott NA, Morrison LJ (2017). Bovine cryptosporidiosis: impact, host-parasite interaction and control strategies. Vet Res..

[45] Benschop J, Booker C, Shadbolt T, Weston J (2017). A Retrospective cohort study of an outbreak of cryptosporidiosis among veterinary students. Vet Sci..

[46] Giles M, Chalmers R, Pritchard G, Elwin K, Mueller-Doblies D, Clifton-Hadley F (2009). *Cryptosporidium hominis* in a goat and a sheep in the UK. Vet Rec..

[47] Chalmers RM, Giles M (2010). Zoonotic cryptosporidiosis in the UK–challenges for control. J Appl Microbiol..

[48] Connelly L, Craig B, Jones B, Alexander C (2013). Genetic diversity of *Cryptosporidium* spp. within a remote population of Soay Sheep on St. Kilda Islands, Scotland. Appl Environ Microbiol..

[49] Green S, Higgins J. Cochrane handbook for systematic reviews of interventions. 2006. https://training.cochrane.org/handbook.

[50] Chalmers RM, Katzer F (2013). Looking for *Cryptosporidium*: the application of advances in detection and diagnosis. Trends Parasitol..

[51] Gomez-Couso H, Ortega-Mora LM, Aguado-Martinez A, Rosadio-Alcantara R, Maturrano-Hernandez L, Luna-Espinoza L (2012). Presence and molecular characterisation of *Giardia* and *Cryptosporidium* in alpacas (*Vicugna pacos*) from Peru. Vet Parasitol..

[52] Twomey DF, Barlow AM, Bell S, Chalmers RM, Elwin K, Giles M (2008). Cryptosporidiosis in two alpaca (Lama pacos) holdings in the South-West of England. Vet J..

[53] Wessels J, Wessels M, Featherstone C, Pike R (2013). Cryptosporidiosis in eight-month-old weaned alpacas. Vet Rec..

[54] Alves M, Xiao L, Lemos V, Zhou L, Cama V, da Cunha MB (2005). Occurrence and molecular characterization of *Cryptosporidium* spp. in mammals and reptiles at the Lisbon Zoo. Parasitol Res..

[55] Němejc K, Sak B, Květoňová D, Hanzel V, Jenikova M (2012). The first report on *Cryptosporidium suis* and *Cryptosporidium* pig genotype II in Eurasian wild boars (*Sus scrofa*) (Czech Republic). Vet Parasitol..

[56] Amer S, Zidan S, Feng Y, Adamu H, Li N, Xiao L (2013). Identity and public health potential of *Cryptosporidium* spp. in water buffalo calves in Egypt. Vet Parasitol..

[57] Helmy AY, Krücken J, Nöckler K, Samson-Himmelstjerna G, Zessin KH (2013). Molecular epidemiology of *Cryptosporidium* in livestock animals and humans in the Ismailia province of Egypt. Vet Parasitol..

[58] Mahfouz ME, Mira NN, Amer S (2014). Prevalence and genotyping of *Cryptosporidium* spp. in farm animals in Egypt. J Vet Med Sci..

[59] Ibrahim MA, Abdel-Ghany AE, Abdel-Latef GK, Abdel-Aziz SA, Aboelhadid SM (2016). Epidemiology and public health significance of *Cryptosporidium* isolated from cattle, buffaloes, and humans in Egypt. Parasitol Res..

[60] Abu Samra N, Jori F, Xiao L, Rikhotso O, Thompson PN (2013). Molecular characterization of Cryptosporidium species at the wildlife/livestock interface of the Kruger National Park, South Africa. Comp Immunol Microbiol Infect Dis..

[61] Abeywardena H, Jex AR, von Samson-Himmelstjerna G, Haydon SR, Stevens MA, Gasser RB (2013). First molecular characterisation of *Cryptosporidium* and *Giardia* from *Bubalus bubalis* (water buffalo) in Victoria, Australia. Infect Genet Evol..

[62] Zahedi A, Phasey J, Boland T, Ryan U (2016). First report of *Cryptosporidium* species in farmed and wild buffalo from the Northern Territory, Australia. Parasitol Res..

[63] Caccio SM, Rinaldi L, Cringoli G, Condoleo R, Pozio E (2007). Molecular identification of *Cryptosporidium parvum* and *Giardia duodenalis* in the Italian water buffalo (*Bubalus bubalis*). Vet Parasitol..

[64] Aquino MCC, Widemer G, Zucatto AS, Viol MA, Inacio SV, Nakamura AA (2015). First molecular characterization of *Cryptosporidium* spp. infecting buffalo calves in Brazil. J Eukaryot Microbiol..

[65] Wang R, Zhang L, Ning C, Feng Y, Jian F, Xiao L, Lu B, Ai W, Dong H (2008). Multilocus phylogenetic analysis of *Cryptosporidium andersoni* (Apicomplexa) isolated from a Bactrian camel (*Camelus bactrianus*) in China. Parasitol Res..

[66] Liu X, Zhou X, Zhong Zh, Deng J, Chen W, Cao S (2014). Multilocus genotype and subtype analysis of *Cryptosporidium andersoni* derived from a Bactrian camel (*Camelus bactrianus*) in China. Parasitol Res..

[67] Wegayehu T, Karim R, Anberber M, Adamu H, Erko B, Zhang L, Tilahun G (2016). Prevalence and genetic characterization of *Cryptosporidium* species in dairy calves in Central Ethiopia. PLoS ONE..

[68] Szonyi B, Kangethe EK, Mbae CK, Kakundi EM, Kamwati SK, Mohammed HO (2008). First report of *Cryptosporidium* deer-like genotype in Kenyan cattle. Vet Parasitol..

[69] Kangʼethe EK, Mulinge EK, Skilton RA, Njahira M, Monda JG, Nyongesa C (2012). *Cryptosporidium* species detected in calves and cattle in Dagoretti, Nairobi, Kenya. Trop Anim Health Prod..

[70] Bodager JR, Parsons MB, Wright PC, Rasambainarivo F, Roellig D, Xiao L (2015). Complex epidemiology and zoonotic potential for *Cryptosporidium suis* in rural Madagascar. Vet Parasitol..

[71] Ayinmode AB, Fagbemi BO, Xiao L (2010). Molecular characterization of *Cryptosporidium* spp. in native calves in Nigeria. Parasitol Res..

[72] Maikai BV, Umoh JU, Kwaga JKB, Lawal I, Maikai VA, Camae V, Xiao L (2011). Molecular characterization of *Cryptosporidium* spp. in native breeds of cattle in Kaduna State, Nigeria. Vet Parasitol..

[73] Abu Samra N, Jori F, Cacciò SM, Frean J, Poonsamy B, Thompson PN (2015). *Cryptosporidium* genotypes in children and calves living at the wildlife or livestock interface of the Kruger National Park, South Africa. Onderstepoort J Vet Res..

[74] Soltane R, Guyot K, Dei-Cas E, Ayadi A (2007). Prevalence of *Cryptosporidium* spp. (Eucoccidiorida: Cryptosporiidae) in seven species of farm animals in Tunisia. Parasite..

[75] Geurden T, Goma FY, Siwila J, Phiri IGK, Mwanza AM, Gabriel S (2006). Prevalence and genotyping of *Cryptosporidium* in three cattle husbandry systems in Zambia. Vet Parasitol..

[76] Wang R, Wang H, Sun Y, Zhang L, Jian F, Qi M, Ning C, Xiao L (2011). Characteristics of *Cryptosporidium* transmission in preweaned dairy cattle in Henan, China. J Clin Microbiol..

[77] Wang R, Ma G, Zhao J, Lu Q, Wang H, Zhang L, Jian F, Ning C, Xiao L (2011). *Cryptosporidium andersoni* is the predominant species in post-weaned and adult dairy cattle in China. Parasitol Int..

[78] Huang J, Yue D, Meng Q, Wang R, Zhao J, Li J (2014). Prevalence and molecular characterization of *Cryptosporidium* spp. and *Giardia duodenalis* in dairy cattle in Ningxia, northwestern China. BMC Vet Res..

[79] Khan SM, Debnath C, Pramanik AK, Xiao L, Nozaki T, Ganguly S (2010). Molecular characterization and assessment of zoonotic transmission of *Cryptosporidium* from dairy cattle in West Bengal, India. Vet Parasitol..

[80] Meamar AR, Guyot K, Certad G, Dei-Cas E, Mohraz M, Mohebali M (2007). Molecular characterization of *Cryptosporidium* isolates from humans and animals in Iran. Appl Environ Microbiol..

[81] Fotouhi Ardakani R, Fasihi Harandi M, Soleiman Banaei S, Kamyabi H, Atapour M, Sharifi I (2008). Epidemiology of *Cryptosporidium* infection of cattle in Kerman/Iran and molecular genotyping of some isolates. J Kerman Univ Med Sci..

[82] Pirestani M, Sadraei J, Dalimi A, Zawar M, Vaeznia H (2008). Molecular characterization of *Cryptosporidium* isolates from human and bovine using *18S* rRNA gene in Shahriar county of Tehran, Iran. Parasitol Res..

[83] Tanriverdi S, Markovics A, Arslan MO, Itik A, Shkap V, Widmer G (2006). Emergence of distinct genotypes of *Cryptosporidium parvum* in structured host populations. Appl Environ Microbiol..

[84] Karanis P, Eiji T, Palomino L, Boonrod K, Plutzer J, Ongerth J, Igarashi I (2010). First description of *Cryptosporidium bovis* in Japan and diagnosis and genotyping of *Cryptosporidium* spp. in diarrheic pre-weaned calves in Hokkaido. Vet Parasitol..

[85] Halim NA, Plutzer J, Bakheit MA, Karanis P (2008). First report of *Cryptosporidium* deer-like genotype in Malaysian cattle. Vet Parasitol..

[86] Waldron LS, Dimeski B, Beggs PJ, Ferrari BC, Power ML (2011). Molecular epidemiology, spatiotemporal analysis, and ecology of sporadic human cryptosporidiosis in Australia. App Environ Microbiol..

[87] Nolan MJ, Jex AR, Mansell PD, Browning GF, Gasser RB (2009). Genetic characterization of *Cryptosporidium parvum* from calves by mutation scanning and targeted sequencing-zoonotic implications. Electrophoresis..

[88] Ferguson C. Quantifying Infectious Pathogen Sources in WA Drinking Water Catchments. Report by ASL Water services group. 2010.

[89] Ng J, Yang R, McCarthy S, Gordon C, Hijjawi N, Ryan U (2011). Molecular characterization of *Cryptosporidium* and *Giardia* in pre-weaned calves in Western Australia and New South Wales. Vet Parasitol..

[90] McCarthy S, Ng J, Gordon C, Miller R, Wyber A, Ryan UM (2008). Prevalence of *Cryptosporidium* and *Giardia* species in animals in irrigation catchments in the southwest of Australia. Exp Parasitol..

[91] OʼBrien E, McInnes L, Ryan U (2008). *Cryptosporidium* GP60 genotypes from humans and domesticated animals in Australia, North America and Europe. Exp Parasitol..

[92] Ralston B, Thompson RC, Pethick D, McAllister TA, Olson ME (2010). *Cryptosporidium andersoni* in Western Australian feedlot cattle. Aust Vet J..

[93] Learmonth JJ, Ionas G, Pita AB, Cowie RS (2003). Identification and genetic characterisation of *Giardia* and *Cryptosporidium* strains in humans and dairy cattle in the Waikato Region of New Zealand. Water Sci Technol..

[94] Grinberg A, Lopez-Villalobos N, Pomroy W, Widmer G, Smith H, Tait A (2008). Host-shaped segregation of the *Cryptosporidium parvum* multilocus genotype repertoire. Epidemiol Infect..

[95] Al-Mawly JA, Grinberg A, Velathanthiri N, French N (2015). Cross-sectional study of prevalence, genetic diversity and zoonotic potential of *Cryptosporidium parvum* cycling in New Zealand dairy farms. Parasit Vectors.

[96] Geurden T, Berkvens D, Martens C, Casaert S, Vercruysse J, Claerebout E (2007). Molecular epidemiology with subtype analysis of *Cryptosporidium* in calves in Belgium. Parasitology..

[97] Kvac M, Kouba M, Vitovec J (2006). Age-related and housing-dependence of *Cryptosporidium* infection of calves from dairy and beef herds in South Bohemia. Czech Republic. Vet Parasitol..

[98] Kvac M, Hromadova N, Kvetooova D, Rost M, Sak B (2011). Molecular characterization of *Cryptosporidium* spp. in pre-weaned dairy calves in the Czech Republic: absence of *C. ryanae* and management-associated distribution of *C. andersoni*, *C. bovis* and *C. parvum* subtypes. Vet Parasitol..

[99] Ondrackova Z, Kvac M, Sak B, Kvetonova D, Rost M (2009). Prevalence and molecular characterization of *Cryptosporidium* spp. in dairy cattle in South Bohemia, the Czech Republic. Vet Parasitol..

[100] Langkjaer RB, Vigre H, Enemark HL, Maddox-Hyttel C (2007). Molecular and phylogenetic characterization of *Cryptosporidium* and *Giardia* from pigs and cattle in Denmark. Parasitology..

[101] Enemark HL, Ahrens P, Lowery C, Thamsborg SM, Enemark JMD, Bille-Hansen V, Lind P (2002). *Cryptosporidium andersoni* from a Danish cattle herd: identification and preliminary characterization. Vet Parasitol..

[102] Follet J, Guyot K, Leruste H, Follet-Dumoulin A, Hammouma-Ghelboun O, Certad G, Dei-Cas E, Halama P (2011). *Cryptosporidium* infection in a veal calf cohort in France: molecular characterization of species in a longitudinal study. Vet Res..

[103] Plutzer J, Karanis P (2007). Genotype and subtype analyses of *Cryptosporidium* isolates from cattle in Hungary. Vet Parasitol..

[104] Thompson HP, Dooley JS, Kenny J, McCoy M, Lowery CJ, Moore JE (2007). Genotypes and subtypes of *Cryptosporidium* spp. in neonatal calves in Northern Ireland. Parasitol Res..

[105] Duranti A, Caccio SM, Pozio E, Di Egidio A, De Curtis M, Battisti A, Scaramozzino P (2009). Risk factors associated with *Cryptosporidium parvum* infection in cattle. Zoonoses Public Health..

[106] Rzeżutka A, Kaupke A (2013). Occurrence and molecular identification of *Cryptosporidium* species isolated from cattle in Poland. Vet Parasitol..

[107] Mendonca C, Almeida A, Castro A, Delgado ML, Soares S, Correia da Costa JM, Canada N (2007). Molecular characterization of *Cryptosporidium* and *Giardia* isolates from cattle from Portugal. Vet Parasitol..

[108] Imre K, Lobo ML, Matos O, Popescu C, Genchi C, Dărăbus G (2011). Molecular characterization of *Cryptosporidium* isolates from pre-weaned calves in Romania: is there an actual risk of zoonotic infections?. Vet Parasitol..

[109] Smith HV, Nichols RA, Mallon M, Macleod A, Tait A, Reilly WJ (2005). Natural *Cryptosporidium hominis* infections in Scottish cattle. Vet Rec..

[110] Misic Z, Abe N (2007). Subtype analysis of *Cryptosporidium parvum* isolates from calves on farms around Belgrade, Serbia and Montenegro, using the 60 kDa glycoprotein gene sequences. Parasitology..

[111] Quilez J, Torres E, Chalmers RM, Robinson G, Del Cacho E, Sanchez-Acedo C (2008). *Cryptosporidium* species and subtype analysis from dairy calves in Spain. Parasitology..

[112] Cardona GA, de Lucio A, Bailo B, Cano L, de Fuentes I, Carmena D (2015). Unexpected finding of feline-specific *Giardia duodenalis* assemblage F and *Cryptosporidium felis* in asymptomatic adult cattle in Northern Spain. Vet Parasitol..

[113] Silverlas C, Blanco-Penedo I (2013). *Cryptosporidium* spp. in calves and cows from organic and conventional dairy herds. Epidemiol Infect..

[114] Silverlas C, Näslund K, Björkman C, Mattson JG (2010). Molecular characterisation of *Cryptosporidium* isolates from Swedish dairy cattle in relation to age, diarrhoea and region. Vet Parasitol..

[115] Silverlås C, de Verdier K, Emanuelson U, Mattsson JG, Björkman C (2010). *Cryptosporidium* infection in herds with and without calf diarrhoeal problems. Parasitol Res..

[116] Bjorkman C, Lindstrom L, Oweston C, Ahola H, Troel K, Axen C (2015). *Cryptosporidium* infections in suckler herd beef calves. Parasitology..

[117] Uhde FL, Kaufmann T, Sager H, Albini S, Zanoni R, Schelling E, Meylan M (2008). Prevalence of four enteropathogens in the faeces of young diarrhoeic dairy calves in Switzerland. Vet Rec..

[118] Brook EJ, Hart CA, French NP, Christley RM (2009). Molecular epidemiology of *Cryptosporidium* subtypes in cattle in England. Vet J..

[119] Featherstone CA, Giles M, Marshall JA, Mawhinney IC, Holliman A, Pritchard GC (2010). *Cryptosporidium* species in calves submitted for post-mortem examination in England and Wales. Vet Rec..

[120] Moriarty EM, McEvoy JM, Lowery CJ, Thompson HP, Finn M, Sheridan JJ (2005). Prevalence and characterisation of *Cryptosporidium* species in cattle faeces and on beef carcases at slaughter. Vet Rec..

[121] Smith RP, Chalmers RM, Mueller-Doblies D, Clifton-Hadley FA, Elwin K, Watkins J, Paiba GA, Hadfield SJ, Giles M (2010). Investigation of farms linked to human patients with cryptosporidiosis in England and Wales. Prev Vet Med..

[122] Coklin T, Farber J, Parrington L, Dixon B (2007). Prevalence and molecular characterization of *Giardia duodenalis* and *Cryptosporidium* spp. in dairy cattle in Ontario, Canada. Vet Parasitol..

[123] Coklin T, Uehlinger FD, Farber JM, Barkema HW, O’Handley RM, Dixon B (2009). Prevalence and molecular characterization of *Cryptosporidium* spp. in dairy calves in 11 farms in Prince Edward Island, Canada. Vet Parasitol..

[124] Budu-Amoako E, Greenwood SJ, Dixon BR, Barkema HW, McClure JT (2012). *Giardia* and *Cryptosporidium* on dairy farms and the role these farms may play in contaminating water sources in Prince Edward Island, Canada. J Vet Intern Med..

[125] Budu-Amoako E, Greenwood SJ, Dixon BR, Barkema HW, McClure JT (2012). Occurrence of *Cryptosporidium* and *Giardia* on beef farms and water sources within the vicinity of the farms on Prince Edward Island, Canada. Vet Parasitol..

[126] Santín M, Trout JM, Xiao L, Zhou L, Greiner E, Fayer R (2004). Prevalence and age-related variation of *Cryptosporidium* species and genotypes in dairy calves. Vet Parasitol..

[127] Fayer R, Santın M, Trout JM, Greiner E (2006). Prevalence of species and genotypes of *Cryptosporidium* found in 1-2-year-old dairy cattle in the eastern United States. Vet Parasitol..

[128] Fayer R, Santin M, Trout JM (2007). Prevalence of *Cryptosporidium* species and genotypes in mature dairy cattle on farms in eastern United States compared with younger cattle from the same locations. Vet Parasitol..

[129] Fayer R, Santín M, Dargatz D (2010). Species of *Cryptosporidium* detected in weaned cattle on cow-calf operations in the United States. Vet Parasitol..

[130] Szonyi B, Bordonaro R, Wade SE, Mohammed HO (2010). Seasonal variation in the prevalence and molecular epidemiology of *Cryptosporidium* infection in dairy cattle in the New York City Watershed. Parasitol Res..

[131] Meireles MV, Oliveira FP, Teixeira WF, Coelho WM, Mendes LC (2011). Molecular characterization of *Cryptosporidium* spp. in dairy calves from the state of São Paulo, Brazil. Parasitol Res..

[132] Sevá AP, Funada MR, Souza SO, Nava A, Richtzenhain LJ, Soares RM (2010). Occurrence and molecular characterization of *Cryptosporidium* spp. isolated from domestic animals in a rural area surrounding Atlantic dry forest fragments in Teodoro Sampaio municipality, State of São Paulo, Brazil. Rev Bras Parasitol Vet..

[133] Silva FM, Lopes RS, Araújo-Junior JP (2013). Identification of *Cryptosporidium* species and genotypes in dairy cattle in Brazil. Rev Bras Parasitol Vet..

[134] Kodádková A, Kváč M, Ditrich O, Sak B, Xiao L (2010). *Cryptosporidium muris* in a reticulated giraffe (*Giraffa camelopardalis reticulata*). J Parasitol..

[135] Parsons MB, Travis D, Lonsdorf EV, Lipende I, Roellig DM, Collins A (2015). Epidemiology and molecular characterization of *Cryptosporidium* spp. in humans, wild primates, and domesticated animals in the Greater Gombe Ecosystem, Tanzania. PLoS Negl Trop Dis..

[136] Goma FY, Geurden T, Siwila J, Phiri IGK, Gabriel S, Claerebout E (2007). The prevalence and molecular characterisation of *Cryptosporidium* spp. in small ruminants in Zambia. Small Ruminant Res..

[137] Wang R, Li G, Cui B, Huang J, Cui Z, Zhang S (2014). Prevalence, molecular characterization and zoonotic potential of *Cryptosporidium* spp. in goats in Henan and Chongqing, China. Exp Parasitol..

[138] Koinari M, Lymbery AJ, Ryan UM (2014). *Cryptosporidium* species in sheep and goats from Papua New Guinea. Exp Parasitol..

[139] Geurden T, Thomas P, Casaert S, Vercruysse J, Claerebout E (2008). Prevalence and molecular characterisation of *Cryptosporidium* and *Giardia* in lambs and goat kids in Belgium. Vet Parasitol..

[140] Rieux A, Paraud C, Pors I, Chartier C (2013). Molecular characterization of *Cryptosporidium* spp. in pre-weaned kids in a dairy goat farm in western France. Vet Parasitol..

[141] Paraud C, Pors I, Rieux A, Brunet S (2014). High excretion of *Cryptosporidium ubiquitum* by peri-parturient goats in one flock in western France. Vet Parasitol..

[142] Tzanidakis N, Sotiraki S, Claerebout E, Ehsan A, Voutzourakis N, Kostopoulou D (2014). Occurrence and molecular characterization of *Giardia duodenalis* and *Cryptosporidium* spp. in sheep and goats reared under dairy husbandry systems in Greece. Parasite.

[143] Diaz P, Quilez J, Robinson G, Chalmers RM, Diez-Banos P, Morrondo P (2010). Identification of *Cryptosporidium xiaoi* in diarrhoeic goat kids (*Capra hircus*) in Spain. Vet Parasitol..

[144] Díaz P, Quílez J, Prieto A, Navarro E, Pérez-Creo A, Fernández G (2015). *Cryptosporidium* species and subtype analysis in diarrhoeic pre-weaned lambs and goat kids from north-western Spain. Parasitol Res..

[145] Laatamna AK, Wagnerová P, Sak B, Květoňová D, Aissi M, Rost M (2013). Equine cryptosporidial infection associated with *Cryptosporidium* hedgehog genotype in Algeria. Vet Parasitol..

[146] Liu A, Zhang J, Zhao J, Zhao W, Wang R, Zhang L (2015). The first report of *Cryptosporidium andersoni* in horses with diarrhea and multilocus subtype analysis. Parasit Vectors.

[147] Galuppi R, Piva S, Castagnetti C, Iacono E, Tanel S, Pallaver F (2015). Epidemiological survey on *Cryptosporidium* in an Equine Perinatology Unit. Vet Parasitol..

[148] Chalmers AM, Thomas AL, Butler BA, Davies Morel MCG (2005). Identification of *Cryptosporidium parvum* genotype 2 in domestic horses. Vet Rec..

[149] Burton AJ, Nydam DV, Dearen TK, Mitchell K, Bowman DD, Xiao L (2010). The prevalence of *Cryptosporidium*, and identification of the *Cryptosporidium* horse genotype in foals in New York State. Vet Parasitol..

[150] Kotková M, Němejc K, Sak B, Hanzal V, Květoňová D, Hlásková L (2016). *Cryptosporidium ubiquitum*, *C. muris* and *Cryptosporidium* deer genotype in wild cervids and caprines in the Czech Republic. Folia Parasitol..

[151] Morgan UM, Deplazes P, Forbes DA, Spano F, Hertzberg H, Sargent KD (1999). Sequence and PCR-RFLP analysis of the internal transcribed spacers of the rDNA repeat unit in isolates of *Cryptosporidium* from different hosts. Parasitology..

[152] Johnson J, Buddle R, Reid S, Armson A, Ryan U (2008). Prevalence of *Cryptosporidium* genotypes in pre and post-weaned pigs in Australia. Exp Parasitol..

[153] Ryan UM, Samarasinghe B, Read C, Buddle JR, Robertson ID, Thompson RCA (2003). Identification of a novel *Cryptosporidium* genotype in pigs. Appl Environ Microbiol..

[154] Vitovec J, Hamadejova K, Landova L, Kvac M, Kvetonova D, Sak B (2006). Prevalence and pathogenicity of *Cryptosporidium suis* in pre- and post-weaned pigs. J Vet Med B..

[155] Kváč M, Sak B, Hanzlíková D, Kotilová J, Květoňová D (2009). Molecular characterization of *Cryptosporidium* isolates from pigs at slaughterhouses in South Bohemia, Czech Republic. Parasitol Res..

[156] Kváč M, Hanzlíková D, Sak B, Květoňová D (2009). Prevalence and age-related infection of *Cryptosporidium suis*, *C. muris* and *Cryptosporidium pig* genotype II in pigs on a farm complex in the Czech Republic. Vet Parasitol..

[157] Němejc K, Sak B, Květoňová D, Kernerová N, Rost M, Cama VA, Kváč M (2013). Occurrence of *Cryptosporidium suis* and *Cryptosporidium scrofarum* on commercial swine farms in the Czech Republic and its associations with age and husbandry practices. Parasitol Res..

[158] Petersen HH, Jianmin W, Katakam KK, Mejer H, Thamsborg SM, Anders Dalsgaard A (2015). *Cryptosporidium* and *Giardia* in Danish organic pig farms: seasonal and age-related variation in prevalence, infection intensity and species/genotypes. Vet Parasitol..

[159] Featherstone CA, Marshall JA, Giles M, Sayers AR, Pritchard GC (2010). *Cryptosporidium* species infection in pigs in East Anglia. Vet Rec..

[160] Fiuza VRS, Gallo SSM, Frazao-Teixeira E, Santın M, Fayer R, Oliveira FCR (2011). *Cryptosporidium* pig genotype II diagnosed in Pigs from the State of Rio De Janeiro. Brazil J Parasitol..

[161] García-Presedo I, Pedraza-Díaz S, González-Warleta M, Mezo M, Gómez-Bautista M, Ortega-Mora LM (2013). The first report of *Cryptosporidium bovis*, *C. ryanae* and *Giardia duodenalis* sub-assemblage A-II in roe deer (*Capreolus capreolus*) in Spain. Vet Parasitol..

[162] Wang Y, Feng Y, Cui B, Jian F, Ning C, Wang R, Zhang L, Xiao L (2010). Cervine genotype is the major *Cryptosporidium* genotype in sheep in China. Parasitol Res..

[163] Li P, Cai J, Cai M, Wu W, Li C, Lei M (2016). Distribution of *Cryptosporidium* species in Tibetan sheep and yaks in Qinghai, China. Vet Parasitol..

[164] Yang R, Jacobson C, Gardner G, Carmichael I, Campbell AJD, Ng-Hublin J (2014). Longitudinal prevalence, oocyst shedding and molecular characterisation of *Cryptosporidium* species in sheep across four states in Australia. Vet Parasitol..

[165] Ryan UM, Bath C, Robertson I, Read C, Elliot A, Mcinnes L, Traub R, Besier B (2005). Sheep may not be an important zoonotic reservoir for *Cryptosporidium* and *Giardia* parasites. Appl Environ Microbiol..

[166] Yang R, Jacobson C, Gordon C, Ryan U (2009). Prevalence and molecular characterisation of *Cryptosporidium* and *Giardia* species in pre-weaned sheep in Australia. Vet Parasitol..

[167] Yang R, Gardner GE, Ryan U, Jacobson C (2015). Prevalence and pathogen load of *Cryptosporidium* and *Giardia* in sheep faeces collected from saleyards and in abattoir effluent in Western Australia. Small Ruminant Res..

[168] Imre K, Luca C, Costache M, Sala C, Morar A, Morariu S (2013). Zoonotic *Cryptosporidium parvum* in Romanian newborn lambs (*Ovis aries*). Vet Parasitol.

[169] Díaz P, Quílez J, Chalmers RM, Panadero R, López C, Sánchez-Acedo C (2010). Genotype and subtype analysis of *Cryptosporidium* isolates from calves and lambs in Galicia (NW Spain). Parasitology..

[170] Pritchard GC, Marshall JA, Giles M, Muller-Doblies D, Sayers AR, Marshall RN (2008). *Cryptosporidium* species in lambs submitted for diagnostic postmortem examination in England and Wales. Vet Rec..

[171] Fiuza VR, Cosendey RI, Frazão-Teixeira E, Santín M, Fayer R, Oliveira FC (2011). Molecular characterization of *Cryptosporidium* in Brazilian sheep. Vet Parasitol..

[172] Paz e Silva FM, Lopes RS, Saraiva Bresciani KD, Talamini Amarante AF, Araujo JP (2014). High occurrence of *Cryptosporidium ubiquitum* and *Giardia duodenalis* genotype E in sheep from Brazil. Acta Parasitol..

[173] Zucatto AS, Aquino MCC, Inácio SV, Figueiredo RN, Pierucci JC, Perri SHV (2015). Molecular characterisation of *Cryptosporidium* spp. in lambs in the South-Central region of the State of São Paulo. Arq Bras Med Vet Zootec..

