# Supplementary material for: Cryptosporidium infections in terrestrial ungulates with focus on livestock: a systematic review and meta-analysis
Source: Parasit Vectors. 2019 Sep 14;12:453. doi: 10.1186/s13071-019-3704-4 (PMC6744657; doi:10.1186/s13071-019-3704-4)
Supplement: Supplementary file 2 — Additional file 2: Table S2. Worldwide prevalence of Cryptosporidium spp. in herbivorous animals. [file 13071_2019_3704_MOESM2_ESM.doc]

**Additional file 2: Table S2.** Worldwide prevalence of *Cryptosporidium* spp. in herbivorous animals

| **Host (scientific name)** | **Country** | **Period of study** | **Study**  **type** | **Keeping status** | **Age group(s)** | **Sample size** | | **No. positive (%)** | **Sign^a^** | **Technique(s)** | **Reference Number** |
| --- | --- | --- | --- | --- | --- | --- | --- | --- | --- | --- | --- |
| Family Bovidae |  |  |  |  |  | |  |  |  |  |  |
| Antelope (*Hippotragus equinus*) | USA | n.s. | O | Captive | No age group | | 1 | 00 (00.0) | n.s. | CM | [1] |
| Antelope (*Hippotragus niger*) | USA | n.s. | O | Captive | No age group | | 4 | 2 (50.0) | n.s. | CM | [1] |
| Barbary sheep (*Ammotragus lervia*) | China | 2005 | O | Captive | Adult | | 1 | 1 (100) | n.s. | IFA | [2] |
| Barbary sheep (*Ammotragus lervia*) | Brazil | 2008 | O | Captive | No age group | | 1 | 00 (00.0) | n.s. | CM | [3] |
| Bison (*Bison bison*) | Canada | 1998-2000 | P | Ranched | No age group | | 41 | 2 (4.9) | n.s. | IFA | [4] |
| Bison (*Bison bison*) | Belgium | n.s. | P | Farmed | < 6 months old | | 82 | 3 (3.7) | n.s. | IFA | [5] |
| Bison (*Bison bonasus*) | USA | n.s. | O | Captive | No age group | | 2 | 00 (00.0) | n.s. | CM | [1] |
| Bison (*Bison bonasus*) | Poland | 2002-2003 | P | Wild | No age group | | 55 | 16 (29.1) | n.s. | CM | [6] |
| Blackbuck (*Antilope cervicapra*) | USA | n.s. | O | Captive | No age group | | 24 | 4 (16.7) | n.s. | CM | [1] |
| Bongo (*Tragelaphus eurycerus*) | USA | n.s. | O | Captive | No age group | | 1 | 00 (00.0) | n.s. | CM | [1] |
| Buffalo (*Bubalus bubalis*) | India | 1990 | P | n.s. | 7-80 days old | | 60 | 10 (16.6) | n.s. | CM | [7] |
| Buffalo (*Bubalus bubalis*) | India | 1992 | P | n.s. | < 1 months old | | 18 | 1 (5.5) | n.s. | CM | [7] |
| Buffalo (*Bubalus bubalis*) | Brazil | 1996 | P | Farmed | 3-45 days old | | 106 | 10 (9.4) | M | CM | [8] |
| Buffalo (*Bubalus bubalis*) | India | n.s. | P | Farmed | 5 age groups | | 64 | 23 (35.9) | M | CM | [9] |
| Buffalo (*Bubalus bubalis*) | Iran | 2002-2003 | P | Slaughtered | No age group | | 300 | 11 (3.6) | n.s. | CM | [10] |
| Buffalo (*Bubalus bubalis*) | Italy | 2006 | P | Farmed | 1-9 weeks old | | 346 | 51 (14.7) | A | ELISA | [11] |
| Buffalo (*Bubalus bubalis*) | Italy | n.s. | O | Farmed | No age group | | 57 | 8 (14.0) | A | IFA | [12] |
| Buffalo (*Bubalus bubalis*) | Egypt | 2005-2006 | P | Farmed | 1-15 days old | | 458 | 65 (14.2) | M | CM | [13] |
| Buffalo (*Bubalus bubalis*) | Pakistan | n.s. | P | Farmed | 4 age groups | | 250 | 60 (24.0) | M | CM | [14] |
| Buffalo (*Bubalus bubalis*) | India | 2009-2010 | P | Farmed | < 5 months old | | 162 | 62 (38.3) | M | CM | [15] |
| Buffalo (*Bubalus bubalis*) | Iran | 2009-2011 | O | Farmed | Adults | | 8 | 4 (50.0) | n.s. | CM | [16] |
| Buffalo (*Bubalus bubalis*) | Nepal | 2010 | P | Farmed | 2-7 months old | | 81 | 30 (37.0) | n.s. | CM | [17] |
| Buffalo (*Bubalus bubalis*) | Australia | 2002 | P | Farmed | 3 age groups | | 476 | 62 (13.1) | n.s. | PCR | [18] |
| Buffalo (*Bubalus bubalis*) | Pakistan | 2007-2008 | P | Farmed | No age group | | 720 | 61 (8.4) | n.s. | CM | [19] |
| Buffalo (*Bubalus bubalis*) | India | 2009-2012 | P | Farmed | < 3 months old | | 264 | 64 (24.2) | M | CM | [20] |
| Buffalo (*Bubalus bubalis*) | India | n.s. | P | Farmed | 1-2 months old | | 34 | 8 (23.5) | S | ICT | [21] |
| Buffalo (*Bubalus bubalis*) | Egypt | 2010-2011 | P | Farmed | 2 age groups | | 538 | 17 (3.1) | A | CM | [22] |
| Buffalo (*Bubalus bubalis*) | Egypt | 2011 | P | Farmed | 4 age groups | | 211 | 75 (35.5) | M | PCR | [23] |
| Buffalo (*Bubalus bubalis*) | Egypt | 2010-2011 | P | Farmed | 3 age groups | | 466 | 6 (1.29) | n.s. | CM | [24] |
| Buffalo (*Bubalus bubalis*) | Egypt | n.s. | P | Farmed | 4 age groups | | 571 | 297 (52.0) | M | CM | [25] |
| Buffalo (*Bubalus bubalis*) | Thailand | 2010-2011 | P | Farmed | 4 age groups | | 600 | 34 (5.7) | n.s. | CM | [26] |
| Buffalo (*Bubalus bubalis*) | Iran | 2011-2012 | P | Farmed | < 1 years old | | 90 | 41 (45.5) | M | CM | [27] |
| Buffalo (*Bubalus bubalis*) | Sri Lanka | 2012-2013 | P | Farmed | 2 age groups | | 297 | 29 (9.8) | n.s. | PCR | [28] |
| Buffalo (*Bubalus bubalis*) | Brazil | 2010 | P | Farmed | 4 age groups | | 220 | 107 (48.2) | n.s. | PCR | [29] |
| Buffalo (*Bubalus bubalis*) | Iraq | 2013-2014 | P | Farmed | 2 age groups | | 130 | 13 (10.0) | M | CM | [30] |
| Buffalo (*Bubalus bubalis*) | India | 2012-2013 | P | Farmed | 2 age groups | | 246 | 91 (37.0) | M | CM | [31] |
| Buffalo (*Bubalus bubalis*) | Egypt | 2014 | P | Farmed | 3 age groups | | 130 | 16 (12.3) | M | CM | [32] |
| Buffalo (*Bubalus bubalis*) | Australia | 2015 | P | Farmed | Adults | | 50 | 15 (30.0) | n.s. | PCR | [33] |
| Buffalo (*Bubalus bubalis*) | Australia | 2015 | P | Wild | Adults | | 50 | 6 (12.0) | n.s. | PCR | [33] |

**Additional file 2: Table S2.** (*Continuted*)

| **Host (Scientific name)** | **Country** | **Period of study** | **Study**  **type** | **Keeping status** | **Age group(s)** | **Sample size** | **No. positive (%)** | **Sign^a^** | **Technique(s)** | **Reference Number** |
| --- | --- | --- | --- | --- | --- | --- | --- | --- | --- | --- |
| Buffalo (*Syncerus caffer*) | USA | n.s. | O | Captive | No age group | 1 | 00 (00.0) | n.s. | CM | [1] |
| Buffalo (*Syncerus caffer*) | Spain | n.s. | O | Captive | No age group | 3 | 1 (33.3) | n.s. | CM | [34] |
| Buffalo (*Syncerus caffer*) | Tanzania | 1995 | P | Wild | No age group | 36 | 8 (22.0) | n.s. | CM | [35] |
| Buffalo (*Syncerus caffer*) | South Africa | 2008 | P | Wild | No age group | 91 | 5 (5.5) | n.s. | CM | [36] |
| Buffalo (*Syncerus caffer*) | Rwanda | 2010 | P | Wild | 2 age groups | 55 | 20 (36.0) | n.s. | IFA | [37] |
| Cattle (*Bos taurus*) | Bangladesh | 1982-1983 | P | Farmed | 1-28 days old | 510 | 31 (6.0) | M | CM | [38] |
| Cattle (*Bos indicus*) | Netherland | n.s. | P^b^ | Farmed | Calves | 45 | 27 (60.0) | n.s. | CM | [39] |
| Cattle (*Bos taurus*) | Argentina | n.s. | P | Farmed | < 1 month old | 452 | 136 (30.0) | S | CM | [40] |
| Cattle (*Bos taurus*) | South Korea | n.s. | P | Farmed | 2-5 years old | 500 | 111 (22.2) | n.s. | CM | [41] |
| Cattle (*Bos taurus*) | Spain | 1987-1989 | P | Farmed | 2 age groups | 141 | 9 (6.4) | M | CM | [42] |
| Cattle (*Bos taurus*) | India | 1990 | P | n.s. | 7-80 days old | 45 | 8 (17.7) | n.s. | CM | [7] |
| Cattle (*Bos taurus*) | India | 1992 | P | n.s. | < 1 months old | 48 | 4 (8.3) | n.s. | CM | [7] |
| Cattle (*Bos taurus*) | Spain | 1990 | P | Farmed | Adults | 131 | 94 (71.7) | A | CM | [43] |
| Cattle (*Bos taurus*) | Trinidad | n.s. | P | Farmed | < 24 days old | 298 | 26 (8.7) | M | CM | [44] |
| Cattle (*Bos taurus*) | Trinidad | 1992 | P | Farmed | ≤ 6 months old | 298 | 26 (8.7) | M | CM | [45] |
| Cattle (*Bos taurus*) | USA | n.s. | P | Farmed | Pre-weaned | 7,369 | 1,648 (22.4) | n.s. | IFA | [46] |
| Cattle (*Bos taurus*) | UK | 1991 | P | Farmed | Adults | 553 | 345 (62.4) | A | CM | [47] |
| Cattle (*Bos taurus*) | Scotland | n.s. | P | Farmed | Adults | 109 | 25 (23.0) | n.s. | IFA | [48] |
| Cattle (*Bos taurus*) | South Korea | n.s. | O | n.s. | < 4 months old | 201 | 29 (14.4) | S | CM | [49] |
| Cattle (*Bos taurus*) | Spain | 1990-1993 | P | Farmed | 3 age groups | 554 | 109 (19.7) | M | CM | [50] |
| Cattle (*Bos taurus*) | USA | 1992-1994 | O^d^ | n.s. | No age group | 214 | 39 (18.2) | n.s. | CM | [51] |
| Cattle (*Bos taurus*) | Brazil | 1994-1995 | P^b^ | Farmed | < 20 months old | 30 | 14 (46.7) | A | CM | [52] |
| Cattle (*Bos taurus*) | Canada | 1995 | P | Farmed | 2 age groups | 104 | 21 (20.0) | A | IFA | [53] |
| Cattle (*Bos taurus*) | Tanzania | 1995 | P | Farmed | 3 age groups | 486 | 26 (5.3) | M | CM | [35] |
| Cattle (*Bos taurus*) | Costa Rica | 1993-1994 | P | Farmed | < 3 months old | 187 | 21 (11.0) | S | CM | [54] |
| Cattle (*Bos taurus*) | Mexico | 1996-1997 | P | Farmed | 1-30 days old | 512 | 128 (25.0) | n.s. | CM | [55] |
| Cattle (*Bos taurus*) | Poland | 1997 | P | Farmed | 3-16 days old | 75 | 38 (51.0) | n.s. | CM | [56] |
| Cattle (*Bos taurus*) | Turkey | n.s. | P | Farmed | 4 age groups | 302 | 199 (65.8) | M | CM | [57] |
| Cattle (*Bos taurus*) | Spain | 1993-1995 | P | Farmed | 1-30 days old | 218 | 114 (52.3) | S | CM | [58] |
| Cattle (*Bos taurus*) | France | 1996-1997 | O | Farmed | 3-30 days old | 301 | 78 (25.9) | n.s. | QLAT | [59] |
| Cattle (*Bos taurus*) | USA | 1993-1994 | P | Farmed | 3 age groups | 2,943 | 58 (1.9) | M | CM | [60] |
| Cattle (*Bos taurus*) | USA | 1997 | P | Farmed | No age group | 557 | 6 (1.1) | n.s. | IFA | [61] |
| Cattle (*Bos taurus*) | Japan | 1998-1999 | P^b^ | Farmed | ≤ 30 days old | 30 | 28 (93.0) | M | CM | [62] |
| Cattle (*Bos taurus*) | Portugal | 1996-1998 | P | Farmed | 12 age groups | 553 | 129 (23.3) | n.s. | ELISA | [63] |
| Cattle (*Bos taurus*) | Netherland | 1999-2000 | P | Farmed | 4 age groups | 66 | 33 (50.0) | M | IFA | [64] |
| Cattle (*Bos taurus*) | Serbia | n.s. | P | Farmed | < 3 months old | 126 | 68 (53.9) | M | CM | [65] |
| Cattle (*Bos taurus*) | Denmark | 1997 | P | Farmed | 3 age groups | 142 | 33 (23.2) | n.s. | CM | [66] |
| Cattle (*Bos taurus*) | Denmark | 1998 | P | Farmed | 3 age groups | 150 | 67 (44.6) | n.s. | CM | [66] |
| Cattle (*Bos taurus*) | Poland | 1999-2001 | P | Farmed | Calves | 171 | 42 (24.5) | n.s. | ELISA | [67] |
| Cattle (*Bos taurus*) | Iraq | n.s. | P | n.s. | No age group | 60 | 12 (20.0) | n.s. | CM | [68] |
| Cattle (*Bos taurus*) | Serbia | n.s. | P | Farmed | 6 age groups | 160 | 72 (45.0) | M | CM | [69] |
| Cattle (*Bos taurus*) | UK | n.s. | P | Farmed | 2 age groups | 1,495 | 173 (11.5) | n.s. | CM | [70] |

**Additional file 2: Table S2.** (*Continuted*)

| **Host (Scientific name)** | **Country** | **Period of study** | **Study**  **type** | **Keeping status** | **Age group(s)** | **Sample size** | | **No. positive (%)** | **Sign^a^** | **Technique(s)** | **Reference Number** |
| --- | --- | --- | --- | --- | --- | --- | --- | --- | --- | --- | --- |
| Cattle (*Bos taurus*) | Sweden | 1998-1999 | O | Farmed | ≤ 3 months old | | 270 | 22 (8.1) | M | CM | [71] |
| Cattle (*Bos taurus*) | Ireland | 2002 | p | Slaughtered | No age group | | 288 | 21 (7.3) | n.s. | IFA | [72] |
| Cattle (*Bos taurus*) | New Zealand | n.s. | p | Farmed | 2 age groups | | 658 | 35 (5.3) | n.s. | n.s. | [73] |
| Cattle (*Bos taurus*) | USA | n.s. | P | Herded | Adults | | 240 | 17 (7.1) | n.s. | IFA | [74] |
| Cattle (*Bos taurus*) | Australia | 2000 | P^b^ | Farmed | < 12 weeks old | | 54 | 26 (48.1) | n.s. | CM | [75] |
| Cattle (*Bos taurus*) | Germany | 1998-2002 | P | n.s. | No age group | | 998 | 35 (3.5) | n.s. | n.s. | [76] |
| Cattle (*Bos taurus*) | South Korea | 2001-2002 | P | Farmed | No age group | | 947 | 258 (27.2) | n.s. | CM | [77] |
| Cattle (*Bos taurus*) | USA | 2002-2003 | P | Farmed | 2 age groups | | 971 | 345 (35.5) | A | CM | [78] |
| Cattle (*Bos taurus*) | Canada | 1998 | P | Ranched | < 6 weeks old | | 193 | 25 (13.0) | n.s. | IFA | [79] |
| Cattle (*Bos taurus*) | Canada | 1998-1999 | P | Farmed | Adults | | 669 | 194 (29.0) | A | IFA | [79] |
| Cattle (*Bos taurus*) | Japan | 2001 | P | Slaughtered | Adults | | 325 | 5 (1.5) | n.s. | CM | [80] |
| Cattle (*Bos taurus*) | Canada | 2002 | P | Farmed | 7-21 days old | | 500 | 203 (40.6) | M | CM | [81] |
| Cattle (*Bos taurus*) | Malaysia | n.s. | P | n.s. | No age group | | 96 | 24 (25.0) | n.s. | IFA | [82] |
| Cattle (*Bos taurus*) | Taiwan | n.s. | P | Farmed | 5 age groups | | 460 | 173 (37.6) | M | CM | [83] |
| Cattle (*Bos taurus*) | New Zealand | 2002 | P | Farmed | Newborn calves | | 156 | 33 (21.2) | n.s. | CM | [84] |
| Cattle (*Bos taurus*) | Turkey | 2002 | P | Farmed | 6 age groups | | 457 | 294 (64.3) | n.s. | CM | [85] |
| Cattle (*Bos taurus*) | Tanzania | 2003-2004 | P | Farmed | No age group | | 942 | 5 (0.5) | n.s. | CM | [86] |
| Cattle (*Bos taurus*) | UK | 2005 | P | Slaughtered | 1-2 years old | | 288 | 21 (7.3) | n.s. | IFA | [87] |
| Cattle (*Bos taurus*) | Argentina | n.s. | P | Farmed | Calves | | 109 | 29 (26.6) | n.s. | CM | [88] |
| Cattle (*Bos taurus*) | Turkey | 2000-2005 | P^d^ | n.s. | < 8 months old | | 231 | 43 (18.6) | S | CM | [89] |
| Cattle (*Bos taurus*) | Iran | 2001 | P | Farmed | 3 age groups | | 291 | 63 (21.7) | M | CM | [90] |
| Cattle (*Bos taurus*) | Canada | 2002 | P | Farmed | All age groups | | 1,165 | 24 (2.0) | M | IFA | [91] |
| Cattle (*Bos taurus*) | Czechia | 2002-2005 | P | Farmed | 2 age groups | | 4,273 | 1,305 (30.5) | n.s. | CM | [92] |
| Cattle (*Bos taurus*) | Czechia | 2002-2005 | P | Farmed | 2 age groups | | 2,748 | 509 (18.5) | n.s. | CM | [92] |
| Cattle (*Bos taurus*) | Denmark | 2003-2004 | P | Farmed | 3 age groups | | 1,150 | 366 (31.8) | M | IFA | [93] |
| Cattle (*Bos taurus*) | India | 2003-2004 | P | n.s. | < 1 year old | | 470 | 82 (17.4) | M | CM | [94] |
| Cattle (*Bos taurus*) | India | n.s. | P | Farmed | 5 age groups | | 90 | 36 (40.0) | M | CM | [9] |
| Cattle (*Bos taurus*) | USA | 2004 | P | Farmed | 1-2 years old | | 571 | 68 (11.9) | n.s. | PCR | [95] |
| Cattle (*Bos taurus*) | Austria | 2004-2005 | P | Farmed | 1-42 days old | | 230 | 27 (11.7) | M | CM | [96] |
| Cattle (*Bos taurus*) | India | 2004-2005 | P | n.s. | < 1 year old | | 470 | 85 (18.0) | M | CM | [94] |
| Cattle (*Bos taurus*) | Norway | n.s. | P | Farmed | < 6 months old | | 1,386 | 167 (12.0) | n.s. | IFA | [97] |
| Cattle (*Bos taurus*) | Spain | 2005 | P | Farmed | 12 age groups | | 734 | 104 (14.2) | A | IFA | [98] |
| Cattle (*Bos taurus*) | Zambia | n.s. | P | Farmed | < 3 months old | | 744 | 142 (19.2) | M | ELISA | [99] |
| Cattle (*Bos taurus*) | Tanzania | 1999 | P | Farmed | 3 age groups | | 1,129 | 222 (19.7) | n.s. | CM | [100] |
| Cattle (*Bos taurus*) | Tunisia | 2000 | P^b^ | Farmed | < 30 days old | | 30 | 26 (86.7) | n.s. | CM | [101] |
| Cattle (*Bos taurus*) | Belgium | 2001-2005 | P | Farmed | < 10 weeks old | | 832 | 224 (26.9) | M | IFA | [102] |
| Cattle (*Bos taurus*) | Ireland | 2003-2003 | P | n.s. | < 1 months old | | 779 | 291 (37.4) | S | CM | [103] |
| Cattle (*Bos taurus*) | Serbia | 2003 | P | Farmed | < 30 days old | | 103 | 62 (60.2) | n.s. | PCR | [104] |
| Cattle (*Bos taurus*) | Canada | 2003-2004 | P^b^ | Farmed | < 30 days old | | 919 | 716 (78.0) | M | CM | [105] |
| Cattle (*Bos taurus*) | Iran | 2003-2004 | P | n.s. | 3 age groups | | 480 | 30 (6.25) | M | CM | [106] |
| Cattle (*Bos taurus*) | Portugal | 2004-2006 | P | Farmed | 2 age groups | | 467 | 82 (17.5) | n.s. | CM | [107] |

**Additional file 2: Table S2.** (*Continuted*)

| **Host (Scientific name)** | **Country** | **Period of study** | | **Study**  **type** | | **Keeping status** | **Age group(s)** | **Sample size** | **No. positive (%)** | **Sign^a^** | **Technique(s)** | | **Reference Number** |
| --- | --- | --- | --- | --- | --- | --- | --- | --- | --- | --- | --- | --- | --- |
| Cattle (*Bos taurus*) | Canada | | 2005 | | P | Farmed | 3 age groups | 143 | 39 (27.3) | n.s. | PCR | [108] | |
| Cattle (*Bos taurus*) | USA | | 2005 | | P | Farmed | > 2 years old | 541 | 31 (5.7) | n.s. | PCR | [109] | |
| Cattle (*Bos taurus*) | Spain | | n.s. | | P | Farmed | 3-13 years old | 379 | 32 (8.4) | A | IFA | [110] | |
| Cattle (*Bos taurus*) | Hungary | | 2006 | | P | Farmed | Pre-weaned | 79 | 39 (49.3) | S | IFA | [111] | |
| Cattle (*Bos taurus*) | Iran | | n.s. | | P | n.s. | < 7 months old | 292 | 35 (11.9) | n.s. | CM | [112] | |
| Cattle (*Bos taurus*) | Iran | | 2003-2004 | | P | n.s. | 3 age groups | 412 | 78 (18.9) | M | CM | [113] | |
| Cattle (*Bos taurus*) | UK | | 2004 | | P | Farmed | 26 days old | 215 | 60 (28.0) | n.s. | PCR | [114] | |
| Cattle (*Bos taurus*) | USA | | 2004 | | P | Farmed | 7-28 days old | 1,089 | 324 (29.7) | n.s. | CM | [115] | |
| Cattle (*Bos taurus*) | Switzerland | | 2005-2006 | | P | Farmed | 1-21 days old | 147 | 81 (55.1) | S | CM | [116] | |
| Cattle (*Bos taurus*) | Turkey | | 2005-2006 | | P | n.s. | < 6 months old | 182 | 24 (13.1) | n.s. | CM | [117] | |
| Cattle (*Bos taurus*) | Kenya | | 2006 | | P | Farmed | 7-30 days old | 143 | 34 (23.8) | n.s. | CM | [118] | |
| Cattle (*Bos taurus*) | Turkey | | 2006 | | P | Slaughtered | No age group | 86 | 7 (8.1) | n.s. | ICT | [119] | |
| Cattle (*Bos taurus*) | India | | 2006-2007 | | P | Farmed | < 3 months old | 457 | 138 (30.2) | M | PCR | [120] | |
| Cattle (*Bos taurus*) | Mongolia | | 2006-2007 | | P | Farmed | 2 age groups | 439 | 116 (26.4) | n.s. | IFA | [121] | |
| Cattle (*Bos taurus*) | Spain | | 2006-2007 | | P | Farmed | < 21 days old | 287 | 166 (57.8) | S | CM | [122] | |
| Cattle (*Bos taurus*) | Thailand | | 2007 | | P | Farmed | < 4 months old | 200 | 26 (13.0) | n.s. | CM | [123] | |
| Cattle (*Bos taurus*) | Turkey | | 2007 | | P | Farmed | ≤ 3 months old | 189 | 43 (22.8) | M | CM | [124] | |
| Cattle (*Bos taurus*) | Argentina | | 2007 | | P | Farmed | ≤ 1 month old | 280 | 48 (17.1) | M | CM | [125] | |
| Cattle (*Bos taurus*) | Malaysia | | 2007 | | P | Farmed | 1-6 months old | 50 | 18 (36.0) | M | CM | [126] | |
| Cattle (*Bos taurus*) | New Zealand | | n.s. | | P | Farmed | No age group | 155 | 8 (5.2) | n.s. | IFA | [127] | |
| Cattle (*Bos taurus*) | USA | | n.s. | | P | Farmed | 4 age groups | 212 | 43 (20.3) | A | PCR | [128] | |
| Cattle (*Bos taurus*) | New Zealand | | 2005-2006 | | p | Farmed | 1-7 weeks old | 1,190 | 31 (2.6) | n.s. | IFA | [129] | |
| Cattle (*Bos taurus*) | Italy | | 2001-2003 | | P | Farmed | 7 age groups | 2,024 | 101 (5.0) | n.s. | IFA | [130] | |
| Cattle (*Bos taurus*) | Italy | | 2001-2003 | | P | Farmed | 7 age groups | 2,024 | 155 (8.0) | n.s. | ELISA | [130] | |
| Cattle (*Bos taurus*) | Malavi | | 2001-2003 | | P | Farmed | 2 age groups | 825 | 142 (17.2) | M | CM | [131] | |
| Cattle (*Bos taurus*) | Australia | | 2005 | | P | Farmed | 1-30 days age | 268 | 124 (46.3) | n.s. | PCR | [132] | |
| Cattle (*Bos taurus*) | Sweden | | 2005-2007 | | P | Farmed | 3 age groups | 1,202 | 403 (33.5) | A | IFA | [133] | |
| Cattle (*Bos taurus*) | USA | | 2005-2008 | | O | n.s. | 2 age groups | 19 | 1 (5.2) | n.s. | PCR | [134] | |
| Cattle (*Bos taurus*) | Canada | | 2003-2004 | | P | Farmed | < 6 months old | 183 | 10 (6.2) | n.s. | IFA | [135] | |
| Cattle (*Bos taurus*) | Austria | | n.s. | | O | Farmed | 1-42 days old | 180 | 58 (32.2) | n.s. | ICT | [136] | |
| Cattle (*Bos taurus*) | Iran | | 2006-2007 | | P | Farmed | 1-20 weeks old | 272 | 51 (18.75) | n.s. | CM | [137] | |
| Cattle (*Bos taurus*) | India | | 2007 | | P | Farmed | 3 age groups | 350 | 45 (12.8) | n.s. | PCR | [138] | |
| Cattle (*Bos taurus*) | India | | 2007 | | P | Farmed | 4 age groups | 455 | 26 (5.7) | M | CM | [139] | |
| Cattle (*Bos taurus*) | Japan | | 2007 | | P | Farmed | > 24 months old | 50 | 5 (10.0) | n.s. | PCR | [140] | |
| Cattle (*Bos taurus*) | Spain | | 2007 | | P | Farmed | 3 age groups | 1,316 | 85 (6.5) | M | IFA | [141] | |
| Cattle (*Bos taurus*) | Czechia | | 2007-2008 | | P | Slaughtered | 3 age groups | 995 | 49 (4.9) | n.s. | CM | [142] | |
| Cattle (*Bos taurus*) | China | | 2008 | | P | Farmed | 4 age groups | 507 | 27 (5.3) | n.s. | CM | [143] | |
| Cattle (*Bos taurus*) | Pakistan | | n.s. | | P | Farmed | 4 age groups | 250 | 68 (27.2) | M | CM | [14] | |
| Cattle (*Bos taurus*) | Poland | | n.s. | | P | Imported | Heifers | 75 | 23 (30.7) | n.s. | CM | [144] | |
| Cattle (*Bos taurus*) | Poland | | n.s. | | P | Imported | Heifers | 105 | 12 (11.3) | n.s. | CM | [144] | |
| Cattle (*Bos taurus*) | Poland | | n.s. | | P | Farmed | Adults | 60 | 00 (00.0) | n.s. | CM | [145] | |
| Cattle (*Bos taurus*) | Australia | | n.s. | | P | Farmed | 10 age groups | 567 | 20 (3.6) | A | IFA | [146] | |
| Cattle (*Bos taurus*) | Tanzania | | 2003-2004 | | P | Farmed | *≤* 3 months old | 117 | 41 (35.0) | n.s. | CM | [147] | |

**Additional file 2: Table S2.** (*Continuted*)

| **Host (Scientific name)** | **Country** | **Period of study** | **Study**  **type** | **Keeping status** | **Age group(s)** | **Sample size** | **No. positive (%)** | **Sign^a^** | **Technique(s)** | **Reference Number** |
| --- | --- | --- | --- | --- | --- | --- | --- | --- | --- | --- |
| Cattle (*Bos taurus*) | England & Wales | 2004-2006 | P | Farmed | 3 age groups | 116 | 62 (53.4) | n.s. | IFA | [148] |
| Cattle (*Bos taurus*) | Sweden | 2006-2008 | P^e^ | Farmed | 3 age groups | 483 | 217 (44.9) | M | IFA | [149] |
| Cattle (*Bos taurus*) | Brazil | 2007 | P | Farmed | No age group | 197 | 6 (3.0) | n.s. | CM | [150] |
| Cattle (*Bos taurus*) | Netherland | 2007 | P | Farmed | 1-21 days old | 424 | 118 (27.8) | M | ICT | [151] |
| Cattle (*Bos taurus*) | Japan | 2007-2008 | P | n.s. | 3-45 days old | 80 | 60 (75.0) | S | PCR | [152] |
| Cattle (*Bos taurus*) | USA | 2007-2008 | P | Farmed | 6-8 month old | 819 | 164 (20.0) | n.s. | PCR | [153] |
| Cattle (*Bos taurus*) | England & Wales | 2008 | P^d^ | Farmed | < 3 months old | 229 | 103 (45.0) | M | IFA | [154] |
| Cattle (*Bos taurus*) | India | 2008-2009 | P | Farmed | 3 age groups | 180 | 21 (11.7) | n.s. | PCR | [155] |
| Cattle (*Bos taurus*) | Spain | 2008-2009 | P | Farmed | Pre-weaned | 61 | 30 (49.2) | S | CM | [156] |
| Cattle (*Bos taurus*) | Nigeria | 2009 | P | Farmed | 12-24 week old | 65 | 34 (52.3) | S | PCR | [157] |
| Cattle (*Bos taurus*) | Nigeria | n.s. | P | n.s. | 3 age groups | 406 | 95 (23.4) | M | CM | [158] |
| Cattle (*Bos taurus*) | Thailand | n.s. | P | Farmed | 4 age groups | 200 | 31 (15.5) | n.s. | PCR | [159] |
| Cattle (*Bos taurus*) | USA | n.s. | P | Farmed | 3 age groups | 1,911 | 115 (6.0) | n.s. | CM | [160] |
| Cattle (*Bos taurus*) | Australia | 2006-2008 | P | Farmed | < 4 months old | 364 | 82 (22.5) | n.s. | PCR | [161] |
| Cattle (*Bos taurus*) | China | 2006-2009 | P | Farmed | 3 age groups | 1,315 | 104 (7.9) | A | CM | [162] |
| Cattle (*Bos taurus*) | France | 2007 | P^b^ | Farmed | 5 weeks old | 142 | 100 (70.4) | S | PCR | [163] |
| Cattle (*Bos taurus*) | Australia | 2007-2008 | P^d^ | Farmed | < 42 days old | 597 | 349 (58.5) | S | ELISA | [164] |
| Cattle (*Bos taurus*) | Brazil | 2007-2008 | P | Farmed | < 30 days old | 196 | 21 (10.7) | n.s. | PCR | [165] |
| Cattle (*Bos taurus*) | Chile | 2007-2008 | P | Farmed | < 4 weeks old | 205 | 115 (56.1) | S | CM | [166] |
| Cattle (*Bos taurus*) | Argentina | 2008 | P | Farmed | < 7 weeks old | 620 | 119 (19.3) | n.s. | CM | [167] |
| Cattle (*Bos taurus*) | Iran | 2008 | P | Farmed | < 1 months old | 224 | 82 (36.6) | M | CM | [168] |
| Cattle (*Bos taurus*) | China | 2008-2009 | P | Farmed | Pre weaned | 801 | 172 (21.5) | n.s. | CM | [169] |
| Cattle (*Bos taurus*) | Czechia | 2008-2009 | P | Farmed | < 2 months old | 750 | 161 (21.4) | M | CM | [170] |
| Cattle (*Bos taurus*) | Malaysia | 2008-2009 | P | Farmed | ≤ 12 months old | 240 | 65 (27.1) | n.s. | PCR | [171] |
| Cattle (*Bos taurus*) | Nigeria | 2008-2009 | P | Farmed | 2-365 days old | 194 | 31 (16.0) | n.s. | PCR | [172] |
| Cattle (*Bos taurus*) | Spain | 2008-2009 | P | Farmed | 2 age groups | 227 | 7 (3.0) | A | ELISA | [173] |
| Cattle (*Bos taurus*) | Australia | 2008-2010 | P | Farmed | No age group | 205 | 62 (30.0) | n.s. | PCR | [174] |
| Cattle (*Bos taurus*) | Romania | 2008-2011 | P | Farmed | 1-30 days old | 258 | 65 (25.5) | n.s. | CM | [175] |
| Cattle (*Bos taurus*) | Mexico | 2010 | P | Farmed | 2 age groups | 272 | 97 (35.7) | n.s. | CM | [176] |
| Cattle (*Bos taurus*) | Canada | 2006 | P | Farmed | 3 age groups | 752 | 108 (14.0) | n.s. | IFA | [177] |
| Cattle (*Bos taurus*) | Canada | 2006 | P | Farmed | 2 age groups | 739 | 123 (17.0) | n.s. | IFA | [178] |
| Cattle (*Bos taurus*) | Kenya | 2006 | P^f^ | Farmed | No age group | 872 | 131 (15.0) | n.s. | CM | [179] |
| Cattle (*Bos taurus*) | Kenya | 2006 | P^g^ | Farmed | No age group | 862 | 95 (11.0) | n.s. | CM | [179] |
| Cattle (*Bos taurus*) | China | 2007-2009 | P | Farmed | < 5 years old | 2,056 | 387 (18.8) | n.s. | CM | [180] |
| Cattle (*Bos taurus*) | USA | 2007-2010 | P | Pastured | No age group | 201 | 13 (6.5) | n.s. | PCR | [181] |
| Cattle (*Bos taurus*) | Iran | 2009-2011 | P | n.s. | 2 age groups | 80 | 51 (63.7) | n.s. | CM | [16] |
| Cattle (*Bos taurus*) | Australia | 2010 | P | Farmed | < 2 months old | 196 | 144 (73.5) | n.s. | PCR | [182] |
| Cattle (*Bos taurus*) | Nepal | 2010 | P | Farmed | 2-6 months old | 81 | 28 (34.5) | n.s. | CM | [17] |
| Cattle (*Bos taurus*) | France | 2010-2011 | P | Farmed | 7-21 days old | 968 | 402 (41.5) | M | CM | [183] |
| Cattle (*Bos taurus*) | Vietnam | 2011 | P | Farmed | 2-6 months old | 232 | 44 (18.9) | n.s. | CM | [184] |
| Cattle (*Bos taurus*) | Brazil | 2006 | P | Farmed | All age groups | 200 | 28 (14.0) | M | PCR | [185] |
| Cattle (*Bos taurus*) | Pakistan | 2007-2008 | P | Farmed | No age group | 720 | 76 (10.5) | n.s. | CM | [19] |

**Additional file 2: Table S2.** (*Continuted*)

| **Host (Scientific name)** | **Country** | **Period of study** | **Study**  **type** | **Keeping status** | **Age group(s)** | **Sample size** | **No. positive (%)** | **Sign^a^** | **Technique(s)** | **Reference Number** |
| --- | --- | --- | --- | --- | --- | --- | --- | --- | --- | --- |
| Cattle (*Bos indicus*) | Ethiopia | 2009 | P | Farmed | No age group | 384 | 30 (7.8) | n.s. | CM | [186] |
| Cattle (*Bos taurus*) | Ghana | 2009 | P | Farmed | 3 age groups | 241 | 70 (29.0) | M | CM | [187] |
| Cattle (*Bos taurus*) | Poland | 2009 | P | Farmed | 5 age groups | 700 | 119 (17.0) | M | PCR | [188] |
| Cattle (*Bos taurus*) | India | 2009-2010 | P | Farmed | < 6 months old | 459 | 182 (39.6) | M | PCR | [189] |
| Cattle (*Bos taurus*) | India | 2009-2010 | P | Farmed | < 5 months old | 144 | 56 (38.9) | M | CM | [190] |
| Cattle (*Bos taurus*) | Sweden | 2009-2010 | P | Farmed | 2 age groups | 480 | 116 (24.1) | M | IFA | [191] |
| Cattle (*Bos taurus*) | China | 2009-2011 | P | Farmed | 24-60 days old | 151 | 72 (47.6) | n.s. | PCR | [192] |
| Cattle (*Bos taurus*) | Egypt | 2009-2011 | P | Farmed | 4 age groups | 1,974 | 269 (13.6) | n.s. | CM | [193] |
| Cattle (*Bos taurus*) | India | 2009-2012 | P | n.s. | < 3 months old | 461 | 75 (16.3) | M | CM | [20] |
| Cattle (*Bos indicus*) | Ethiopia | 2010-2011 | P | Farmed | 3 age groups | 133 | 37 (27.8) | n.s. | CM | [194] |
| Cattle (*Bos taurus*) | China | 2010-2012 | P | Farmed | 4 age groups | 847 | 38 (4.4) | n.s. | CM | [195] |
| Cattle (*Bos taurus*) | China | 2010-2012 | P | Farmed | 4 age groups | 1,224 | 32 (2.6) | n.s. | CM | [195] |
| Cattle (*Bos taurus*) | Egypt | 2011 | P | Farmed | 4 age groups | 593 | 185 (31.2) | M | PCR | [23] |
| Cattle (*Bos taurus*) | Iran | 2012 | P | Farmed | Suckling calves | 195 | 25 (12.8) | M | CM | [196] |
| Cattle (*Bos taurus*) | Japan | 2012 | P | Farmed | 2-120 days old | 107 | 25 (23.0) | M | PCR | [197] |
| Cattle (*Bos indicus*) | Nigeria | n.s. | P | Farmed | 6 age groups | 250 | 70 (28.0) | n.s. | CM | [198] |
| Cattle (*Bos taurus*) | Brazil | n.s. | P | Farmed | < 3 months old | 100 | 16 (16.0) | M | ICT | [199] |
| Cattle (*Bos taurus*) | India | n.s. | P | Farmed | 1-2 months old | 16 | 2 (12.5) | A | ICT | [21] |
| Cattle (*Bos taurus*) | Iran | n.s. | P | Farmed | < 1 months old | 300 | 85 (28.3) | M | CM | [200] |
| Cattle (*Bos taurus*) | Rwanda | 2010 | P | n.s. | 2 age groups | 135 | 4 (3.0) | n.s. | IFA | [37] |
| Cattle (*Bos taurus*) | Egypt | 2010-2011 | P | Farmed | 3 age groups | 1,697 | 118 (7.0) | n.s. | CM | [24] |
| Cattle (*Bos taurus*) | Iran | 2010-2011 | P | Farmed | 4 age groups | 246 | 55 (22.3) | M | CM | [201] |
| Cattle (*Bos indicus*) | Myanmar | 2011 | P | Farmed | 3 age groups | 400 | 224 (56.0) | n.s. | CM | [202] |
| Cattle (*Bos indicus*) | Nigeria | 2011 | P | Pastured | No age group | 100 | 33 (33.0) | M | CM | [203] |
| Cattle (*Bos taurus*) | China | 2011-2012 | P | Farmed | 4 age groups | 1,366 | 23 (1.68) | n.s. | CM | [204] |
| Cattle (*Bos taurus*) | Germany | 2012 | P^d^ | n.s. | < 1 year old | 1,564 | 646 (41.3) | S | CM | [205] |
| Cattle (*Bos taurus*) | China | 2012-2013 | P | Farmed | 12-14 months old | 420 | 87 (20.7) | n.s. | CM | [206] |
| Cattle (*Bos taurus*) | China | 2012-2013 | P | Farmed | 12-14 months old | 405 | 71 (17.5) | n.s. | CM | [206] |
| Cattle (*Bos taurus*) | Sri Lanka | 2012-2013 | P | Farmed | < 3 months old | 340 | 211 (62.1) | n.s. | PCR | [28] |
| Cattle (*Bos taurus*) | Bangladesh | 2012-2014 | P | Farmed | 3 age groups | 110 | 31 (28.1) | S | ICT | [207] |
| Cattle (*Bos taurus*) | China | 2013 | P | Farmed | 3 age groups | 252 | 53 (21.0) | A | CM | [208] |
| Cattle (*Bos taurus*) | Iran | 2013 | P | n.s. | 3 age groups | 217 | 8 (3.68) | A | CM | [209] |
| Cattle (*Bos taurus*) | Taiwan | n.s. | P | Farmed | 3 age groups | 1,256 | 410 (32.6) | n.s. | CM | [210] |
| Cattle (*Bos taurus*) | Madagascar | 2011 | P | n.s. | No age group | 62 | 18 (29.0) | n.s. | PCR | [211] |
| Cattle (*Bos taurus*) | New Zealand | 2011 | P | Farmed | 1-21 days old | 1,283 | 176 (13.7) | n.s. | IFA | [212] |
| Cattle (*Bos indicus*) | Nigeria | 2012 | P | n.s. | 3 age groups | 95 | 41 (43.2) | S | ELISA | [213] |
| Cattle (*Bos taurus*) | China | 2012-2013 | P | Farmed | Pre-weaned | 258 | 52 (20.2) | n.s. | CM | [214] |
| Cattle (*Bos taurus*) | Sweden | 2012-2013 | P | Farmed | 1-3 months old | 332 | 122 (36.7) | M | IFA | [215] |
| Cattle (*Bos taurus*) | Iran | 2013-2014 | P | n.s. | 2 age groups | 430 | 39 (9.07) | M | CM | [216] |

**Additional file 2: Table S2.** (*Continuted*)

| **Host (Scientific name)** | **Country** | **Period of study** | **Study**  **type** | **Keeping status** | **Age group(s)** | **Sample size** | | **No. positive (%)** | **Sign^a^** | **Technique(s)** | **Reference Number** |
| --- | --- | --- | --- | --- | --- | --- | --- | --- | --- | --- | --- |
| Cattle (*Bos taurus*) | Japan | 2013-2014 | P | Farmed | < 3 months old | | 77 | 44 (57.0) | n.s. | PCR | [217] |
| Cattle (*Bos taurus*) | Japan | 2014 | P | Farmed | > 5 months old | | 94 | 2 (2.1) | A | CM | [218] |
| Cattle (*Bos taurus*) | Nigeria | n.s. | P | Farmed | 2 age groups | | 400 | 89 (22.3) | n.s. | CM | [219] |
| Cattle (*Bos indicus*) | Nigeria | n.s. | P | n.s. | 2 age groups | | 350 | 98 (28.0) | M | CM | [220] |
| Cattle (*Bos taurus*) | South Africa | 2012 | P | Farmed | < 6 months old | | 352 | 2 (0.6) | n.s. | CM | [221] |
| Cattle (*Bos taurus*) | Spain | 2011-2012 | P | Farmed | 25-128 months | | 362 | 45 (12.4) | A | PCR | [222] |
| Cattle (*Bos taurus*) | India | 2012-2013 | P | n.s. | 2 age groups | | 145 | 50 (34.4) | M | CM | [31] |
| Cattle (*Bos taurus*) | Argentina | 2013-2014 | P | Farmed | 1-70 days old | | 552 | 88 (16.0) | A | CM | [223] |
| Cattle (*Bos taurus*) | China | 2014-2015 | P | Farmed | 3 age groups | | 822 | 21 (2.5) | n.s. | CM | [224] |
| Cattle (*Bos taurus*) | Egypt | 2014 | P | Farmed | 3 age groups | | 480 | 49 (10.2) | M | CM | [32] |
| Cattle (*Bos indicus*) | Ethiopia | 2014 | P | Farmed | < 5 months old | | 449 | 71 (15.8) | n.s. | PCR | [225] |
| Cattle (*Bos taurus*) | Malaysia | 2015 | P | Farmed | 3 age groups | | 151 | 24 (15.9) | M | CM | [226] |
| Cattle (*Bos taurus*) | UK | n.s. | P | Farmed | Adults | | 308 | 13 (4.13) | M | PCR | [227] |
| Cattle (*Bos taurus*) | UK | n.s. | P | Farmed | Calves | | 190 | 115 (60.5) | M | PCR | [227] |
| Eland (*Taurotragus oryx*) | USA | n.s. | O | Captive | No age group | | 4 | 1 (25.0) | n.s. | CM | [1] |
| Gaur (*Bos gaurus*) | USA | n.s. | O | Captive | No age group | | 1 | 00 (00.0) | n.s. | CM | [1] |
| [Gazelle](https://en.wikipedia.org/wiki/Gazella) (*Gazella dorcas*) | Spain | n.s. | O | Captive | No age group | | 3 | 1 (33.3) | A | CM | [34] |
| [Gazelle](https://en.wikipedia.org/wiki/Gazella) (*Gazella dama*) | USA | n.s. | O | Captive | No age group | | 8 | 1 (12.5) | n.s. | CM | [1] |
| [Gazelle](https://en.wikipedia.org/wiki/Gazella) (*Gazella granti roosevelti*) | USA | n.s. | O | Captive | No age group | | 2 | 00 (00.0) | n.s. | CM | [1] |
| Gazelle (*Gazella leptoceros*) | USA | n.s. | O | Captive | No age group | | 6 | 4 (66.7) | n.s. | CM | [1] |
| Gazelle (*Gazella subgutturosa*) | USA | n.s. | O | Captive | No age group | | 10 | 1 (10.0) | n.s. | CM | [1] |
| Gemsbok (*Oryx gazella gazella*) | USA | n.s. | O | Captive | No age group | | 2 | 00 (00.0) | n.s. | CM | [1] |
| Gayal (*Bos frontalis*) | India | n.s. | P | Pastured | 3 age groups | | 157 | 88 (56.0) | M | ELISA | [228] |
| Goat (*Capra hircus*) | Spain | n.s. | P | Farmed | No age group | | 367 | 40 (11.0) | n.s. | CM | [229] |
| Goat (*Capra hircus*) | Trinidad | n.s. | O | n.s. | < 12 weeks old | | 20 | 4 (20.0) | M | CM | [44] |
| Goat (*Capra hircus*) | USA | 1992-1994 | O^d^ | n.s. | No age group | | 19 | 5 (26.3) | n.s. | CM | [51] |
| Goat (*Capra hircus*) | Spain | 1988-1992 | O | Farmed | 1-45 days old | | 36 | 15 (42.0) | S | CM | [230] |
| Goat (*Capra hircus*) | Brazil | 1994-1995 | O^c^ | n.s. | 1-2 weeks old | | 22 | 22 (100) | M | CM | [231] |
| Goat (*Capra hircus*) | Sri Lanka | 1999 | P | Farmed | 3 age groups | | 1,020 | 291 (28.5) | M | CM | [232] |
| Goat (*Capra hircus*) | Poland | n.s. | P | Farmed | 1-7 years old | | 46 | 00 (0.0) | A | CM | [233] |
| Goat (*Capra hircus*) | Iraq | n.s. | P | n.s. | No age group | | 45 | 6 (13.3) | n.s. | CM | [68] |
| Goat (*Capra hircus*) | France | 2003 | P | Farmed | Adults | | 100 | 13 (13.0) | A | IFA | [234] |
| Goat (*Capra hircus*) | Brazil | n.s. | P | Farmed | 2 age groups | | 105 | 5 (4.8) | n.s. | CM | [235] |
| Goat (*Capra hircus*) | France | n.s. | P | Farmed | 5-12 months old | | 200 | 5 (2.5) | M | CM | [236] |
| Goat (*Capra hircus*) | Taiwan | n.s. | P | Farmed | 5 age groups | | 123 | 44 (35.8) | M | CM | [83] |
| Goat (*Capra hircus*) | South Korea | 2000 | O | Farmed | No age group | | 7 | 3 (42.9) | n.s. | CM | [237] |
| Goat (*Capra hircus*) | France | 2003 | P | Farmed | 5-30 days old | | 879 | 142 (16.2) | n.s. | CM | [238] |
| Goat (*Capra hircus*) | Nigeria | 2005 | P | Slaughtered | Adults | | 150 | 5 (3.3) | M | CM | [239] |
| Goat (*Capra hircus*) | Argentina | n.s. | P | n.s. | No age group | | 31 | 21 (67.7) | n.s. | CM | [88] |
| Goat (*Capra hircus*) | Serbia | n.s. | P | n.s. | 2 age groups | | 88 | 28 (31.8) | M | CM | [240] |
| Goat (*Capra hircus*) | Tunisia | 2003-2004 | P | Farmed | 1-7 years old | | 184 | 00 (00.0) | n.s. | CM | [101] |

**Additional file 2: Table S2.** (*Continuted*)

| **Host (Scientific name)** | **Country** | **Period of study** | **Study**  **type** | | **Keeping status** | **Age group(s)** | **Sample size** | | **No. positive (%)** | **Sign^a^** | **Technique(s)** | **Reference Number** |
| --- | --- | --- | --- | --- | --- | --- | --- | --- | --- | --- | --- | --- |
| Goat (*Capra hircus*) | Zambia | 2003-2004 | | P | Farmed | < 3 months old | | 105 | 5 (4.8) | n.s. | ELISA | [241] |
| Goat (*Capra hircus*) | Spain | 2005 | | P | Farmed | 2 age groups | | 184 | 25 (13.6) | M | IFA | [242] |
| Goat (*Capra hircus*) | China | 2006 | | O | Farmed | No age group | | 42 | 15 (35.7) | n.s. | IFA | [2] |
| Goat (*Capra hircus*) | Spain | n.s. | | P | Farmed | No age group | | 116 | 9 (7.7) | A | IFA | [110] |
| Goat (*Capra hircus*) | Turkey | 2006 | | P | Slaughtered | No age group | | 56 | 6 (10.7) | n.s. | ICT | [119] |
| Goat (*Capra hircus*) | India | 2006-2007 | | O | Farmed | < 1 months old | | 16 | 0 (00.0) | n.s. | IFA | [121] |
| Goat (*Capra hircus*) | Belgium | n.s. | | P | Farmed | < 10 weeks old | | 148 | 14 (9.5) | n.s. | IFA | [243] |
| Goat (*Capra hircus*) | Malavi | 2001-2003 | | P | Farmed | No age group | | 225 | 23 (10.2) | n.s. | CM | [131] |
| Goat (*Capra hircus*) | Spain | 2004-2005 | | P^b^ | Farmed | 3 age groups | | 582 | 111 (19.1) | n.s. | CM | [244] |
| Goat (*Capra hircus*) | Romania | 2007-2008 | | P | Farmed | 4 age groups | | 412 | 99 (24.0) | M | CM | [245] |
| Goat (*Capra hircus*) | England & Wales | 2004-2006 | | O | Farmed | No age group | | 15 | 3 (20.0) | n.s. | IFA | [148] |
| Goat (*Capra hircus*) | Spain | n.s. | | O | Farmed | < 21 days old | | 5 | 2 (40.0) | S | CM | [246] |
| Goat (*Capra hircus*) | Cyprus | 2008-2009 | | O | Flocked | Kids | | 75 | 65 (86.6) | S | ELISA | [247] |
| Goat (*Capra hircus*) | India | 2009-2012 | | P | Farmed | < 3 months old | | 116 | 4 (3.5) | M | CM | [20] |
| Goat (*Capra hircus*) | Ethiopia | 2010-2011 | | P | Farmed | 3 age groups | | 41 | 5 (12.2) | n.s. | CM | [193] |
| Goat (*Capra hircus*) | France | 2011 | | P^b^ | Farmed | ≤ 3 months old | | 25 | 17 (68.0) | n.s. | IFA | [248] |
| Goat (*Capra hircus*) | France | 2011 | | P^b^ | Farmed | ≤ 3 months old | | 15 | 14 (93.0) | n.s. | IFA | [248] |
| Goat (*Capra hircus*) | Iran | 2012 | | P | Farmed | No age group | | 237 | 6 (2.5) | M | CM | [195] |
| Goat (*Capra hircus*) | Nigeria | n.s. | | P | Farmed | 3 age groups | | 150 | 36 (24.0) | n.s. | CM | [197] |
| Goat (*Capra hircus*) | China | 2006-2007 | | P | Farmed | 3 age groups | | 1,017 | 28 (2.7) | n.s. | CM | [249] |
| Goat (*Capra hircus*) | China | 2007-2013 | | P | Farmed | 4 age groups | | 604 | 69 (11.4) | n.s. | PCR | [250] |
| Goat (*Capra hircus*) | China | 2011 | | P | Farmed | 3 age groups | | 248 | 16 (6.4) | n.s. | CM | [249] |
| Goat (*Capra hircus*) | Papua New Guinea | 2011 | | P | Farmed | No age group | | 228 | 10 (4.4) | n.s. | PCR | [251] |
| Goat (*Capra hircus*) | France | 2012 | | P^b^ | Farmed | 1-5 years old | | 18 | 16 (89.0) | A | IFA | [252] |
| Goat (*Capra hircus*) | Greece | n.s. | | P | Farmed | < 10 weeks old | | 255 | 18 (7.1) | n.s. | IFA | [253] |
| Goat (*Capra hircus*) | Spain | 2008-2013 | | P | Flocked | < 35 days old | | 118 | 74 (62.7) | S | CM | [254] |
| Goat (*Capra hircus*) | Tanzania | 2010-2011 | | O | n.s. | No age group | | 56 | 5 (8.9) | n.s. | PCR | [255] |
| Goat (*Capra hircus*) | Botswana | 2010-2012 | | P | Farmed | 3 age groups | | 222 | 27 (12.2) | M | ELISA | [256] |
| Goat (*Capra hircus*) | Nigeria | 2012 | | P | n.s. | 3 age groups | | 51 | 39 (76.5) | S | ELISA | [212] |
| Goat (*Capra hircus*) | Nigeria | 2012-2014 | | P | Farmed | 3 age groups | | 216 | 81 (37.5) | M | ELISA | [257] |
| Goat (*Capra hircus*) | Greece | 2013 | | P^d^ | Farmed | 4-15 days old | | 292 | 223 (76.4) | S | CM | [258] |
| Goat (*Capra hircus*) | Iran | 2013-2014 | | P | n.s. | 2 age groups | | 340 | 21 (6.1) | M | CM | [216] |
| Goat (*Capra hircus*) | Bangladesh | n.s. | | P^d^ | n.s. | 1-6 months old | | 100 | 15 (15.0) | S | CM | [259] |
| Goat (*Capra hircus*) | Nigeria | n.s. | | P | n.s. | 2 age groups | | 260 | 46 (17.7) | M | CM | [220] |
| Goat (*Capra hircus*) | Mexico | 2014 | | P | Farmed | 3 age groups | | 80 | 58 (72.5) | M | CM | [260] |
| Ibex (*Capra ibex*) | China | 2005 | | O | Captive | Adult | | 1 | 1 (100) | n.s. | IFA | [2] |
| Ibex (*Capra ibex*) | Switzerland | 2006-2008 | | P | Wild | No age group | | 86 | 4 (4.7) | n.s. | CM | [261] |
| Ibex (*Capra nubiana*) | USA | n.s. | | O | Captive | No age group | | 1 | 00 (00.0) | n.s. | CM | [1] |

**Additional file 2: Table S2.** (*Continuted*)

| **Host (Scientific name)** | **Country** | **Period of study** | **Study**  **type** | **Keeping status** | **Age group(s)** | **Sample size** | | **No. positive (%)** | **Sign^a^** | **Technique(s)** | | **Reference Number** |
| --- | --- | --- | --- | --- | --- | --- | --- | --- | --- | --- | --- | --- |
| Impala (*Aepyceros melampus*) | South Africa | 2008 | P | Wild | No age group | | 94 | 4 (4.2) | n.s. | CM | [36] | |
| Impala (*Aepyceros melampus*) | USA | n.s. | O | Captive | No age group | | 9 | 8 (88.9) | n.s. | CM | [1] | |
| Kob (*Kobus kob thomasi*) | USA | n.s. | O | Captive | No age group | | 1 | 00 (00.0) | n.s. | CM | [1] | |
| Kudu (*Tragelaphus strepsiceros*) | USA | n.s. | O | Captive | No age group | | 2 | 00 (00.0) | n.s. | CM | [1] | |
| Markhor (*Capra falconeri*) | USA | n.s. | O | Captive | No age group | | 4 | 3 (75.0) | n.s. | CM | [1] | |
| Marshbuck (*Tragelaphus spekii*) | USA | n.s. | O | Captive | No age group | | 1 | 00 (00.0) | n.s. | CM | [1] | |
| Mouflon (*Ovis* *gmelini*) | USA | n.s. | O | Captive | No age group | | 12 | 7 (58.3) | n.s. | CM | [1] | |
| Mouflon (*Ovis* *musimon*) | Czechia | n.s. | P | Wild | No age group | | 43 | 1 (2.3) | n.s. | PCR | [262] | |
| Muskox (*Ovibos moschatus*) | Norway | 2012 | P | Wild | 4 age groups | | 167 | 17 (10.1) | n.s. | IFA | [263] | |
| Nile lechwe (*Kobus megaceros*) | USA | n.s. | O | Captive | No age group | | 2 | 1 (50.0) | n.s. | CM | [1] | |
| Nilgai (*Boselaphus tragocamelus*) | USA | n.s. | O | Captive | No age group | | 20 | 5 (25.0) | n.s. | CM | [1] | |
| Nyala (*Tragelaphus angasii*) | USA | n.s. | O | Captive | No age group | | 1 | 00 (00.0) | n.s. | CM | [1] | |
| Oryx (*Oryx gazella callotis*) | USA | n.s. | O | Captive | No age group | | 1 | 1 (100) | n.s. | CM | [1] | |
| Oryx (*Oryx gazella dammah*) | USA | n.s. | O | Captive | No age group | | 2 | 2 (100) | n.s. | CM | [1] | |
| Saiga (*Saiga tatarica*) | USA | n.s. | O | Captive | No age group | | 1 | 00 (00.0) | n.s. | CM | [1] | |
| Sheep (*Ovis aries*) | Spain | 1987-1989 | P | Farmed | 2 age groups | | 69 | 1 (1.4) | M | CM | [42] | |
| Sheep (*Ovis aries*) | Iran | 1990 | P | Flocked | Adults | | 215 | 37 (17.2) | A | CM | [264] | |
| Sheep (*Ovis aries*) | USA | 1992 | P^c^ | Farmed | 3 age groups | | 55 | 31 (56.3) | S | IFA | [265] | |
| Sheep (*Ovis aries*) | Iran | 1990-1991 | P | Flocked | Adults | | 433 | 18 (4.1) | A | CM | [266] | |
| Sheep (*Ovis aries*) | Iran | n.s. | P | Slaughtered | All age groups | | 510 | 137 (26.8) | n.s. | CM | [267] | |
| Sheep (*Ovis aries*) | Trinidad | 1992 | P | Farmed | ≤ 6 months old | | 90 | 18 (20.0) | M | CM | [45] | |
| Sheep (*Ovis aries*) | Spain | 1988-1992 | P | Farmed | 1-45 days old | | 183 | 82 (44.6) | S | CM | [230] | |
| Sheep (*Ovis aries*) | Canada | 1995 | P | Farmed | 2 age groups | | 89 | 21 (23.0) | n.s. | IFA | [53] | |
| Sheep (*Ovis aries*) | Poland | n.s. | P | Farmed | > 3 months old | | 159 | 16 (10.1) | A | CM | [233] | |
| Sheep (*Ovis aries*) | Spain | 1995-1997 | P | Farmed | < 3 months old | | 583 | 344 (59.0) | M | CM | [268] | |
| Sheep (*Ovis aries*) | Spain | 1995-1997 | P | Farmed | Adults | | 205 | 16 (7.5) | n.s. | CM | [268] | |
| Sheep (*Ovis aries*) | Poland | 1999-2001 | P | Farmed | Lambs | | 37 | 9 (24.3) | n.s. | ELISA | [269] | |
| Sheep (*Ovis aries*) | Iraq | n.s. | P | n.s. | No age group | | 45 | 8 (17.7) | n.s. | CM | [68] | |
| Sheep (*Ovis aries*) | Turkey | 2002 | P | Farmed | < 30 days old | | 144 | 67 (46.5) | M | CM | [270] | |
| Sheep (*Ovis aries*) | Mexico | n.s. | P | Flocked | 2 age groups | | 502 | 129 (25.7) | n.s. | CM | [271] | |
| Sheep (*Ovis aries*) | Australia | 2002-2003 | P | Slaughtered | No age group | | 500 | 131 (26.2) | M | PCR | [272] | |
| Sheep (*Ovis aries*) | Turkey | 2003 | P | Farmed | < 2 months old | | 471 | 43 (9.1) | n.s. | ELISA | [273] | |
| Sheep (*Ovis aries*) | Mexico | n.s. | P | Flocked | 2 age groups | | 1200 | 411 (34.3) | A | CM | [274] | |
| Sheep (*Ovis aries*) | Nigeria | 2005 | P | Slaughtered | Adults | | 150 | 2 (1.3) | M | CM | [239] | |
| Sheep (*Ovis aries*) | Argentina | n.s. | P | n.s. | No age group | | 22 | 4 (18.1) | n.s. | CM | [88] | |
| Sheep (*Ovis aries*) | Serbia | n.s. | P | n.s. | < 3 months old | | 126 | 53 (42.1) | A | CM | [240] | |
| Sheep (*Ovis aries*) | Tunisia | 2003-2004 | P | Farmed | 2 age groups | | 89 | 10 (11.2) | n.s. | CM | [101] | |
| Sheep (*Ovis aries*) | Zambia | 2003-2004 | P | Farmed | < 3 months old | | 152 | 19 (12.5) | n.s. | ELISA | [241] | |
| Sheep (*Ovis aries*) | Spain | 2005 | P | Farmed | 2 age groups | | 673 | 59 (8.8) | M | IFA | [242] | |
| Sheep (*Ovis aries*) | Spain | n.s. | P | Farmed | No age group | | 446 | 24 (5.3) | A | IFA | [110] | |
| Sheep (*Ovis aries*) | USA | n.s. | P^b^ | Farmed | Ewes | | 32 | 8 (25.0) | A | PCR | [275] | |
| Sheep (*Ovis aries*) | USA | n.s. | P^b^ | Farmed | < 21 days old | | 31 | 24 (77.4) | A | PCR | [275] | |

**Additional file 2: Table S2.** (*Continuted*)

| **Host (Scientific name)** | **Country** | **Period of study** | **Study**  **type** | **Keeping status** | **Age group(s)** | **Sample size** | | **No. positive (%)** | **Sign^a^** | **Technique(s)** | **Reference Number** |
| --- | --- | --- | --- | --- | --- | --- | --- | --- | --- | --- | --- |
| Sheep (*Ovis aries*) | UK | 2001-2005 | P^d^ | n.s. | < 3 weeks old | | 28 | 28 (100.0) | S | CM | [276] |
| Sheep (*Ovis aries*) | Spain | 2004-2006 | P | Farmed | < 21 days old | | 137 | 45 (2.8) | S | PCR | [277] |
| Sheep (*Ovis aries*) | UK | 2004-2006 | P | Farmed | All age groups | | 261 | 94 (36.0) | A | IFA | [276] |
| Sheep (*Ovis aries*) | Scotland | 2005-2006 | P | Farmed | 3-9 months old | | 8 | 5 (62.5) | A | IFA | [276] |
| Sheep (*Ovis aries*) | Turkey | 2006 | P | Slaughtered | No age group | | 167 | 22 (13.1) | n.s. | ICT | [119] |
| Sheep (*Ovis aries*) | England & Wales | 2006 | O^d^ | n.s. | < 3 months old | | 266 | 48 (18.0) | n.s. | IFA | [278] |
| Sheep (*Ovis aries*) | Greece | 2007 | P | Flocked | 3 age groups | | 523 | 152 (29.0) | M | CM | [279] |
| Sheep (*Ovis aries*) | Belgium | n.s. | P | Farmed | < 10 weeks old | | 137 | 18 (13.1) | n.s. | IFA | [243] |
| Sheep (*Ovis aries*) | Malavi | 2001-2003 | O | n.s. | No age group | | 5 | 00 (00.0) | n.s. | CM | [131] |
| Sheep (*Ovis aries*) | Turkey | 2005 | P | Farmed | < 2 months old | | 132 | 18 (13.6) | S | CM | [280] |
| Sheep (*Ovis aries*) | USA | 2005-2008 | O | n.s. | No age group | | 15 | 3 (20.0) | n.s. | PCR | [134] |
| Sheep (*Ovis aries*) | Australia | 2007 | P | Farmed | < 8 weeks old | | 477 | 117 (24.5) | n.s. | PCR | [281] |
| Sheep (*Ovis aries*) | Turkey | 2007 | P | Farmed | < 4 weeks old | | 400 | 155 (38.8) | S | CM | [282] |
| Sheep (*Ovis aries*) | England & Wales | 2004-2006 | P | Farmed | No age group | | 260 | 103 (39.6) | n.s. | IFA | [148] |
| Sheep (*Ovis aries*) | China | 2006-2007 | P | Farmed | 3 age groups | | 1,701 | 82 (4.8) | n.s. | PCR | [283] |
| Sheep (*Ovis aries*) | Brazil | 2007 | O | Farmed | No age group | | 11 | 00 (00.0) | n.s. | CM | [150] |
| Sheep (*Ovis aries*) | Spain | 2008-2009 | P | Farmed | Pre-weaned | | 127 | 39 (30.7) | S | CM | [156] |
| Sheep (*Ovis aries*) | Brazil | 2007 | P | Farmed | 2 age groups | | 125 | 2 (1.6) | n.s. | PCR | [284] |
| Sheep (*Ovis aries*) | Australia | 2009-2010 | P^b^ | Farmed | < 9 months old | | 107 | 87 (81.3) | n.s. | PCR | [285] |
| Sheep (*Ovis aries*) | Australia | 2009-2010 | P^b^ | Farmed | < 9 months old | | 119 | 85 (71.4) | n.s. | PCR | [285] |
| Sheep (*Ovis aries*) | Cyprus | 2008-2009 | O | Flocked | Lambs | | 39 | 30 (76.9) | S | ELISA | [248] |
| Sheep (*Ovis aries*) | Iran | 2009-2011 | O | n.s. | Adults | | 3 | 2 (66.6) | A | CM | [16] |
| Sheep (*Ovis aries*) | India | 2009-2012 | P | Farmed | < 3 months old | | 55 | 1 (1.8) | M | CM | [20] |
| Sheep (*Ovis aries*) | Ethiopia | 2010-2011 | P | Farmed | 3 age groups | | 63 | 14 (22.2) | n.s. | CM | [194] |
| Sheep (*Ovis aries*) | India | 2010-2011 | P | Flocked | < 6 months old | | 120 | 54 (45.0) | M | CM | [286] |
| Sheep (*Ovis aries*) | Nigeria | n.s. | P | n.s. | 3 age groups | | 150 | 24 (16.0) | n.s. | CM | [198] |
| Sheep (*Ovis aries*) | Romania | n.s. | P | Farmed | < 21 days old | | 175 | 24 (13.7) | S | CM | [287] |
| Sheep (*Ovis aries*) | Egypt | 2010-2011 | P | Farmed | 3 age groups | | 120 | 3 (2.5) | n.s. | CM | [24] |
| Sheep (*Ovis aries*) | Papua New Guinea | 2011 | P | Farmed | Adults | | 276 | 6 (2.2) | n.s. | PCR | [251] |
| Sheep (*Ovis aries*) | Iran | 2011-2012 | P | n.s. | No age group | | 1,749 | 198 (11.3) | A | CM | [288] |
| Sheep (*Ovis aries*) | Australia | n.s. | P^b^ | Farmed | 3 age groups | | 3,412 | 576 (16.9) | n.s. | PCR | [289] |
| Sheep (*Ovis aries*) | Brazil | n.s. | P | Farmed | 3 age groups | | 100 | 25 (25.0) | n.s. | PCR | [290] |
| Sheep (*Ovis aries*) | Greece | n.s. | P | Farmed | < 10 weeks old | | 429 | 22 (5.1) | n.s. | IFA | [253] |
| Sheep (*Ovis aries*) | Spain | 2008-2013 | P | Farmed | < 35 days old | | 171 | 54 (31.6) | S | CM | [254] |
| Sheep (*Ovis aries*) | Tanzania | 2010-2011 | O | n.s. | No age group | | 9 | 2 (22.2) | n.s. | PCR | [255] |
| Sheep (*Ovis aries*) | Botswana | 2010-2012 | P | Farmed | 3 age groups | | 166 | 22 (13.3) | M | ELISA | [256] |
| Sheep (*Ovis aries*) | Brazil | 2011 | P | Farmed | < 1 year old | | 193 | 29 (15.0) | M | PCR | [291] |
| Sheep (*Ovis aries*) | Nigeria | 2012 | P | n.s. | 3 age groups | | 79 | 33 (41.8) | S | ELISA | [213] |
| Sheep (*Ovis aries*) | Iran | 2013-2014 | P | n.s. | 2 age groups | | 345 | 20 (5.8) | M | CM | [216] |

**Additional file 2: Table S2.** (*Continuted*)

| **Host (Scientific name)** | **Country** | **Period of study** | **Study**  **type** | | **Keeping status** | **Age group(s)** | **Sample size** | | **No. positive (%)** | **Sign^a^** | **Technique(s)** | **Reference Number** |
| --- | --- | --- | --- | --- | --- | --- | --- | --- | --- | --- | --- | --- |
| Sheep (*Ovis aries*) | Australia | 2014 | | n.s. | Saleyard | 9-15 months old | | 474 | 31 (6.5) | n.s. | PCR | [292] |
| Sheep (*Ovis aries*) | Nigeria | n.s. | | P | n.s. | 2 age groups | | 290 | 34 (11.7) | M | CM | [220] |
| Sheep (*Ovis aries*) | China | 2013-2015 | | P | n.s. | 3 age groups | | 350 | 43 (12.3) | n.s. | PCR | [293] |
| Sheep (*Ovis aries*) | Mexico | 2014 | | P | Farmed | 3 age groups | | 80 | 54 (67.5) | M | CM | [260] |
| Springbok (*Antidorcas marsupialis*) | USA | n.s. | | O | Captive | No age group | | 14 | 1 (7.1) | n.s. | CM | [1] |
| Takin (*Budorcas taxicolor*) | China | 2005 | | O | Captive | Adult | | 1 | 1 (100) | n.s. | IFA | [2] |
| Waterbuck (*Kobus ellipsiprymnus*) | USA | n.s. | | O | Captive | No age group | | 2 | 00 (00.0) | n.s. | CM | [1] |
| Waterbuck (*Kobus ellipsiprymnus*) | Spain | n.s. | | O | Captive | No age group | | 3 | 1 (33.3) | n.s. | CM | [34] |
| Waterbuck (*Kobus ellipsiprymnus*) | Brazil | 2008 | | O | Captive | No age group | | 1 | 1 (100) | n.s. | CM | [3] |
| Wildebeest (*Connochaetes gnou*) | Tanzania | 1995 | | P | Wild | No age group | | 26 | 7 (27.0) | n.s. | CM | [35] |
| Wildebeest (*Connochaetes taurinus*) | USA | n.s. | | O | Captive | No age group | | 1 | 00 (00.0) | n.s. | CM | [1] |
| Wildebeest (*Connochaetes taurinus*) | Spain | n.s. | | O | Captive | No age group | | 3 | 1 (33.3) | n.s. | CM | [34] |
| Yak (*Bos mutus*) | China | 2005 | | O | Captive | Adult | | 1 | 1 (100) | n.s. | IFA | [2] |
| Yak (*Bos grunniens*) | China | 2006 | | O | Farmed | No age group | | 16 | 2 (12.5) | n.s. | IFA | [2] |
| Yak (*Bos grunniens*) | China | n.s. | | P | Fa & Ca | No age group | | 402 | 42 (10.4) | n.s. | CM | [294] |
| Yak (*Bos grunniens*) | China | n.s. | | P | Farmed | No age group | | 1,094 | 368 (33.64) | n.s. | ELISA | [295] |
| Yak (*Bos grunniens*) | China | 2008-2012 | | P | Farmed | 3 age groups | | 586 | 142 (24.2) | n.s. | CM | [296] |
| Yak (*Bos grunniens*) | China | 2013 | | P | Farmed | 3 age groups | | 327 | 98 (30.0) | n.s. | PCR | [297] |
| Yak (*Bos grunniens*) | China | 2013 | | P | Farmed | No age group | | 76 | 4 (5.26) | n.s. | PCR | [298] |
| Yak (*Bos grunniens*) | China | 2009-2012 | | P | Farmed | 2 age groups | | 545 | 22 (4.0) | A | PCR | [299] |
| Yak (*Bos grunniens*) | China | 2013-2015 | | P | Farmed | 3 age groups | | 554 | 158 (28.5) | n.s. | PCR | [300] |
| Zulu suni (*Nesotragus moschatus*) | USA | n.s. | | O | Captive | No age group | | 1 | 00 (00.0) | n.s. | CM | [1] |
| **Family Camelidae** |  |  | |  |  |  | |  |  |  |  |  |
| Alpaca (*Lama pacos*) | USA | 2004 | | O^c^ | Farmed | Crias | | 6 | 5 (83.3) | M | CM | [301] |
| Alpaca (*Lama pacos*) | USA | 2000-2004 | | O^d^ | n.s. | 6-180 days old | | 20 | 20 (100) | S | CM | [302] |
| Alpaca (*Lama pacos*) | UK | 2005-2006 | | O | Farmed | 2 age groups | | 31 | 4 (12.9) | M | IFA | [303] |
| Alpaca (*Lama pacos*) | Peru | 1995 | | P | Herded | 1-15 days old | | 241 | 24 (10.0) | n.s. | CM | [304] |
| Alpaca (*Lama pacos*) | Peru | n.s. | | P | Herded | 1-15 days old | | 5,163 | 666 (12.9) | n.s. | CM | [304] |
| Alpaca (*Lama pacos*) | USA | 2009 | | P | Farmed | 2 age groups | | 220 | 17 (7.7) | A | IFA | [305] |
| Alpaca (*Lama pacos*) | Peru | 2009-2010 | | P | Herded | No age group | | 274 | 12 (4.4) | n.s. | IFA | [306] |
| Alpaca (*Lama pacos*) | UK | 2012 | | O | Carcase | 8 months old | | 1 | 1 (100) | S | CM | [307] |
| Alpaca (*Lama pacos*) | Peru | 2010 | | O^c^ | Herded | 1-5 weeks old | | 50 | 10 (20.0) | S | CM | [308] |
| Camel (*Camelus bactrianus*) | USA | n.s. | | O | Wild | 4 years old | | 1 | 1 (100.0) | S | CM | [309] |
| Camel (*Camelus dromedarius*) | Iraq | n.s. | | O | n.s. | No age group | | 23 | 00 (00.0) | n.s. | CM | [68] |
| Camel (*Camelus dromedarius*) | Tunisia | 2003-2004 | | P | Farmed | 3-8 months old | | 110 | 00 (00.0) | n.s. | CM | [101] |
| Camel (*Camelus dromedarius*) | Iran | 2007-2008 | | P | Slaughtered | No age group | | 306 | 6 (1.9) | n.s. | CM | [310] |
| Camel (*Camelus dromedarius*) | Iran | n.s. | | P | Slaughtered | 3 age groups | | 103 | 39 (37.9) | n.s. | CM | [311] |
| Camel (*Camelus dromedarius*) | Iran | n.s. | | P | Herded | 2-12 years old | | 65 | 11 (16.9) | n.s. | CM | [312] |
| Camel (*Camelus bactrianus*) | Brazil | 2008 | | O | Captive | No age group | | 2 | 00 (00.0) | n.s. | CM | [3] |
| Camel (*Camelus dromedarius*) | Iran | 2009-2010 | | P | Farmed | 4 age groups | | 170 | 17 (10.0) | n.s. | CM | [313] |
| Camel (*Camelus dromedarius*) | Iran | 2008-2010 | | P | Far & Slu | 3 age groups | | 300 | 61 (20.3) | A | CM | [314] |
| Camel (*Camelus dromedarius*) | Iran | 2008-2010 | | P | Slaughtered | 3 age groups | | 100 | 12 (12.0) | A | CM | [314] |
| Camel (*Camelus bactrianus*) | China | n.s. | | O | Captive | No age group | | 2 | 1 (50.0) | n.s. | CM | [315] |

**Additional file 2: Table S2.** (*Continuted*)

| **Host (Scientific name)** | **Country** | **Period of study** | **Study**  **type** | | **Keeping status** | **Age group(s)** | **Sample size** | | **No. positive (%)** | **Sign^a^** | **Technique(s)** | **Reference Number** |
| --- | --- | --- | --- | --- | --- | --- | --- | --- | --- | --- | --- | --- |
| Guanaco (*Lama guanicoe*) | China | 2005 | | O | Captive | Adults | | 1 | 1 (100) | n.s. | IFA | [2] |
| Guanaco (*Lama guanicoe*) | Brazil | 2008 | | O | Captive | No age group | | 2 | 00 (00.0) | n.s. | CM | [3] |
| Llama (*Lama glama*) | USA | n.s. | | P | Farmed | < 23 years old | | 354 | 00 (00.0) | n.s. | IFA | [316] |
| Llama & Alpaca | USA | n.s. | | O | Herded | < 7 months old | | 45 | 4 (9.0) | S | CM | [317] |
| Llama (*Lama glama*) | Brazil | 2008 | | O | Captive | No age group | | 2 | 00 (00.0) | n.s. | CM | [3] |
| **Family Cervidae** |  |  | |  |  |  | |  |  |  |  |  |
| Black-tailed deer (*Odocoileus columbianus*) | USA | n.s. | | O | Wild | 1-6 years old | | 38 | 3 (7.9) | A | IFA | [318] |
| Caribou (*Rangifer tarandus*) | USA | n.s. | | O | Herded | No age group | | 49 | 3 (6.2) | A | PCR | [319] |
| Caribou (*Rangifer tarandus*) | Finland | 2001-2002 | | P | Wild | All age groups | | 664 | 00 (00.0) | n.s. | IFA | [320] |
| Caribou (*Rangifer tarandus*) | Norway | 2001-2002 | | P | Wild | All age groups | | 915 | 00 (00.0) | n.s. | IFA | [320] |
| Caribou (*Rangifer tarandus*) | Norway | 2001-2003 | | P | Wild | 3 age groups | | 155 | 00 (00.0) | A | CM | [321] |
| Caribou (*Rangifer tarandus*) | Canada | 2003-2006 | | P | Wild | Adults | | 149 | 2 (1.3) | n.s. | CM | [322] |
| Cheetal (*Axis axis*) | USA | n.s. | | O | Captive | No age group | | 12 | 1 (8.3) | n.s. | CM | [1] |
| Deer (species not stated) | Canada | 1998-2000 | | P | Wild | No age group | | 649 | 1 (0.15) | n.s. | IFA | [4] |
| Deer (species not stated) | South Korea | 2001-2002 | | P | n.s. | No age group | | 13 | 9 (69.2) | n.s. | CM | [77] |
| Eld's deer (*Cervus eldi thamin*) | USA | n.s. | | O | Captive | No age group | | 2 | 1 (50.0) | n.s. | CM | [1] |
| Eld's deer (*Cervus eldi thamin*) | Poland | n.s. | | O | Captive | Adult | | 1 | 1 (100) | A | CM | [323] |
| Elk (*Cervus e. nannodes*) | USA | n.s. | | O | Wild | 1-6 years old | | 40 | 8 (20.0) | A | IFA | [318] |
| Elk (*Cervus e. nannodes*) | Canada | 1998-2000 | | P | Ranched | No age group | | 72 | 2 (2.7) | n.s. | IFA | [4] |
| Fallow deer (*Dama dama*) | USA | n.s. | | O | Captive | No age group | | 8 | 2 (25.0) | n.s. | CM | [1] |
| Fallow deer (*Dama dama*) | USA | n.s. | | O | Wild | 1-6 years old | | 4 | 00 (00.0) | A | IFA | [318] |
| Fallow deer (*Dama dama*) | Britain | n.s. | | O | Wild | No age group | | 16 | 1 (6.0) | A | IFA | [324] |
| Fallow deer (*Dama dama*) | Portugal | 1999-2000 | | O | Wild | < 4 years old | | 12 | 12 (100) | A | CM | [325] |
| Fallow deer (*Dama dama*) | China | 2005 | | O | Captive | Adult | | 1 | 1 (100) | n.s. | IFA | [2] |
| Fallow deer (*Dama dama*) | Brazil | 2008 | | O | Captive | No age group | | 2 | 2 (100) | n.s. | CM | [3] |
| Fallow deer (*Dama dama*) | Czechia | n.s. | | O | Wild | No age group | | 64 | 00 (00.0) | n.s. | CM | [262] |
| Moose (*Alces alces*) | Canada | 1998-2000 | | O | Wild | No age group | | 177 | 00 (00.0) | n.s. | IFA | [4] |
| Moose (*Alces alces*) | Norway | 2000 | | P | Wild | No age group | | 72 | 00 (00.0) | A | IFA | [324] |
| Moose (*Alces alces*) | Norway | 2001-2003 | | P | Wild | 3 age groups | | 455 | 15 (3.3) | A | CM | [321] |
| Mule deer (*Odocoileus hemionus*) | USA | n.s. | | O | Captive | No age group | | 2 | 1 (50.0) | n.s. | CM | [1] |
| Muntjac deer (*Muntiacus reevesi*) | USA | n.s. | | O | Captive | No age group | | 1 | 00 (00.0) | n.s. | CM | [1] |
| Muntjac deer (*Muntiacus reevesi*) | Britain | n.s. | | O | Wild | No age group | | 42 | 4 (10.0) | A | IFA | [324] |
| Pere David’s deer (*Elaphurus davidianus*) | China | 2002-2007 | | O | Farmed | 2 years old | | 33 | 00 (00.0) | n.s. | PCR | [326] |
| Red deer (*Cervus elaphus*) | USA | n.s. | | O | Captive | No age group | | 2 | 2 (100) | n.s. | CM | [1] |
| Red deer (*Cervus elaphus*) | Norway | 2001-2003 | | P | Wild | 3 age groups | | 289 | 1 (0.3) | A | CM | [321] |
| Red deer (*Cervus elaphus*) | Poland | 2003-2005 | | P | Wild | No age group | | 118 | 17 (14.4) | A | CM | [6] |
| Red deer (*Cervus elaphus*) | China | 2002-2007 | | O | Farmed | 2 age groups | | 8 | 00 (00.0) | n.s. | PCR | [326] |
| Red deer (*Cervus elaphus*) | Brazil | 2008 | | O | Captive | No age group | | 2 | 00 (00.0) | n.s. | CM | [3] |
| Red deer (*Cervus elaphus*) | Czechia | n.s. | | P | Wild | No age group | | 136 | 6 (4.4) | n.s. | PCR | [262] |
| Red deer (*Cervus elaphus*) | Scotland | 2014 | | O^h^ | Wild | No age group | | 20 | 16 (80.0) | n.s. | PCR | [327] |
| Roe deer (*Capreolus capreolus*) | Norway | 2001-2003 | | P | Wild | 3 age groups | | 291 | 18 (6.2) | A | CM | [321] |
| Roe deer (*Capreolus capreolus*) | Poland | 2003 | | P | Wild | No age group | | 22 | 2 (9.1) | A | CM | [6] |
| Roe deer (*Capreolus capreolus*) | Spain | 2008-2009 | | P | Wild | No age group | | 224 | 3 (1.3) | A | CM | [328] |
| Roe deer (*Capreolus capreolus*) | Spain | n.s. | | P | Wild | No age group | | 212 | 9 (4.2) | n.s. | CM | [329] |
| Roe deer (*Capreolus capreolus*) | Scotland | 2014 | | O^h^ | Wild | No age group | | 6 | 2 (33.0) | n.s. | PCR | [327] |

**Additional file 2: Table S2.**  (*Continuted*)

| **Host (Scientific name)** | **Country** | **Period of study** | **Study**  **type** | **Keeping status** | **Age group(s)** | **Sample size** | | **No. positive (%)** | **Sign^a^** | **Technique(s)** | **Reference Number** |
| --- | --- | --- | --- | --- | --- | --- | --- | --- | --- | --- | --- |
| Sambar (*Rusa unicolor*) | Brazil | 2008 | O | Captive | No age group | | 2 | 2 (100) | n.s. | CM | [3] |
| Serow (*Capricornis crispus*) | USA | n.s. | O | Captive | No age group | | 2 | 00 (00.0) | n.s. | CM | [1] |
| Sika deer (*Cervus nippon*) | USA | n.s. | O | Captive | No age group | | 3 | 1 (33.3) | n.s. | CM | [1] |
| Sika deer (*Cervus nippon*) | China | 2002-2007 | O | Farmed | 4 age groups | | 83 | 2 (2.4) | n.s. | PCR | [326] |
| Swamp deer (*Rucervus duvaucelii*) | USA | n.s. | O | Captive | No age group | | 8 | 4 (50.0) | n.s. | CM | [1] |
| Swamp deer (*Rucervus duvaucelii*) | Nepal | 2010 | O | Wild | No age group | | 32 | 20 (62.5) | n.s. | CM | [17] |
| Thorold's deer (*Cervus albirostris*) | Poland | n.s. | O | Captive | Adult | | 1 | 1 (100) | n.s. | CM | [323] |
| Wapiti (*Cervus elaphus*) | China | 2005 | O | Captive | Adult | | 1 | 1 (100) | n.s. | IFA | [2] |
| White-lipped deer (*Cervus albirostris*) | China | 2005 | O | Captive | Adult | | 1 | 1 (100) | n.s. | IFA | [2] |
| White-tailed deer (*Odocoileus virginianus*) | USA | 1994 | O | Captive | 2 age groups | | 2 | 1 (50.0) | S | CM | [330] |
| White-tailed deer (*Odocoileus virginianus*) | USA | 1995 | O^b^ | Captive | 2 age groups | | 29 | 4 (13.8) | A | CM | [330] |
| White-tailed deer (*Odocoileus virginianus*) | USA | 1997 | P | Wild | 15 age groups | | 394 | 21 (5.3) | n.s. | IFA | [331] |
| White-tailed deer (*Odocoileus virginianus*) | USA | 1996-1998 | O^h^ | Wild | No age group | | 91 | 10 (10.9) | A | PCR | [332] |
| White-tailed deer (*Odocoileus virginianus*) | USA | 1996-1998 | O | Wild | 2 age groups | | 24 | 2 (8.3) | n.s. | ELISA | [333] |
| White-tailed deer (*Odocoileus virginianus*) | USA | 2005-2007 | O | Wild | No age group | | 59 | 5 (8.5) | n.s. | PCR | [334] |
| White-tailed deer (*Odocoileus virginianus*) | USA | 2005-2008 | O | Wild | No age group | | 91 | 2 (2.1) | n.s. | PCR | [134] |
| White-tailed deer (*Odocoileus virginianus*) | USA | 2010-2012 | O | Wild | No age group | | 80 | 10 (12.5) | A | PCR | [335] |
| White-tailed deer (*Odocoileus virginianus*) | Czechia | n.s. | O | Wild | No age group | | 26 | 3 (11.5) | n.s. | PCR | [262] |
| **Family Suidae** |  |  |  |  |  | |  |  |  |  |  |
| Feral pig (*Sus scrofa*) | USA | 1995 | P | Wild | All age groups | | 221 | 12 (5.4) | n.s. | IFA | [336] |
| Feral pig (*Sus scrofa*) | Australia | 2004 | P | Wild | No age group | | 292 | 1 (0.3) | n.s. | CM | [337] |
| Feral pig (*Sus scrofa*) | Brazil | 2008 | O | Captive | No age group | | 4 | 00 (00.0) | n.s. | CM | [3] |
| Pig (*Sus scrofa*) | Spain | 1987-1989 | P | Farmed | < 2 months old | | 329 | 10 (3.0) | M | CM | [42] |
| Pig (*Sus scrofa*) | South Korea | n.s. | P | n.s. | 6-8 months old | | 500 | 98 (19.9) | n.s. | CM | [41] |
| Pig (*Sus scrofa*) | Trinidad | n.s. | P | n.s. | < 12 weeks old | | 275 | 54 (19.6) | M | CM | [44] |
| Pig (*Sus scrofa*) | Trinidad | 1992 | P | Farmed | ≤ 6 months old | | 102 | 15 (14.7) | M | CM | [45] |
| Pig (*Sus scrofa*) | USA | 1992-1994 | O^d^ | n.s. | No age group | | 50 | 3 (6.0) | n.s. | CM | [51] |
| Pig (*Sus scrofa*) | Spain | n.s. | P | Farmed | All age groups | | 620 | 136 (21.9) | M | CM | [338] |
| Pig (*Sus scrofa*) | Canada | 1995 | P | Farmed | No age group | | 236 | 26 (11.0) | n.s. | IFA | [53] |
| Pig (*Sus scrofa*) | Canada | n.s. | P | Farmed | All age groups | | 1,602 | 45 (2.8) | n.s. | n.s. | [339] |
| Pig (*Sus scrofa*) | Japan | 1998-2000 | P | Farmed | 1-3 months old | | 232 | 77 (33.2) | n.s. | IFA | [340] |
| Pig (*Sus scrofa*) | Japan | 1998-2000 | P | Farmed | 6 months old | | 252 | 1 (0.4) | n.s. | IFA | [340] |
| Pig (*Sus scrofa*) | Germany | n.s. | P | Farmed | 2 age groups | | 287 | 4 (1.4) | S | CM | [341] |
| Pig (*Sus scrofa*) | Australia | 1999-2001 | P | Herded | < 9 weeks old | | 646 | 39 (6.03) | n.s. | CM | [342] |
| Pig (*Sus scrofa*) | Serbia | n.s. | P | Farmed | 5 age groups | | 290 | 89 (34.2) | M | CM | [343] |
| Pig (*Sus scrofa*) | Germany | 1998-2002 | O | n.s. | All age groups | | 1,427 | 2 (0.1) | A | CM | [76] |
| Pig (*Sus scrofa*) | South Korea | 2000-2001 | P | Farmed | No age group | | 589 | 62 (10.5) | n.s. | CM | [344] |
| Pig (*Sus scrofa*) | South Korea | 2001-2002 | P | n.s. | No age group | | 493 | 52 (10.5) | n.s. | CM | [77] |
| Pig (*Sus scrofa*) | Czechia | 2002-2004 | P | Farmed | 3 age groups | | 4,338 | 345 (9.0) | M | CM | [345] |
| Pig (*Sus scrofa*) | Denmark | 2003-2004 | P | Herded | 3 age groups | | 1,237 | 395 (31.9) | M | IFA | [93] |
| Pig (*Sus scrofa*) | Ireland | 2005 | P | Farmed | 5 age groups | | 342 | 39 (11.4) | A | CM | [346] |
| Pig (*Sus scrofa*) | Nigeria | 2005-2006 | P | Farmed | 2 age groups | | 402 | 56 (13.9) | n.s. | CM | [347] |
| Pig (*Sus scrofa*) | Australia | 2005-2006 | P | Herded | 3 age groups | | 289 | 64 (22.1) | M | PCR | [348] |

**Additional file 2: Table S2.** (*Continuted*)

| **Host (Scientific name)** | **Country** | **Period of study** | **Study**  **type** | **Keeping status** | **Age group(s)** | **Sample size** | | **No. positive (%)** | **Sign^a^** | **Technique(s)** | **Reference Number** |
| --- | --- | --- | --- | --- | --- | --- | --- | --- | --- | --- | --- |
| Pig (*Sus scrofa*) | Malavi | 2001-2003 | O | n.s. | No age group | | 92 | 30 (32.6) | n.s. | CM | [131] |
| Pig (*Sus scrofa*) | Czechia | 2007 | P | Farmed | 4 age groups | | 413 | 87 (21.1) | n.s. | CM | [349] |
| Pig (*Sus scrofa*) | Nigeria | 2008 | P | Farmed | < 20 weeks old | | 132 | 18 (13.6) | M | CM | [350] |
| Pig (*Sus scrofa*) | Czechia | n.s. | P | Slaughtered | 2 age groups | | 144 | 38 (26.3) | n.s. | CM | [351] |
| Pig (*Sus scrofa*) | China | 2006-2007 | P | Farmed | 4 age groups | | 1,350 | 111 (8.2) | n.s. | CM | [352] |
| Pig (*Sus scrofa*) | Brazil | 2007 | P | Farmed | No age group | | 25 | 00 (00.0) | n.s. | CM | [150] |
| Pig (*Sus scrofa*) | UK | 2007 | O^d^ | Farmed | 5 age groups | | 308 | 119 (38.6) | n.s. | IFA | [353] |
| Pig (*Sus scrofa*) | China | 2009 | P | Farmed | No age group | | 94 | 16 (17.0) | n.s. | PCR | [354] |
| Pig (*Sus scrofa*) | Brazil | n.s. | P | Farmed | No age group | | 91 | 2 (2.2) | n.s. | PCR | [355] |
| Pig (*Sus scrofa*) | Zambia | 2011 | P | Farmed | 4 age groups | | 217 | 96 (44.2) | M | IFA | [356] |
| Pig (*Sus scrofa*) | Czechia | 2009-2011 | P | Farmed | 5 age groups | | 1,620 | 353 (21.8) | n.s. | PCR | [357] |
| Pig (*Sus scrofa*) | India | 2009-2012 | P | Farmed | < 3 months old | | 42 | 8 (19.1) | M | CM | [20] |
| Pig (*Sus scrofa*) | China | 2011-2012 | P | Farmed | 3 age groups | | 113 | 63 (55.8) | n.s. | PCR | [358] |
| Pig (*Sus scrofa*) | Poland | 2008 | P | Farmed | > 5 months old | | 166 | 46 (27.7) | M | PCR | [359] |
| Pig (*Sus scrofa*) | Japan | 2009 | P | Farmed | 8 age groups | | 334 | 79 (23.7) | M | IFA | [360] |
| Pig (*Sus scrofa*) | Japan | 2010-2011 | P | Farmed | 5 age groups | | 344 | 112 (32.6) | M | IFA | [361] |
| Pig (*Sus scrofa*) | Madagascar | 2011 | O | n.s. | No age group | | 17 | 4 (23.5) | n.s. | PCR | [212] |
| Pig (*Sus scrofa*) | Denmark | 2011-2012 | P | Farmed | 4 age groups | | 856 | 350 (40.9) | n.s. | IFA | [362] |
| Pig (*Sus scrofa*) | China | 2011-2014 | P | Farmed | 4 age groups | | 1,337 | 44 (3.3) | n.s. | CM | [363] |
| Pig (*Sus scrofa*) | Nigeria | 2012 | P | n.s. | 3 age groups | | 98 | 44 (44.9) | S | ELISA | [212] |
| Wild boar (*Sus scrofa*) | Poland | 2003 | O | Wild | No age group | | 5 | 00 (00.0) | A | CM | [6] |
| Wild boar (*Sus scrofa*) | Spain | 2008-2009 | O | Wild | No age group | | 381 | 30 (7.6) | n.s. | IFA | [328] |
| Wild boar (*Sus scrofa*) | Czechia | 2009-2010 | P | Enclosed | Adults | | 193 | 32 (16.5) | n.s. | PCR | [364] |
| **Family Equidae** |  |  |  |  |  | |  |  |  |  |  |
| Donkey (*Equus asinus*) | USA | 2005-2008 | O | n.s. | No age group | | 6 | 00 (00.0) | n.s. | PCR | [134] |
| Donkey (*Equus asinus*) | Brazil | 2008 | O | Captive | No age group | | 2 | 00 (00.0) | n.s. | CM | [3] |
| Donkey (*Equus asinus*) | Algeria | 2011-2013 | P | Farmed | 3 age groups | | 124 | 2 (1.6) | n.s. | PCR | [365] |
| Horse (*Equus caballus*) | USA | 1992 | P | Farmed | 4 age groups | | 222 | 16 (7.2) | M | IFA | [366] |
| Horse (*Equus caballus*) | USA | 1993-1994 | P | n.s. | 4-24 years old | | 91 | 00 (00.0) | n.s. | IFA | [367] |
| Horse (*Equus caballus*) | Canada | 1995 | P | Farmed | No age group | | 35 | 6 (17.0) | n.s. | IFA | [53] |
| Horse (*Equus caballus*) | USA | 1996 | P | n.s. | 3-30 years old | | 300 | 1 (0.33) | n.s. | IFA | [368] |
| Horse (*Equus caballus*) | USA | n.s. | P | n.s. | No age group | | 58 | 00 (00.0) | n.s. | CM | [369] |
| Horse (*Equus caballus*) | Poland | n.s. | P | Farmed | No age group | | 106 | 10 (9.4) | n.s. | CM | [370] |
| Horse (*Equus caballus*) | Canada | 1998-2000 | O | Wild | Adult | | 1 | 00 (00.0) | n.s. | IFA | [4] |
| Horse (*Equus caballus*) | Poland | 1999-2001 | P | n.s. | Colts | | 26 | 3 (11.5) | n.s. | ELISA | [269] |
| Horse (*Equus caballus*) | Iraq | n.s. | P | n.s. | No age group | | 25 | 3 (12.0) | n.s. | CM | [68] |
| Horse (*Equus caballus*) | New Zealand | 2002 | O^c^ | Farmed | Foals | | 6 | 5 (83.3) | S | CM | [371] |
| Horse (*Equus caballus*) | Poland | n.s. | P | Stabled | 2 age groups | | 318 | 11 (3.5) | n.s. | CM | [372] |
| Horse (*Equus caballus*) | UK | 2002 | Pilot | Farmed | 3 age groups | | 52 | 2 (3.8) | A | IFA | [373] |
| Horse (*Equus caballus*) | Argentina | n.s. | O | n.s. | No age group | | 2 | 2 (100) | n.s. | CM | [88] |
| Horse (*Equus caballus*) | Iran | 2002-2003 | P | Householded | 2 age groups | | 212 | 35 (15.8) | A | CM | [374] |
| Horse (*Equus caballus*) | Tunisia | 2003-2004 | P | Farmed | 1-3 years old | | 190 | 00 (00.0) | n.s. | CM | [101] |

**Additional file 2: Table S2.** (*Continuted*)

| **Host (Scientific name)** | **Country** | **Period of study** | **Study**  **type** | **Keeping status** | **Age group(s)** | | **Sample size** | | **No. positive (%)** | **Sign^a^** | **Technique(s)** | **Reference Number** |
| --- | --- | --- | --- | --- | --- | --- | --- | --- | --- | --- | --- | --- |
| Horse (*Equus caballus*) | Brazil | 2007 | P | Stabled | No age group | | | 64 | 48 (75.0) | A | CM | [375] |
| Horse (*Equus caballus*) | New Zealand | 2005-2007 | O^d^ | n.s. | Foals | | | 67 | 12 (18.0) | S | CM | [376] |
| Horse (*Equus caballus*) | USA | 2005-2008 | O | n.s. | No age group | | | 11 | 00 (00.0) | n.s. | PCR | [134] |
| Horse (*Equus caballus*) | Brazil | 2006 | P | Farmed | 6 age groups | | | 396 | 3 (0.75) | n.s. | CM | [377] |
| Horse (*Equus caballus*) | Greece | 2006-2007 | P | Stabled | 4 age groups | | | 150 | 7 (4.7) | n.s. | CM | [378] |
| Horse (*Equus caballus*) | Greece | 2006-2007 | P | Grazed | 4 age groups | | | 73 | 9 (12.3) | n.s. | CM | [378] |
| Horse (*Equus caballus*) | Iran | 2002-2003 | P | Farmed | 3-7 years old | | | 200 | 50 (25.0) | A | CM | [379] |
| Horse (*Equus caballus*) | England & Wales | 2004-2006 | O | Farmed | No age group | | | 12 | 3 (25.0) | n.s. | IFA | [148] |
| Horse (*Equus caballus*) | Brazil | 2007 | P | n.s. | No age group | | | 63 | 00 (00.0) | n.s. | CM | [150] |
| Horse (*Equus caballus*) | Italy | 2007 | P | Farmed | 5 age groups | | | 150 | 15 (10.0) | A | IFA | [380] |
| Horse (*Equus caballus*) | Brazil | 2008 | P | Stabled | No age group | | | 90 | 25 (27.8) | n.s. | CM | [381] |
| Horse (*Equus caballus*) | USA | 2009 | P | Farmed | 2 age groups | | | 349 | 16 (4.6) | M | IFA | [382] |
| Horse (*Equus caballus*) | Iraq | 2007 | P | n.s. | 4-10 years old | | | 107 | 29 (27.1) | n.s. | CM | [383] |
| Horse (*Equus caballus*) | Brazil | 2010-2011 | P | Farmed | Foals | | | 98 | 21 (21.4) | n.s. | CM | [384] |
| Horse (*Equus caballus*) | Brazil | 2010-2011 | P | Farmed | Mares | | | 98 | 18 (18.4) | n.s. | CM | [384] |
| Horse (*Equus caballus*) | Algeria | 2010-2011 | P | Farmed | 3 age groups | | | 138 | 4 (2.9) | n.s. | PCR | [385] |
| Horse (*Equus caballus*) | Taiwan | 2001-2003 | P | Farmed | No age group | | | 436 | 161 (37.0) | n.s. | CM | [386] |
| Horse (*Equus caballus*) | Algeria | 2011-2013 | P | Farmed | 3 age groups | | | 219 | 5 (2.3) | n.s. | PCR | [365] |
| Horse (*Equus caballus*) | Czechia & Poland | 2011-2012 | P | Farmed | 5 days-32 years | | | 352 | 12 (3.4) | n.s. | PCR | [387] |
| Horse (*Equus caballus*) | Italy | 2012 | O^b^ | Hospitalized | 2 age groups | | | 73 | 14 (19.1) | A | PCR | [388] |
| Horse (*Equus caballus*) | China | 2013 | P | Pastured | 2 age groups | | | 262 | 7 (2.7) | n.s. | PCR | [389] |
| Horse (*Equus caballus*) | China | n.s. | O | n.s. | 2-7 years old | | | 29 | 2 (6.9) | S | PCR | [390] |
| Mustang (*Equus caballus*) | USA | n.s. | P | Wild | 2 age groups | | | 34 | 3 (8.8) | A | PCR | [391] |
| Pony (*Equus caballus*) | USA | n.s. | P | Wild | 2 age groups | | | 50 | 25 (50.0) | A | PCR | [391] |
| Quarterhorse (*Equus caballus*) | USA | n.s. | O | n.s. | 2.5 year old | | | 1 | 1 (100.0) | S | IFA | [392] |
| Zebra (*Equus zebra*) | Tanzania | 1995 | O | Wild | No age group | | | 25 | 7 (28.0) | n.s. | CM | [35] |
| Zebra (*Equus burchelli bohemi*) | Brazil | 2008 | O | Captive | No age group | | | 2 | 00 (00.0) | n.s. | CM | [3] |
| **Family Hippopotamidae** |  |  |  |  |  | | |  |  |  |  |  |
| Hippo (*Hippopotamus amphibious*) | Brazil | 2008 | O | Captive | No age group | | | 2 | 00 (00.0) | n.s. | CM | [3] |
| **Family R**[**hinocerotidae**](https://en.wikipedia.org/wiki/Rhinocerotidae) |  |  |  |  |  | | |  |  |  |  |  |
| Rhinoceros (*Ceratotherium simum*) | Spain | n.s. | O | Captive | No age group | | | 3 | 3 (100) | n.s. | CM | [34] |
| Rhinoceros (*Ceratotherium simum*) | Poland | n.s. | O | Captive | Adult | | | 1 | 1 (100) | n.s. | CM | [323] |
| Rhinoceros (*Ceratotherium simum*) | Brazil | 2008 | O | Captive | No age group | | | 2 | 00 (00.0) | n.s. | CM | [3] |
| **Family Giraffidae** |  |  |  |  |  | | |  |  |  |  |  |
| Giraffe (*Giraffa camelopardalis*) | Spain | n.s. | O | Captive | No age group | | | 3 | 1 (33.3) | n.s. | CM | [34] |
| Giraffe (*Giraffa c. reticulata*) | Czechia | n.s. | O | Captive | No age group | | | 25 | 1 (4.0) | n.s. | CM | [393] |
| Giraffe (*Giraffa c. rotschildi*) | Czechia | n.s. | O | Captive | No age group | | | 20 | 00 (00.0) | n.s. | CM | [393] |
| Giraffe (*Giraffa camelopardalis*) | Brazil | 2008 | O | Captive | No age group | | | 2 | 00 (00.0) | n.s. | CM | [3] |
| **Family Elephantidae** |  |  |  |  | |  | |  |  |  |  |  |
| Elephant (*Elephas maximus*) | Poland | n.s. | O | Captive | | Adult | | 1 | 1 (100) | n.s. | CM | [323] |
| Elephant (*Elephas maximus*) | Brazil | 2008 | O | Captive | | No age group | | 2 | 00 (00.0) | n.s. | CM | [3] |
| Elephant (*Loxodonta africana*) | South Africa | 2008 | O | Wild | | No age group | | 93 | 24 (25.8) | n.s. | CM | [36] |

n.s. = Not stated

CM = Conventional microscopy (e.g., Ziehl-Neelsen acid-fast or auramine staining method and microscopic examination)

ICT = Immunochromatographic test

QLAT = Quantitative latex agglutination

ELISA = Enzyme-linked immunosorbent assay

PCR = Polymerase chain reaction

IFA = Immunofluorescence assay

O = Other type of studies (non-prevalence study)

P = Prevalence study

^a^ Symptomatic defined as the present of diarrhoea

^b^ Two or more samples from each animal, and overall prevalence with at least one positive result identified (Longitudinal study)

^c^ Faecal samples from outbreaks of diarrhoeic animals

^d^ Studies based on results of analyses of faecal samples submitted to laboratories for rotine examination or postmortem examination and/or diarrhoeic animals admitted to the veterinary clinics

^e^ Case-control study

^f^ Dry season

^g^ Wet season

^h^ Samples collected from the ground

**References**

1. Heuschele WP, Oosterhuis J, Janssen D, Robinson PT, Ensley PK, Meier JE, et al. Cryptosporidial infections in captive wild animals. J Wildlife Dis. 1986;22:493-496.
2. Karanis P, Plutzer J, Abdul Halim N, Igori K, Nagasawa H, Ongerth J, et al. Molecular characterization of *Cryptosporidium* from animal sources in Qinghai province of China. Parasitol Res. 2007;101:1575-1580.
3. Ludwig R, Marques SMT. Occurrence of *Cryptosporidium* spp. oocysts in mammals at a zoo in southern Brazil. Rev Ibero-Latinoam Parasitol. 2011;70,122-128.
4. Heitman TL, Frederick LM, Viste JR, Guselle NJ, Morgan UM, Thompson RCA, et al. Prevalence of *Giardia* and *Cryptosporidium* and characterization of *Cryptosporidium* spp. isolated from wildlife, human, and agricultural sources in the North Saskatchewan River Basin in Alberta, Canada. Can J Microbiol. 2002;48:530-541.
5. Geurden T, Goossens E, Levecke B, Vercammen F, Vercruysse J, Claerebout E. Occurrence and Molecular Characterization of *Cryptosporidium* and *Giardia* in Captive Wild Ruminants in Belgium. J Zoo Wildl Med. 2009;40:126-130.
6. Paziewska A, Bednarska M, Niewęgłowski H, Karbowiak G, Bajer A. Distribution of *Cryptosporidium* and *Giardia* spp. in selected species of protected and game mammals from North-Eastern Poland. Ann Agric Environ Med. 2007;14:265-270.
7. Dubey JP, Fayer R, Rao JR. Cryptosporidial oocysts in faeces of water buffalo and zebu cattle in India. J Vet Parasitol. 1992;6:55-56.

# Ribeiro MG, Langoni H, Jerez JA, Leite DS, Ferreira F, Gennari SM. Identification of enteropathogenes from buffalo calves with and without diarrhoea in the Ribeira Valley, State of Sao Paulo, Brazil. Bra J Vet Res Anim Sci. 2000;37:159-165.

1. Singh BB, Sharma R, Kumar H, Banga HS, Aulakh RS, Gill JPS, Sharma JK. Prevalence of *Cryptosporidium* *parvum* infection in Punjab (India) and its association with diarrhea in neonatal dairy calves. Vet Parasitol. 2006;140:162-165.
2. Parsa A, Nouri M. Prevalence of *cryptosporidium muris* - like (*andersoni*) infection in cattle and buffalo slaughtered in Tabriz area abattoir. J Vet Med. 2007;1:49-55. (In Persian).
3. Rinaldi L, Musella V, Condoleo R, Saralli G, Veneziano V, Bruni G, et al.. *Giardia* and *Cryptosporidium* in water buffaloes (*Bubalus* *bubalis*). Parasitol Res. 2007;100:1113-1118.
4. Caccio SM, Rinaldi L, Cringoli G, Condoleo R, Pozio E. Molecular identification of *Cryptosporidium parvum* and *Giardia duodenalis* in the Italian water buffalo (*Bubalus* *bubalis*). Vet Parasitol. 2007;150:146-149.
5. El-Khodery S, Osman SA. Cryptosporidiosis in buffalo calves (*Bubalus* *bubalis*): Prevalence and potential risk factors. Trop Anim Hlth Prod. 2008;40:419-426.
6. Nasir A, Avais M, Khan M, Ahmad N. Prevalence of *Cryptosporidium* *parvum* infection in Lahore (Pakistan) and its association with diarrhea in dairy calves. Int J Agri Biol. 2009;11:221-224.
7. Bhat SA, Juyal PD, Singla LD. Prevalence of cryptosporidiosis in neonatal buffalo calves in Ludhiana district of Punjab, India Asian J Anim Vet Adv. 2012;7:512-520.
8. Heidarnegadi SM, Mohebah M, Maraghi SH, Babaei Z, Farnia S, Bairami A, et al. *Cryptosporidium* spp. infection in human and domestic animals. Iran J Parasitol. 2012;7:53-58.

# **Feng Y, Karna SR, Dearen TK, Singh DK, Adhikari LN, Shrestha A, et al. Common occurrence of a unique *Cryptosporidium ryanae* variant in zebu cattle and water buffaloes in the buffer zone of the Chitwan National Park, Nepal. Vet** **Parasitol. 2012;185:309-314.**

1. Abeywardena H, Jex AR, von Samson-Himmelstjerna G, Haydon SR, Stevens MA, Gasser RB. First molecular characterisation of *Cryptosporidium* and *Giardia* from *Bubalus bubalis* (water buffalo) in Victoria, Australia. Infect. Genet Evol. 2013;20:96-102.
2. Masood S, Maqbool A, Anjum AA, Rashid MI, Choudhary ZI. Prevalence of *Cryptosporidium* oocysts in bovine at different livestock farms by conventional microscopic and molecular techniques. J Anim Plant Sci. 2013;23:1588-1594.
3. Maurya PS, Rakesh RL, Pradeep B, Kumar S, Kunda K, Garg R, et al. Prevalence and risk factors associated with *Cryptosporidium* spp. infection in young domestic livestock in India. Trop Ani Health Prod. 2013;45:941-946.
4. Singla LD, Gupta MP, Singh H, Singh ST, Kaur P, Juyal PD. Antigen based diagnosis of *Cryptosporidium* parvum infection in cattle and buffalo faeces. Indian J Anim Sci. 2013;83:37-39.
5. Amer S, Zidan S, Feng Y, Adamu H, Li N, Xiao L. Identity and public health potential of *Cryptosporidium* spp. in water buffalo calves in Egypt. Vet Parasitol. 2013;191:123-127.

# Helmy AY, Krücken J, Nöckler K, Samson-Himmelstjerna G, Zessin KH. Molecular epidemiology of ***Cryptosporidium*** in livestock animals and humans in the Ismailia province of Egypt. Vet Parasitol. 2013;193:15-24.

# Mahfouz, M.E., Mira, N.N., Amer, S.,. Prevalence and genotyping of ***Cryptosporidium*** spp. in farm animals in Egypt. J Vet Med Sci. 2014;76:1569-1575.

# Morsy GH, Megeed KNA, Hammam AM, Seliem MME, Khalil AM, Aboelsoued D. Prevalence of *Cryptosporidium* infection in buffalo calves with special reference to urea and creatinine levels. Glob Vet. 2014;13:662-667.

1. Inpankaew T, Jiyipong T, Wongpanit K, Pinyopanuwat N, Chimnoi W, Kengradomkij C, et al. Molecular detection of *Cryptosporidium* spp. infections in water buffaloes from northeast Thailand. Trop Anim Health Prod. 2014;46:487-490.
2. Bahrami S, Alborzi AR, Molayan PH, Purbaram S, Mousavi B.. Prevalence of *Cryptosporidium* spp. infection and its association with diarrhea in buffalo calves in Khuzestan, Southwestern Province of Iran. Buffalo Bull. 2014:33:150-155
3. Abeywardena H, Jex AR, Koehler AV, Rajapakse RJ, Udayawarna K, Haydon SR, et al. First molecular characterization of *Cryptosporidium* and *Giardia* from bovines (*Bos taurus* and *Bubalus bubalis*) in Sri Lanka: unexpected absence of *C. parvum* from pre-weaned calves. Parasit vectors. 2014;7:75.
4. Aquino MCC, Widemer G, Zucatto AS, Viol MA, Inacio SV, Nakamura AA, et al. First molecular characterization of *Cryptosporidium* spp. infecting buffalo calves in Brazil. J Eukaryot Microbiol. 2015;62:657-661.
5. Radhy AM. Prevalence of *Rota virus*, *Escherichia coli O157:H7* and *Cryptosporidium spp*. in feces of buffalo in Babil governorate. AL-Qadisiya J Vet Med Sci. 2015;14:75-79.
6. Hingole AC, Gudewar JG, Pednekar RP, Gatne ML. Prevalence and molecular characterization of *Cryptosporidium* species in cattle and buffalo calves in Mumbai region of India. J Parasit Dis. 2016;41:1-6.
7. Ibrahim MA, Abdel-Ghany AE, Abdel-Latef GK, Abdel-Aziz SA, Aboelhadid SM. Epidemiology and public health significance of *Cryptosporidium* isolated from cattle, buffaloes, and humans in Egypt. Parasitol Res. 2016;115:2439-2448.

# Zahedi A, Phasey J, Boland T, Ryan U. First report of *Cryptosporidium* species in farmed and wild buffalo from the Northern Territory, Australia. Parasitol Res. 2016;115:1349-1353.

1. Gomez MS, Vila T, Feliu C, Montoliu I, Gracenea M, Fernandez J. A survey for *Cryptosporidium* spp. in mammals at the Barcelona zoo. Int J Parasit. 1996;26:1331-1333.

# Mtambo MM, Sebatwale JD, Kambarage DM, Muhairwa AP, Maeda GE, Kusiluka LJ, et al. Prevalence of *Cryptosporidium* spp. Oocysts in cattle and wildlife in Morogoro region, Tanzania. Prev Vet Med. 1997;31:185-190.

1. Abu Samra N, Jori F, Samie A, Thompson P. The prevalence of *Cryptosporidium* spp. oocysts in wild mammals in the Kruger National Park, South Africa. Vet Parasitol. 2011;175:155-159.
2. [Hogan JN](https://www.ncbi.nlm.nih.gov/pubmed/?term=Hogan JN%5BAuthor%5D&cauthor=true&cauthor_uid=24171566), [Miller WA](https://www.ncbi.nlm.nih.gov/pubmed/?term=Miller WA%5BAuthor%5D&cauthor=true&cauthor_uid=24171566), [Cranfield MR](https://www.ncbi.nlm.nih.gov/pubmed/?term=Cranfield MR%5BAuthor%5D&cauthor=true&cauthor_uid=24171566), [Ramer J](https://www.ncbi.nlm.nih.gov/pubmed/?term=Ramer J%5BAuthor%5D&cauthor=true&cauthor_uid=24171566), [Hassell J](https://www.ncbi.nlm.nih.gov/pubmed/?term=Hassell J%5BAuthor%5D&cauthor=true&cauthor_uid=24171566), [Noheri JB](https://www.ncbi.nlm.nih.gov/pubmed/?term=Noheri JB%5BAuthor%5D&cauthor=true&cauthor_uid=24171566), et al. *Giardia* in mountain gorillas (*Gorilla beringei beringei*), forest buffalo (*Syncerus caffer*), and domestic cattle in Volcanoes National Park, Rwanda. J Wildl Dis. 2014;50:21-30.

# Rahaman AS, Sanyal SC, AL-Mahmud KA, Sobhan A, Hossain KS, Anderson BC. Cryptosporidiosis in calves and their handlers in Bangladesh. Lancet 1984;2:221.

1. de Visser NAPC, Breukink HJ, van Zijderveld FG, Leeuw PW. Enteric infections in veal calves: a longitudinal study on four veal calf units. Vet Q. 1987;9:289-296.
2. Bellinzoni RC, Blackhall J, Terzolo HR, Moreira AR, Auza N, Mattion N, et al.. Microbiology of diarrhea in young beef and dairy calves in Argentina. Rev Argent Microbiol. 1990;22:130-136

# **Rhee JK, Seu YS, Park BK. Isolation and identification of *Cryptosporidium* from various animals in Korea. I. Prevalence of *Cryptosporidium* in various animals. Korean J Parasitol. 1991;29:139-148.**

1. Villacorta I, Ares-Mazas E, Lorenzo MJ. *Cryptosporidium* *parvum* in cattle, sheep and pigs in Galicia (N.W. Spain). Vet Parasitol. 1991;38:249-252.
2. Lorenzo MJ, Ares-Mazfis E, Villacorta I. Detection of oocysts and lgG antibodies to *Cryptosporidium parvum* in asymptomatic adult cattle. Vet Parasitol. 1993;47:9-15.
3. Kaminjolo JS, Adesiyun AA, Loregnard R., Kitson-Piggott W. Prevalence of *Cryptosporidium* oocysts in livestock in Trinidad and Tobago. Vet Parasitol. 1993;45:209-213.
4. Adesiyun AA, Kaminjolo JS. Prevalence and epidemiology of selected enteric infections of livestock in Trinidad. Vet Parasitol. 1994;19:151-165.
5. Garber LP, Salman MD, Hurd HS, Keefe T, Schlater JL. Potential risk factors for *Cryptosporidium* infection in dairy calves. J Am Vet Med Assoc. 1994;205:86-91
6. Scott CA, Smith HV, Mtambo MMA, Gibbs HA. An epidemiological study of *Cryptosporidium* *parvum* in two herds of adult beef cattle. Vet Parasitol. 1995;57:277-288.
7. Bukhari Z, Smith HV. Detection of *Cryptosporidium* *muris* oocysts in the faeces of adult dairy cattle in Scotland. Vet Rec. 1996;138:207-208.

# **Wee SH, Joo HD, Kang YB. Evaluation for detection of *Cryptosporidium* oocysts in diarrheal feces of calves. Korean J Parasitol. 1996;34:121-126**.

1. Quílez J, Sánchez-Acedo C, del Cacho E, Clavel A, Causapé AC. Prevalence of *Cryptosporidium* and *Giardia* infections in cattle in Aragón (northeastern Spain). Vet Parasitol. 1996;66:139-146.
2. Caver JA, Hill JE, Thompson SJ. Surveillance of Cryptosporidia in veterinary diagnostic laboratory. J Vet Diagn Invest. 1996;8:496-500.
3. Pena HFJ, Kasai N, Gennari SM. *Cryptosporidium muris* in dairy cattle in Brazil. Vet Parasitol. 1997;73:353-355.
4. Olson ME, Thorlakson CL, Deselliers L, Morck DW, McAllister TA. *Giardia* and *Cryptosporidiumin* in Canadian farm animals. Vet Parasitol. 1997;68:375-381.
5. Pérez E, Kummeling A, Janssen MMH, Jiménez C, Alvarado R, Caballero M, et al. Infectious agents associated with diarrhoea of calves in the canton of Tilarán, Costa Rica. Prev Vet Med. 1998;33:195-205.
6. Maldonado-Camargo S, Atwill ER, Saltijeral-Oaxaca JA, Herrera-Alonso LC. Prevalence of and risk factors for shedding of *Cryptosporidium parvum* in Holstein Freisian dairy calves in central Mexico. Prev Vet Med. 1998;36:95-107.

# Bednarska M, Bajer A, Sinski E. Calves as a potential reservoir of *Cryptosporidium* *parvum* and *Giardia* sp. Ann Agric Environ Med. 1998;5:135-138.

1. Emre Z, Alabay BM, Fidancõ H, Duzgun A, Cerci H.. Prevalence of *Cryptosporidium* spp. infection and its relation to other enteric pathogens (*Escherichia* *coli* K99 and rotavirus) in cattle in Ankara, Turkey. Turk J Vet Anim Sci. 1998;22:453-458.
2. De la Fuente R, Luzon M, Ruiz-Santa-Quiteria JA, Garcia A, Cid D, Orden JA, et al. *Cryptosporidium* and concurrent infections with other major enteropathogens in 1 to 30-day-old diarrheic dairy calves in central Spain. Vet Parasitol. 1999;80:179-185.

# ****Nussbaum DJ, Salord JR, Rimmele DD. Evaluation of quantitative latex agglutination for detection of *Cryptosporidium parvum*, *E. coli* K99, and rotavirus in calf feces. J Vet Diag Invest. 1999;11:314-318.****

1. Wade SE, Mohammed HO, Schaaf SL. Prevalence of *Giardia* sp., *Cryptosporidium* *parvum* and *Cryptosporidium* *muris* (*C. andersoni*) in 109 dairy herds in five counties of southeastern New York. Vet Parasitol. 2000;93:1-11.
2. Hoar B, Atwill ER, Farver TB. Estimating maximum possible environmental loading amounts of *Cryptosporidium* *parvum* attributable to adult beef cattle. Quant Microbiol. 2000;2:21-36.
3. Uga S, Matsuo J, Kono E, Kimura K, Inoue M, Rai SK, Ono K. Prevalence of *Cryptosporidium* *parvum* infection and pattern of oocyst shedding in calves in Japan. Vet Parasitol. 2000;94:27-32.
4. Fonseca I, Fazendeiro I, Antunes A. Genetic characterization of *Cryptuspoddiurn pawurn* isolates from cattle in Portugal: animal and human implications. J Eukaryot Microbiol. 2001;32S-33S.
5. Huetink RE, van der Giessen JW, Noordhuizen JP, Ploeger HW. Epidemiology of *Cryptosporidium* spp. and *Giardia duodenalis* on a dairy farm. Vet Parasitol. 2001;102:53-67.

# Misic Z, Katic-Radivojevic S, Kulisic Z. *Cryptosporidium* infections in calves aged up to three months. Acta Veterinaria 2001;51:143-148.

1. Enemark HL, Ahrens P, Lowery C, Thamsborg SM, Enemark JMD, Bille-Hansen V, Lind, P. *Cryptosporidium andersoni* from a danish cattle herd: identification and preliminary characterization. Vet Parasitol. 2002;107:37-49.
2. Pilarczyk B, Balicka-Ramisz A. Prevalence of *Cryptosporidium* sp. in western Pomerania. Elect. J. Polis Agricult Univ Anim Husbandry. 2002;5:1-8.
3. Mahdi NK, Ali NH. Cryptosporidiosis among animal handlers and their livestock in Basrah, Iraq. E Afr Med J. 2002;79:550-553.

# Misic Z, Katic-Radivojevic S, Kulisic Z. *Cryptosporidium* infections in weaners, bull calves and postparturient cows in the Belgrade area. Acta Veterinaria. 2002;52:37-42.

1. Bodley-Tickell AT, Kitchen SE, Sturdee AP. Occurrence of *Cryptosporidium* in agricultural surface waters during an annual farming cycle in lowland UK. Water Res. 2002;36:1880-1886.
2. Björkman C, Svensson C, Christensson B, De Verdier K. *Cryptosporidium* *parvum* and *Giardia intestinalis* in calf diarrhoea in Sweden. Acta Vet Scand. 2003;44:145-152.

# **Duffy G, McEvoy JM, Moriarty EM, Sheridan JJ. A study of *Cryptosporidium parvum* in beef. Teagasc Project Report RMIS 4723. Dublin: Teagasc, National Food Centre**. **2003.**

1. Learmonth JJ, Ionas G, Pita AB, Cowie RS. Identification and genetic characterisation of *Giardia* and *Cryptosporidium* strains in humans and dairy cattle in the Waikato Region of New Zealand. Water Sci Technol. 2003;47:21-26.
2. Atwill ER, Hoar B, das Gracas C, Pereira M, Tate KW, Rulofson F, et al. Improved quantitative estimates of low environmental loading and sporadic periparturient shedding of *Cryptosporidium parvum* in adult beef cattle. Appl Environ Microbiol. 2003;69:4604-4610.

# **Becher KA, Robertson ID, Fraser DM, Palmer DG, Thompson RCA. Molecular epidemiology of *Giardia* and *Cryptosporidium* infections in dairy calves originating from three sources in Western Australia. Vet Parasitol. 2004;123:1-9**.

# **Epe C, Coati N, Schnieder T. Results of parasitological examinations of faecal samples from horses, ruminants, pigs, dogs, cats, hedgehogs and rabbits between 1998 and 2002. Dtsch Tierarztl Wochenschr. 2004;111:243-247**.

# **Yu JR, Lee JK, Seo M, Kim SI, Sohn WM, Huh S, et al. Prevalence of cryptosporidiosis among the villagers and domestic animals in several rural areas of Korea. Korean J Parasitol. 2004;42:1-6.**

1. Santín M, Trout JM, Xiao L, Zhou L, Greiner E, Fayer R. Prevalence and age-related variation of *Cryptosporidium* species and genotypes in dairy calves. Vet Parasitol. 2004;122:103-117.
2. McAllister TA, Olson ME, Fletch, A, Wetzstein M, Entz T. Prevalence of *Giardia* and *Cryptosporidium* in beef cows in southern Ontario and in beef calves in southern British Columbia. Can Vet J. 2005;46:47-55.
3. Koyama Y, Satoh M, Meakawa K, Hilkosaka K, Nakai Y. Isolation of *Cryptosporidium* andersoni Kawatabi type in a slaughterhouse in the island of Japan. Vet Parasitol. 2005;130:323-326.
4. Trotz-Williams LA, Jarvie BD, Martin SW, Leslie KE, Peregrine AS. Prevalence of *Cryptosporidium* *parvum* infection in Southwestern Ontario and its association with diarrhea in neonatal dairy calves. Can Vet J. 2005;46:349-351.
5. Farizawati S, Lim YA, Ahmad RA, Fatimah CTNI, Siti-Nor Y. Contribution of cattle farms towards river contamination with *Giardia* cysts and *Cryptosporidium* oocysts in Sungai Langat Basin. Trop Biomed. 2005;22:89-98
6. Watanabe Y, Yang CH, Ooi HK. *Cryptosporidium* infection in livestock and first identification of *Cryptosporidium parvum* genotype in cattle feces in Taiwan. Parasitol Res. 2005;97:238-241.
7. Grinberg A, Pomroy WE, Weston JF, Ayanegui-Alcerreca A, Knight D. The occurrence of *Cryptosporidium parvum*, *Campylobacter* and *Salmonellain* newborn dairy calves in the Manawatu region of New Zealand. N Z Vet J. 2005;53:315-320.
8. Degerli S, Celiksoz A, Kalkan K, Ozcelik S. Prevalence of *Cryptosporidium* spp. and *Giardia* spp. in cows and calves in Sivas. Turk J Vet Anim Sci. 2005;29:995-999.
9. Kusiluka LJM, Karimuribo ED, Mdegela RH, Luogu EJ, Munishi PKT, Mlozi MRS, Kambarage DM. Prevalence and impact of water-borne zoonotic pathogens in water, cattle, and humans in selected village in Dodoma, rural and bagamoyo districts, Tanzania. Physics Chemistry Earth Parts A/B/C. 2005;30:818-825.

# Moriarty EM, McEvoy JM, Lowery CJ, Thompson HP, Finn M, Sheridan JJ, et al. Prevalence and characterisation of *Cryptosporidium* species in cattle faeces and on beef carcases at slaughter. Vet Rec. 2005;156:165-168.

1. Venturini L, Bacigalupe D, Basso W, Unzaga JM, Venturini MC, Moré G. *Cryptosporidium parvum* em animales domésticos y em monos de un zoológico. Parasitol Latinoam. 2006;61:90-93.
2. Goz Y, Atug N, Yuksek N, Ozkan C. Parasites detected in neo natal and young calves with diarrhea Bull. Vet Inst Pulawy. 2006;50:345-348.
3. Radfar MH, Molaei MM, Baghbannejad A. Prevalence of *Cryptosporidium* SPP. oocysts in dairy calves in Kerman, southeastern Iran. Iranian J Vet Res. 2006;7:81-84.
4. Gow S, Waldner C. An examination of the prevalence of and risk factors for shedding of *Cryptosporidium* spp. and Giardia spp. in cows and calves from western Canadian cow-calf herds. Vet Parasitol. 2006;137:50-61.
5. Kvac M, Kouba M, Vitovec J. Age-related and housing-dependence of *Cryptosporidium* infection of calves from dairy and beef herds in South Bohemia, Czech Republic. Vet Parasitol. 2006;137:202-209.
6. Maddox-Hyttel C, Langkjaer RB, Enemark HL, Vigre H. *Cryptosporidium* and *Giardia* in different age groups of Danish cattle and pigs-Occurrence and management associated risk factors. Vet Parasitol. 2006;14:48-59.
7. Roy SS, Sarkar S, Batabyal S, Pramanik AK, Das P. Observations on the epidemiology of bovine cryptosporidiosis in India. Vet Parasitol. 2006;141:330-333.
8. Fayer R, Santın M, Trout JM, Greiner E. Prevalence of species and genotypes of *Cryptosporidium* found in 1-2-year-old dairy cattle in the eastern United States. Vet Parasitol. 2006;135:105-112.
9. Haschek B, Klein D, Benetka V, Herrera C, Sommerfeld-Stur I, Vilcek Š, et al. Detection of bovine torovirus in neonatal calf diarrhoea in lower Austria and Styria (Austria). J Vet Med B. 2006;53:160-165.
10. Hamnes IS, Gjerde BK, Robertson LJ. Prevalence of *Giardia* and *Cryptosporidium* in dairy calves in three areas of Norway. Vet Parasitol. 2006;140:204-216.
11. Castro-Hermida JA, Carro-Corral C, González-Warleta M, Mezo M. Prevalence and intensity of infection of *Cryptosporidium* spp. and *Giardia* *duodenalis* in dairy cattle in Galicia (NW Spain). J Vet Med B. 2006;53:244-246.
12. Geurden T, Goma FY, Siwila J, Phiri IGK, Mwanza AM, Gabriel S, et al. Prevalence and genotyping of *Cryptosporidium* in three cattle husbandry systems in Zambia. Vet Parasitol. 2006;138:217-222.
13. Swai ES, French NP, Karimuribo ED, Fitzpatrick JL, Bryant MJ, Kambarage DM, et al. Prevalence and determinants of *Cryptosporidium* spp. infection in smallholder dairy cattle in Iringa and Tanga Regions of Tanzania. Onderstepoort J Vet Res. 2007;74:23-29.

# Soltane R, Guyot K, Dei-Cas E, Ayadi A. Prevalence of *Cryptosporidium* spp. (Eucoccidiorida: Cryptosporiidae) in seven species of farm animals in Tunisia. Parasite. 2007;14:335-338.

1. Geurden T, Berkvens D, Martens C, Casaert S, Vercruysse J, Claerebout E. Molecular epidemiology with subtype analysis of *Cryptosporidium* in calves in Belgium. Parasitology. 2007;134:1981-1987.
2. Thompson HP, Dooley JS, Kenny J, McCoy M, Lowery CJ, Moore JE. Genotypes and subtypes of *Cryptosporidium* spp. in neonatal calves in Northern Ireland. Parasitol Res. 2007;100:619-624.
3. Misic Z, Abe N. Subtype analysis of *Cryptosporidium parvum* isolates from calves on farms around Belgrade, Serbia and Montenegro, using the 60 kDa glycoprotein gene sequences. Parasitology 2007;134:351-358.
4. Trotz-Williams LA, Wayne Martin S, Leslie KE, Duffield T, Pereqrine AS. Calf-level risk factors for neonatal diarrhea and shedding of *Cryptosporidium parvum* in Ontario dairy calves. Prev Vet Med. 2007;82:12-28.
5. Azimi M. Prevalence of *Cryptosporidium* infection in cattle in Isfahan, Iran. J Eukaryot Microbiol. 2007;54:100-102.
6. Mendonca C, Almeida A, Castro A, Delgado ML, Soares S, Correia da Costa JM, Canada N. Molecular characterization of *Cryptosporidium* and *Giardia* isolates from cattle from Portugal. Vet Parasitol. 2007;147:47-50.
7. Coklin T, Farber J, Parrington L, Dixon B. Prevalence and molecular characterization of *Giardia duodenalis* and *Cryptosporidium* spp. in dairy cattle in Ontario, Canada. Vet Parasitol. 2007;150:297-305.
8. Fayer R, Santin M, Trout JM. Prevalence of *Cryptosporidium* species and genotypes in mature dairy cattle on farms in eastern United States compared with younger cattle from the same locations. Vet Parasitol. 2007;145:260-266.
9. Castro-Hermida JA, Almeida A, González-Warleta M, Correia da Costa JM, Rumbo-Lorenzo C, Mezo M. Occurrence of *Cryptosporidium parvum* and *Giardia duodenalis* in healthy adult domestic ruminants. Parasitol Res. 2007;101:1443-1448.
10. Plutzer J, Karanis P. Genotype and subtype analyses of *Cryptosporidium* isolates from cattle in Hungary, Vet Parasitol. 2007;146:357-362.
11. Pirestani M, Sadraei J, Dalimi A, Zawar M, Vaeznia H. Molecular characterization of *Cryptosporidium* isolates from human and bovine using 18S rRNA gene in Shahriar county of Tehran, Iran. Parasitol Res. 2008;103:447-467.
12. Fotouhi Ardakani R, Fasihi Harandi M, Soleiman Banaei S, Kamyabi H, Atapour M, Sharifi I. Epidemiology of *Cryptosporidium* infection of cattle in Kerman/Iran and molecular genotyping of some isolates, J Kerman Uni Med Sci. 2008;15:313-320.
13. Brook EJ, Hart CA, French NP, Christley RM. Molecular epidemiology of *Cryptosporidium* subtypes in cattle in England. Vet J. 2009;179:378-382.
14. Trotz Williams LA, Martin SW, Leslie KE, Duffield T, Nydam DV, Pereqrine AS. Association between management practices and within-herd prevalence of *Cryptosporidium parvum* shedding on dairy farms in southern Ontario. Prev Vet Med. 2008;83:11-23.
15. Uhde FL, Kaufmann T, Sager H, Albini S, Zanoni R, Schelling E, Meylan M. Prevalence of four enteropathogens in the faeces of young diarrhoeic dairy calves in Switzerland. Vet Rec. 2008;163:362-366.
16. Gul A, Cicek M, Kilinic O. Prevalence of *Eimeria* spp., *Cryptosporidium* spp. and *Giardia* spp. in calves in the Van province. Trukiye Parazitol Derg. 2008;3:202-20.
17. Szonyi B, Kang’ethe EK, Mbae CK, Kakundi EM, Kamwati SK, Mohammed HO. First report of *Cryptosporidium* deer-like genotype in Kenyan cattle, Vet Parasitol. 2008;153:172-175.
18. Cicek M, Korkoca H, Gul A. Investigation of *Cryptosporidium* sp. in workers of the Van Municipality slaughterhouse and in slaughtered animals. Trukiye Parazitol Derg. 2008;32:8-11.
19. Paul S, Chandra D, Ray DD, Tewari AK, Rao JR, Banerjee PS, Baidya S, Raina OK. Prevalence and molecular characterization of bovine *Cryptosporidium* isolates in India. Vet Parasitol. 2008;153:143-146.
20. Burenbaatar B, Bakheit M, Plutzer J, Suzuki N, Igarashi I, Ongerth J, Karanis P. Prevalence and genotyping of *Cryptosporidium* species from farm animals in Mongolia. Parasitol Res. 2008;102:901-905.
21. Quilez J, Torres E, Chalmers RM, Robinson G, Del Cacho E, Sanchez-Acedo C. *Cryptosporidium* species and subtype analysis from dairy calves in Spain. Parasitology 2008;135:1613-1620.
22. Nuchjangreed C, Boonrod K, Ongerth J, Karanis P. Prevalence and molecular characterization of human and bovine *Cryptosporidium* isolates in Thailand. Parasitol Res. 2008;103:1347-1353.
23. Sari B, Aktas MS, Arslan MO. The Prevalence of *Cryptosporidium* spp. in calves in Erzurum Province. Trukiye Parazitol Derg. 2008;3:116-119.
24. Del Coco VF, Cordoba MA, Basualdo JA. *Cryptosporidium* infection in calves from a rural area of Buenos Aires, Argentina. Vet Parasitol. 2008;158:31-35.
25. Halim NA, Plutzer J, Bakheit MA, Karanis P. First report of *Cryptosporidium* deer-like genotype in Malaysian cattle. Vet Parasitol. 2008;152:325-329.
26. Moriarty EM, Sinton LW, Mackenzie ML, Karki N, Wood DR. A survey of enteric bacteria and protozoans in fresh bovine faeces on New Zealand dairy farms. J Appl Microbiol. 2008;105:2015-2025.
27. Feltus DC, Giddings CW, Khaitsa ML, McEvoy JM. High prevalence of *Cryptosporidium bovis* and the deer-like genotype in calves compared to mature cows in beef cow-calf operations. Vet Parasitol. 2008;151:191-195.
28. Winkworth CL, Matthaei CD, Townsend CR. Prevalence of *Giardia* and *Cryptosporidium* spp. in calves from a region in New Zealand experiencing intensification of dairying. N Z Vet J. 2008;56:15-20
29. Duranti A, Caccio SM, Pozio E, Di Egidio A, De Curtis M, Battisti A, Scaramozzino P. Risk factors associated with *Cryptosporidium parvum* infection in cattle. Zoonoses Public Health. 2009;56:176-182.
30. Banda Z, Nichols RAB, Grimason AM, Smith HV. *Cryptosporidium* infection in non-human hosts in Malawi. Onderstepoort J Vet Res. 2009;76:363-375.
31. Nolan MJ, Jex AR, Mansell PD, Browning GF, Gasser RB. Genetic characterization of *Cryptosporidium parvum* from calves by mutation scanning and targeted sequencing-zoonotic implications. Electrophoresis 2009;30:2640-2647.
32. Silverlas C, Emanuelson U, de Verdier K, Björkman C. Prevalence and associated management factors of *Cryptosporidium* shedding in 50 Swedish dairy herds. Prev Vet Med. 2009;90:242-253.
33. Jellison KL, Lynch AE, Ziemann JM. Source tracking identifies deer and geese as vectors of human infectious *Cryptosporidium* genotypes in an urban/suburban watershed. Environ Sci Technol. 2009;43:4267-4272.
34. Coklin T, Uehlinger FD, Farber JM, Barkema HW, O’Handley RM, Dixon B. Prevalence and molecular characterization of *Cryptosporidium* spp. in dairy calves in 11 farms in Prince Edward Island, Canada. Vet Parasitol. 2009;160:323-326.
35. Klein D, Kern A, Lapan G, Benetka V, Möstl K, Hassl A, Baumgartner W. Evaluation of rapid assays for the detection of bovine coronavirus, rotavirus A and *Cryptosporidium parvum* in faecal samples of calves. Vet J. 2009;182:484-486.
36. Keshavarz A, Haghighi A, Athari A, Kazemi B, Abadi A, Mojarad EN. Prevalence and molecular characterization of bovine *Cryptosporidium* in Qazvin province, Iran. Vet Parasitol. 2009;160:316-318.
37. Paul S, Chandra D, Tewari AK, Banerjee PS, Ray DD, Raina OK, Rao JR. Prevalence of *Cryptosporidium andersoni*: A molecular epidemiological survey among cattle in India. Vet Parasitol. 2009;161:31-35.
38. Mallinath RHK, Chikkachowdappa PG, Gowda. AJK, D’souza PE. Studies on the prevalence of cryptosporidiosis in bovines in organized dairy farms in and around Bangalore, South India. Vet Archiv. 2009;79:461-470.
39. Amer S, Honma H, Ikarashi M, Oishi R, Endo M, Otawa K, Nakai Y. The first detection of *Cryptosporidium* deer-like genotype in cattle in Japan. Parasitol Res. 2009;104:745-752.
40. Castro-Hermida JA, Garcia-Presedo I, Almeida A, Gonzalez-Warleta M, Correia Da Costa JM, Mezo M. Detection of *Cryptosporidium* spp. and *Giardia duodenalis* in surface water: a health risk for humans and animals. Water Res. 2009;43:4133-4142
41. Ondrackova Z, Kvac M, Sak B, Kvetonova D, Rost M. Prevalence and molecular characterization of *Cryptosporidium* spp. in dairy cattle in South Bohemia, the Czech Republic. Vet Parasitol. 2009;165:141-144
42. Liu AQ, Wang RJ, Li YH, Zhang LX, Shu J, Zhang WZ, Feng YY, Xiao LH, Ling H. Prevalence and distribution of *Cryptosporidium* spp. in dairy cattle in Heilongjiang Province, China. Parasitol Res. 2009;105:797-802.
43. Pilarczyk B, Balicka-Ramisz A, Kozak W, Ramisz A. Occurrence of endoparasites in heifers imported to Poland from the Netherlands. Archiv Tierzucht. 2009;52:265-271.
44. Pilarczyk B, Kolodziejczyk L, Zaja̧czkowska K, Kuzna-Grygiel W, Balicka-Ramisa A, Tomza-Marciniak A, Pilarczyk R,. Prevalence of *Eimeria* and *Cryptosporidium* sp. protozoa in polish cows and in cows imported from the Netherlands as in-calf heifers. Bull Vet Inst Pulawy. 2009;53:637-640.
45. Ralston B, Thompson RC, Pethick D, McAllister TA, Olson ME. *Cryptosporidium* *andersoni* in Western Australian feedlot cattle. Aust Vet J. 2010;88:458-460.
46. Swai ES, Schoonman L. Investigation into the prevalence of *Cryptosporidium* infection in calves among small-holder dairy and traditional herds in Tanzania. Vet Med Int. 2010;676451.
47. Smith RP, Chalmers RM, Mueller-Doblies D, Clifton-Hadley FA, Elwin K, Watkins J, Paiba GA, Hadfield SJ, Giles M. Investigation of farms linked to human patients with cryptosporidiosis in England and Wales. Prev Vet Med. 2010;94:9-17.
48. Silverlas C, Näslund K, Björkman C, Mattson JG. Molecular characterisation of *Cryptosporidium* isolates from Swedish dairy cattle in relation to age, diarrhoea and region. Vet Parasitol. 2010;169:289-295.
49. Sevá AP, Funada MR, Souza SO, Nava A, Richtzenhain LJ, Soares RM. Occurrence and molecular characterization of *Cryptosporidium* spp. isolated from domestic animals in a rural area surrounding Atlantic dry forest fragments in Teodoro Sampaio municipality, State of São Paulo, Brazil. Rev Bras Parasitol Vet. 2010;19:249-253.
50. Bartels CJ, Holzhauer M, Jorritsma R, Swart WA, Lam TJ. Prevalence, prediction and risk factors of enteropathogens in normal and non-normal faeces of young Dutch dairy calves. Prev Vet Med. 2010;93:162-169.
51. Karanis P, Eiji T, Palomino L, Boonrod K, Plutzer J, Ongerth J, Igarashi I. First description of *Cryptosporidium bovis* in Japan and diagnosis and genotyping of *Cryptosporidium* spp. in diarrheic pre-weaned calves in Hokkaido. Vet. Parasitol. 2010;169:387-390.
52. Fayer R, Santín M, Dargatz D. Species of *Cryptosporidium* detected in weaned cattle on cow-calf operations in the United States. Vet Parasitol. 2010;170:187-192.
53. Featherstone CA, Giles M, Marshall JA, Mawhinney IC, Holliman A, Pritchard GC*.* *Cryptosporidium* species in calves submitted for post-mortem examination in England and Wales. Vet Rec. 2010;167:979-980.
54. Khan SM, Debnath C, Pramanik AK, Xiao L, Nozaki T, Ganguly S. Molecular characterization and assessment of zoonotic transmission of *Cryptosporidium* from dairy cattle in West Bengal, India. Vet Parasitol. 2010;171:41-47.
55. [Díaz P](https://www.ncbi.nlm.nih.gov/pubmed/?term=Díaz P%5BAuthor%5D&cauthor=true&cauthor_uid=20380767), [Quílez J](https://www.ncbi.nlm.nih.gov/pubmed/?term=Quílez J%5BAuthor%5D&cauthor=true&cauthor_uid=20380767), [Chalmers RM](https://www.ncbi.nlm.nih.gov/pubmed/?term=Chalmers RM%5BAuthor%5D&cauthor=true&cauthor_uid=20380767), [Panadero R](https://www.ncbi.nlm.nih.gov/pubmed/?term=Panadero R%5BAuthor%5D&cauthor=true&cauthor_uid=20380767), [López C](https://www.ncbi.nlm.nih.gov/pubmed/?term=López C%5BAuthor%5D&cauthor=true&cauthor_uid=20380767), [Sánchez-Acedo C](https://www.ncbi.nlm.nih.gov/pubmed/?term=Sánchez-Acedo C%5BAuthor%5D&cauthor=true&cauthor_uid=20380767). Genotype and subtype analysis of *Cryptosporidium* isolates from calves and lambs in Galicia (NW Spain). Parasitol. 2010;137(8):1187-93
56. Ayinmode AB, Fagbemi BO, Xiao L. Molecular characterization of *Cryptosporidium* spp. in native calves in Nigeria. Parasitol Res. 2010;107:1019-1021.
57. Ayinmode AB, Fagbemi BO. Prevalence of *Cryptosporidium* infection in cattle from South Western Nigeria. Veterinarski Arhiv. 2010;80:723-731
58. Inpankaew T, Jiyipong T, Pinyopanuwat N, Chimnoi W, Thompson, R.C., Jittapalapong S. Prevalence and genotyping of *Cryptosporidium* SPP from dairy cow fecal samples in western Thailand. Southeast Asian J Trop Med Public Health 2010;41:770-775.
59. Szonyi B, Bordonaro R, Wade SE, Mohammed HO. Seasonal variation in the prevalence and molecular epidemiology of Cryptosporidium infection in dairy cattle in the New York City Watershed. Parasitol Res. 2010;107:317-325.
60. Ng J, Yang R, McCarthy S, Gordon C, Hijjawi N, Ryan U. Molecular characterization of *Cryptosporidium* and *Giardia* in pre-weaned calves in Western Australia and New South Wales. Vet Parasitol. 2011;176:145-150.
61. Wang R, Ma G, Zhao J, Lu Q, Wang H, Zhang L, Jian F, Ning C, Xiao L. *Cryptosporidium andersoni* is the predominant species in post-weaned and adult dairy cattle in China. Parasitol Int. 2011;60:1-4.
62. Follet J, Guyot K, Leruste H, Follet-Dumoulin A, Hammouma-Ghelboun O, Certad G, Dei-Cas E, Halama P. *Cryptosporidium* infection in a veal calf cohort in France: molecular characterization of species in a longitudinal study. Vet Res. 2011;42:116.
63. Izzo MM, Kirkland PD, Mohler VL, Perkins NR, Gunn AA, House JK. Prevalence of major enteric pathogens in Australian dairy calves with diarrhoea. Aust Vet J. 2011;89:167-173.
64. Meireles MV, Oliveira FP, Teixeira WF, Coelho WM, Mendes LC. Molecular characterization of *Cryptosporidium* spp. in dairy calves from the state of São Paulo, Brazil. Parasitol Res. 2011;109:949-951.
65. Díaz-Lee A, Mercado R, Onuoha E, Ozaki L, Muñoz P, Muñoz V, et al. *Cryptosporidium parvum* in diarrheic calves detected by microscopy and identified by immunochromatographic and molecular methods. Vet Parasitol. 2011;176:139-144.
66. Tiranti K, Larriestra A, Vissio C, Picco N, Alustiza F, Degioanni A, Vivas A. Prevalence of *Cryptosporidium* spp. and *Giardia* spp., spatial clustering and patterns of shedding in dairy calves from Córdoba, Argentina. Rev Bras Parasitol Vet. 2011;20:140-147.
67. Afshari Safavi E, Reza Mohammadi GH, Naghibi A, Rad M. Prevalence of *Cryptosporidium* spp. infection in some dairy Herds of Mashhad (Iran) and its association with diarrhea in newborn calves. Comp Clin Pathol. 2011;20:103-107.
68. Wang R, Wang H, Sun Y, Zhang L, Jian F, Qi M, Ning C, Xiao L. Characteristics of *Cryptosporidium* transmission in preweaned dairy cattle in Henan, China. J Clin Microbiol*.* 2011;49:1077-1082.
69. Kvac M, Hromadova N, Kvetooova D, Rost M, Sak B. Molecular characterization of *Cryptosporidium* spp. in pre-weaned dairy calves in the Czech Republic: absence of *C. ryanae* and management-associated distribution of *C. andersoni*, *C. bovis* and *C. parvum* subtypes. Vet Parasitol. 2011;177:378-382.
70. Muhid A, Robertson I, Ng J, Ryan U. Prevalence of and management factors contributing to *Cryptosporidium* sp. infection in preweaned and post-weaned calves in Johor, Malaysia. Exp Parasitol. 2011;127:534-538.
71. Maikai BV, Umoh JU, Kwaga JKB, Lawal I, Maikai VA, Camae V, Xiao L. Molecular characterization of *Cryptosporidium* spp. in native breeds of cattle in Kaduna State, Nigeria. Vet Parasitol. 2011;178:241-245.
72. Cardona GA, Carabin H, Goni P, Arriola L, Robinson G, Fernández-Crespo JC. Identification and molecular characterization of *Cryptosporidium* and *Giardia* in children and cattle populations from the province of Alava, North of Spain. Sci Total Environ. 2011;412-413:101-108.
73. Waldron LS, Dimeski B, Beggs PJ, Ferrari BC, Power ML. Molecular epidemiology, spatiotemporal analysis, and ecology of sporadic human cryptosporidiosis in Australia. App Environ Microbiol. 2011;77:7757-7765.
74. Imre K, Lobo ML, Matos O, Popescu C, Genchi C, Dărăbus G. Molecular characterization of *Cryptosporidium* isolates from pre-weaned calves in Romania: is there an actual risk of zoonotic infections? Vet Parasitol. 2011;181:321-324.
75. Cano-Romero P, Alonso-Díaz MA, Figueroa-Castillo JA, Trigo-Tavera JF. Prevalence and incidence of *Cryptosporidium* spp. in calves from the central region of Veracruz, Mexico. Trop Subtrop Agroecosyst. 2011;13:567-571.
76. Budu-Amoako E, Greenwood SJ, Dixon BR, Barkema HW, McClure JT. *Giardia* and *Cryptosporidium* on dairy farms and the role these farms may play in contaminating water Sources in Prince Edward Island, Canada. J Vet Intern Med. 2012;26:668-673.
77. Budu-Amoako E, Greenwood SJ, Dixon BR, Barkema H.W, McClure JT. Occurrence of *Cryptosporidium* and *Giardia* on beef farms and water sources within the vicinity of the farms on Prince Edward Island, Canada. Vet Parasitol. 2012;184:1-9.
78. Kang'ethe EK, Mulinge EK, Skilton RA, Njahira M, Monda JG, Nyongesa C, et al. *Cryptosporidium* species detected in calves and cattle in Dagoretti, Nairobi, Kenya. Trop Anim Health Prod. 2012;44(Suppl 1):S25-31.
79. Chen F, Huang K. Prevalence and molecular characterization of *Cryptosporidium* spp. in dairy cattle from farms in China. J Vet Sci. 2012;13:15-22.
80. Oates SC, Miller MA, Hardin D, Conrad PA, Melli A, Jessup DA, Dominik C, Roug A, Tinker MT, Miller WA. Prevalence, Environmental Loading, and Molecular Characterization of *Cryptosporidium* and *Giardia* Isolates from Domestic and Wild Animals along the Central California Coast. App Environ Microbiol. 2012;78:8762-8772.
81. Ng J, Eastwood K, Walker B, Durrheim DN, Massey PD, Porgineaux P, et al. Evidence of *Cryptosporidium* transmission between cattle and humans in northern New South Wales. Exp Parasitol. 2012;130:437-441.
82. Delafosse A, Chartier C, Dupuy MC, Dumoulin M, Pors I, Paraud, C. *Cryptosporidium parvum* infection and associated risk factors in dairy calves in western France. Prev Vet Med. 2015;118:406-412.
83. Nguyen ST, Fukuda Y, Tada C, Sato R, Duong B, Nguyen DT, et al. Molecular characterization of *Cryptosporidium* in native beef calves in central Vietnam. Parasitol Res. 2012;111:1817-1820.
84. Silva FM, Lopes RS, Araújo-Junior JP. Identification of *Cryptosporidium* species and genotypes in dairy cattle in Brazil. Rev Bras Parasitol Vet. 2013;22:22-28.
85. Wegayehu T, Adamu H, Petros B. Prevalence of *Giardia duodenalis* and *Cryptosporidium* species infections among children and cattle in North Shewa Zone, Ethiopia. BMC Infect Dis. 2013;13:419-426.
86. Squire SA, Beyuo J, Amafu-Dey H. Prevalence of *Cryptosporidium* oocysts in cattle from Southern Ghana. Veterinarski Arhiv. 2013;83:497-507
87. Rzeżutka A, Kaupke A. Occurrence and molecular identification of *Cryptosporidium* species isolated from cattle in Poland. Vet Parasitol. 2013;196:301-306.
88. Venu R, Latha BR, Basith SA, Sreekumar C, Raj GD, Raman M. Comparative evaluation of conventional diagnostic methods for detection of Bovine cryptosporidiosis. Indian J Anim Sci. 2013;83:124-128.
89. Bhat SA, Juyal PD, Singh NK, Singla LD. Coprological investigation on neonatal bovine cryptosporidiosis in Ludhiana, Punjab. J Parasit Dis. 2013;37:114-117.
90. Silverlas C, Blanco-Penedo I. *Cryptosporidium* spp. in calves and cows from organic and conventional dairy herds. Epidemiol Infect. 2013;141:529-539.
91. Zhang W, Wang R, Yang F, Zhang L, Cao J, Zhang X. et al. Distribution and genetic characterizations of *Cryptosporidium* spp. in pre-weaned dairy calves in Northeastern China’s Heilongjiang Province. PLoS ONE 2013;8:e54857.
92. Amer S, Zidan S, Adamu H, Ye J, Roellig D, Xiao L, Feng Y. Prevalence and characterization of *Cryptosporidium* spp. in dairy cattle in Nile River delta provinces, Egypt. Exp Parasitol. 2013;135:518-523.
93. Regassa A, Gizaw O, Abunna F, Abebe R, Beyene D, Megersa B, et al. *Cryptosporidium* in calves, lambs and kids at Haramaya, eastern Ethiopia. Ethiop Vet J. 2013;17:81-94.
94. Zhao GH, Ren WX, Gao M, Bian QQ, Hu B, Cong MM, et al. Genotyping *Cryptosporidium andersoni* in Cattle in Shaanxi Province, Northwestern China. PLoS One 2013;8:e60112.
95. Jafari R, Maghsood AH, Fallah M. Prevalence of *Cryptosporidium* infection among livestock and humans in contact with livestock in Hamadan district, Iran, 2012. J Res Health Sci. 2013;13:86-89.
96. Murakoshi F, Tozawa Y, Inomata A, Horimoto T, Wada Y, Kato K. Molecular characterization of *Cryptosporidium* isolates from calves in Ishikari District, Hokkaido, Japan. J Vet Med Sci. 2013;75:837-840.
97. Pam VA, Dakul DA, Karshima NS, Bata SI, Ogbu KI, Daniel LN, et al. Survey of *Cryptosporidium* species among ruminants in Jos, Plateau State, North-Central Nigeria. J Vet Adv. 2013;3:49-54.
98. Ferreira Feitosa T, Longo Riberio Vilela V, Rudrigues Athayde AC. First report of *Cryptosporidium* spp. and *Giardia* duodenalis in calves from northeastern Brazil. Turk J Vet Anim Sci. 2013;37:743-746.
99. Asadpour M, Razmi G, Mohammadi G, Naghibi A. Prevalence and molecular identification of *Cryptosporidium* spp. In pre-weaned dairy calves in Mashhad area, khorasan razavi province, Iran. Iran J Parasitol**.** 2013;8:601-607
100. Mirzai Y, Yakhchali M, Mardani K. *Cryptosporidium parvum* and *Cryptosporidium andersoni* infection in naturally infected cattle of northwest Iran. Vet Res Forum. 2014;5:55-60.
101. Bawm S, Kyi S, Lay KK, Htun LL, Myaing TT. Prevalence and associated risk factors of *Cryptosporidium* and *Giardia* species in cattle within Mandalay region, Myanmar. The J Adv Parasitol. 2014;1:49-53.
102. Faleke OO, Yabo YA, Olaleye AO, Dabai YU, Ibitoye EB. Point prevalence of *Cryptosporidium* oocysts in calves grazing along river Rima bank in Sokoto, Nigeria, Pakistan J Biol Sci. 2014;17:443-446.
103. Huang J, Yue D, Meng Q, Wang R, Zhao J, Li J, et al. Prevalence and molecular characterization of *Cryptosporidium* spp. and *Giardia duodenalis* in dairy cattle in Ningxia, northwestern China. BMC Vet Res. 2014;10:1-5.
104. Gillhuber J, Rügamer D, Pfister K, Scheuerle M. Giardiosis and other enteropathogenic infections: a study on diarrhoeic calves in Southern Germany. BMC Res Notes 2014;7:112-120.
105. Zhao W, Wang R, Zhang W, Liu A, Cao J, Shen Y, Yang F, Zhang L. MLST subtypes and population genetic structure of *Cryptosporidium andersoni* from dairy cattle and beef cattle in northeastern China's Heilongjiang Province. PLoS ONE 2014;9:e102006.
106. Khair A, Alam MM, Rahman AKMA, Shahiduzzaman M. Prevalence of cryptosporidiosis in crossbred calves in two selected areas of Bangladesh. Bangladesh J Vet Med Assoc. 2014;12:185-190.
107. Cui Z, Wang R, Huang J, Wang H, Zhao J, Luo N, et al. Cryptosporidiosis caused by *Cryptosporidium parvum* subtype IIdA15G1 at a dairy farm in Northwestern China. Parasit Vectors 2014;7:529.
108. Mahami M, Fallah E, Ahmadi M, Safaiyan A, Bakhtiyari S, Naserifar R, et al. Molecular and parasitological study of *Cryptosporidium* isolates from cattle in Ilam, west of Iran. Iran J Parasitol. 2014;9:435-40.
109. Huang CC, Wang LC, Pan CH, Yang CH, Lai CH. Investigation of gastrointestinal parasites of dairy cattle around Taiwan. J Microbiol Immunol Infect. 2014;47:70-74.
110. Bodager JR, Parsons MB, Wright PC, Rasambainarivo F, Roellig D, Xiao L, et al. Complex epidemiology and zoonotic potential for *Cryptosporidium suis* in rural Madagascar. Vet Parasitol. 2015;207:140e143.
111. Al-Mawly JA, Grinberg A, Velathanthiri N, French N. Cross sectional study of prevalence, genetic diversity and zoonoticpotential of *Cryptosporidium parvum* cycling in New Zealand dairy farms. Parasit Vectors 2015;8:1-7.
112. Akinkuotu OA, Oluwasile BB, Jacobs EB, Adeyanju J, Okwelum N. Occurrence of *Cryptosporidium* coproantigens in diarrhoeic ruminants and Pigs in Ogun State, Southwest Nigeria. J Vet Adv*.* 2016;5:1127-1132.
113. Qi MZ, Fang YQ, Wang XT, Zhang LX, Wang RJ, Du SZ, et al. Molecular characterization of *Cryptosporidium* spp. in pre-weaned calves in Shaanxi Province north-western China. J Med Microbiol. 2015;64:111-116.
114. Bjorkman C, Lindstrom L, Oweston C, Ahola H, Troel K, Axen C. *Cryptosporidium* infections in suckler herd beef calves. Parasitol. 2015;142:1108-1114.
115. Shafieyan H, Alborzi A, Hamidinejat H, Tabandeh MR., Hajikolaei MRH. Prevalence of *Cryptosporidium* spp. in ruminants of Lorestan province, Iran. J Parasit Dis. 2016;40:1165-1169.
116. Aita J, Ichikawa-Seki M, Kinami A, Yaita S, Kumagai Y, Nishikawa Y, et al. Molecular characterization of *Cryptosporidium parvum* detected in Japanese black and Holstein calves in Iwate Prefecture and Tanegashima Island, Kagoshima Prefecture, Japan. J Vet Med Sci. 2015;77:997-999.
117. Aita J, Ichikawa-Seki M, Fukumoto M, Asada M, Kumagai Y, Nishikawa Y, Itagaki T. Molecular characterization of *Cryptosporidium* *andersoni* isolated Japanese black calves in Tokachi district, Hokkaido prefecture, Japan. J Protozool Res. 2015;25:44-47.
118. Adamu SG, Adamu NB, Aliyu AU, Atsanda NN, Mustapha FB, Muhammad YA, et al. Prevalence of *Cryptosporidium* Infection in Cattle in Maiduguri, North Eastern Nigeria. Bangl. J. Vet. Med. 2015;13:25-28.
119. Danladi YK, Ugbomoiko US. Epidemiology of cryptosporidiosis in ruminant Species in Kebbi State, Nigeria. IOSR J Agri Vet Sci. 2015;8:39-44.
120. Abu Samra N, Jori F, Cacciò SM, Frean J, Poonsamy B, Thompson PN. *Cryptosporidium* genotypes in children and calves living at the wildlife or livestock interface of the Kruger National Park, South Africa. Onderstepoort J Vet Res. 2015;83:a1024.
121. Cardona GA, de Lucio A, Bailo B, Cano L, de Fuentes I, Carmena D. Unexpected finding of feline-specific *Giardia* *duodenalis* assemblage F and *Cryptosporidium* *felis* in asymptomatic adult cattle in Northern Spain. Vet Parasitol. 2015;30:258-263.
122. Garro CJ, Morici GE, Utgés ME, Tomazic ML, Schnittger L. Prevalence and risk factors for shedding of *Cryptosporidium* spp. oocysts in dairy calves of Buenos Aires Province, Argentina. Parasite Epidemiol Control 2016;1:36-41.
123. Li F, Wang H, Zhang Z, Li J, Wang C, Zhao J, et al. Prevalence and molecular characterization of *Cryptosporidium* spp. and *Giardia duodenalis* in dairy cattle in Beijing. China. Vet Parasitol. 2016;219:61-65.
124. Wegayehu T, Karim R, Anberber M, Adamu H, Erko B, Zhang L, Tilahun G. Prevalence and genetic characterization of *Cryptosporidium* species in dairy calves in Central Ethiopia. PLoS One 2016;11:e0154647.
125. Hisamuddin NH, Hashim N, Soffian SN, Amin MHM, Wahab RA, Mohammad M, et al. Identification of *Cryptosporidium* from dairy cattle in Pahang, Malaysia. Korean J Parasitol. 2016;54:197-200.
126. Thomson S, Jonsson N, Innes EA, Katzer F. A multiplex PCR test to identify four common cattle adapted *Cryptosporidium* species. Parasitology 2016;2:e5.
127. Rajkhowa S, Rajkhowa C, Hazarika GC. Prevalence of *Cryptosporidium parvum* in mithuns (*Bos frontalis*) from India. Vet Parasitol. 2006;142:146-149.
128. Matos-Fernández MJ, Pereira-Bueno J, Ortega-Mora LM, Pilar-Izquierdo M, Ferre I, Rojo-Vázquez FA. Prevalencia de la infección por *Cryptosporidium parvum* en corderos, cabritos y terneros en la provincia de León. Acta Parasitol Port. 1993;1:211.
129. Munoz-fernandez M, Alvarez M, Lanza I, Carmenes P. Role of enteric pathogens in the aetiology of neonatal diarrhoea in lambs and goat kids in Spain. Epidemiol Inf. 1996;117:203-211.
130. Vieira LS, Silva MBO, Tolentono ACV, Lima JD, Silva AC. Outbreak of cryptosporidiosis in dairy goats in Brazil. Vet Rec. 1997;140:427-428.
131. Noordeen F, Rajapakse RPVJ, Faizal ACM, Horadagoda NU, Arulkanthan A. Prevalence of *Cryptosporidium* infection in goats in selected locations in three agroclimatic zones of Sri Lanka. Vet Parasitol. 2000;9:95-101.
132. Majewska AC, Werner A, Sulima P, Luty T. Prevalence of *Cryptosporidium* in sheep and goats bred on five farms in west central region of Poland. Vet Parasitol. 2000;89:269-275.
133. Castro-Hermida JA, Pors I, Poupin B, Ares-Mazás E, Chartier C. Prevalence of *Giardia duodenalis* and *Cryptosporidium parvum* in goat kids in western France. Small Rumin Res. 2005;56:259-264.
134. Bomfim TCB, Huber F, Gomes RS, Alves LL. Natural infection by *Giardia* sp. and *Cryptosporidium* sp. in dairy goats, associated with possible risk factors of the studied properties. Vet Parasitol. 2005;134:9-13.
135. Castro-Hermida JA, Delafosse A, Pors I, Ares-Mazás E, Chartier C*. Giardia duodenalis* and *Cryptosporidium parvum* infections in adult goats and their implications for neonatal kids. Vet Rec. 2005;157:623-627.
136. Park JH, Guk SM, Han ET, Shin EH, Kim JL, Chai JY. Genotype analysis of *Cryptosporidium* spp. prevalent in a rural village in Hwasun-gun, Republic of Korea. Korean J Parasitol. 2006;44:27-33.
137. Delafosse A, Castro-Hermida JA, Baudry C, Ares-Mazás E, Chartier C. Herd-level risk factors for *Cryptosporidium* infection in dairy goat kids in western France. Prev Vet Med. 2006;77:109-121.
138. Faleke OO, Sahabi K, Aliyu AB. Prevalence of *Cryptosporidium* in slaughter sheep and goats at Sokoto abattoir, Nigeria. Anim Prod Res Adv. 2006;2:179-182.
139. Misic Z, Katic-Radivojevic S, Kulisic Z. *Cryptosporidium* infection in lambs and goat kids in Serbia. Acta Vet. (Beograd) 2006;56:49-54.
140. Goma FY, Geurden T, Siwila J, Phiri IGK, Gabriel S, Claerebout E, et al. The prevalence and molecular characterisation of *Cryptosporidium* spp. in small ruminants in Zambia. Small Ruminant Res. 2007;72:77-80.
141. Castro-Hermida JA, Gonzalez-Warleta M, Mezo M. Natural infection by *Cryptosporidium parvum* and *Giardia* *duodenalis* in sheep and goats in Galacia (NW Spain). Small Rumin Res. 2007;72:96-100.
142. Geurden T, Thomas P, Casaert S, Vercruysse J, Claerebout E. Prevalence and molecular characterisation of *Cryptosporidium* and *Giardia* in lambs and goat kids in Belgium. Vet Parasitol. 2008;155:142-145.
143. Sanz Ceballos L, Illescas Gomez P, Sanz Sampelayo MR, Gil Extremera F, Rodriguez Osorio M. Prevalence of Cryptosporidium infection in goats maintained under semi-extensive feeding conditions in the southeast of Spain. Parasite 2009;16:315-318.
144. Bejan A, Mircean V, Radu C, Smaro S, Cozma V. Epidemiology of *Cryptosporidium* spp. infection in goat kids in the central and the northwest part of Romania. Rev Sci Parasitol. 2009;10:32-36.
145. Diaz P, Quilez J, Robinson G, Chalmers RM, Diez-Banos P, Morrondo P. Identification of *Cryptosporidium xiaoi* in diarrhoeic goat kids (Capra hircus) in Spain. Vet Parasitol. 2010;172:132-134.
146. Giadinis ND, Symeoudakis S, Papadopoulos E, Lafi SQ, Karatzias H. Comparison of two techniques for diagnosis of cryptosporidiosis in diarrhoeic goat kids and lambs in Cyprus. Trop Anim Health Prod. 2012;44:1561-1565.
147. Rieux A, Paraud C, Pors I, Chartier C. Molecular characterization of *Cryptosporidium* spp. in pre-weaned kids in a dairy goat farm in western France. Vet Parasitol. 2013;192:268-272.
148. Wang R, Li G, Cui B, Huang J, Cui Z, Zhang S, et al. Prevalence, molecular characterization and zoonotic potential of *Cryptosporidium* spp. in goats in Henan and Chongqing, China. Exp Parasitol. 2014;142:11-16.
149. Mi R, Wang X, Huang Y, Zhou P, Liu Y, Chen Y, et al. Prevalence and molecular characterization of *Cryptosporidium* in goats across four provincial level areas in China. PLoS ONE 2014;9:e111164
150. Koinari M, Lymbery AJ, Ryan UM. *Cryptosporidium* species in sheep and goats from Papua New Guinea. Exp Parasitol. 2014;141:134-137.
151. Paraud C, Pors I, Rieux A, Brunet S. High excretion of *Cryptosporidium ubiquitum* by peri-parturient goats in one flock in western France. Vet Parasitol. 2014;202:301-304.
152. Tzanidakis N, Sotiraki S, Claerebout E, Ehsan A, Voutzourakis N, Kostopoulou D, et al. Occurrence and molecular characterization of Giardia duodenalis and *Cryptosporidium* spp. in sheep and goats reared under dairy husbandry systems in Greece. Parasite 2014;21:45.
153. Díaz P, Quílez J, Prieto A, Navarro E, Pérez-Creo A, Fernández G, et al. *Cryptosporidium* species and subtype analysis in diarrhoeic pre-weaned lambs and goat kids from north-western Spain. Parasitol Res. 2015;114:4099-4105.
154. Parsons MB, Travis D, Lonsdorf EV, Lipende I, Roellig DM, Collins A, et al. Epidemiology and molecular characterization of *Cryptosporidium* spp. in humans, wild primates, and domesticated animals in the Greater Gombe Ecosystem, Tanzania. PLoS Negl Trop Dis. 2015;20:e0003529.
155. Sharma SP, Busang M. *Cryptosporidium* infection in sheep and goats in Southern Botswana and its public health significance. Glob J Anim Sci Res. 2015;3:329-36.
156. Akinkuotu OA, Fagbemi BO, Egbetade AO, Jacobs EB, Adeyanju J. Prevalence of *Cryptosporidium* coproantigens in goats in Ogun, southwest Nigeria. J Vet Adv. 2015;5:1122-1126.
157. Giadinis ND, Papadopoulos E, Lafi SQ, Papanikolopoulou V, Karanikola S, Diakou A, et al. Epidemiological observations on *Cryptosporidiosis* in diarrheic goat kids in Greece. Vet Med Int. 2015;764193.
158. Siddiki AZ, Mina SA, Farzana Z, Ayesa B, Das R, Hossain MA. Molecular characterization of *Cryptosporidium xiaoi* in goat kids in Bangladesh by nested PCR amplification of 18S rRNA gene. Asian Pac J Trop Biomed. 2015;5:202-207.
159. Romero-Salas D, Alvarado-Esquivel C, Cruz-Romero A, Aguilar Domínguez M, Ibarra-Priego N, Merino-Charrez OJ, et al. Prevalence of *Cryptosporidium* in small ruminants from Veracruz, Mexico. BMC Vet Res. 2016;12:1-6.
160. Marreros N, Frey CF, Willisch CS, Signer C, Ryser-Degiorgis M. Coprological analyses on apparently healthy Alpine ibex (*Capra ibex ibex*) from two Swiss colonies. Vet Parasitol. 2012;186:382-389.
161. Kotková M, Němejc K, Sak B, Hanzal V, Květoňová D, Hlásková L, et al. *Cryptosporidium ubiquitum*, *C. muris* and *Cryptosporidium* deer genotype in wild cervids and caprines in the Czech Republic. Folia Parasitol. 2016;63:003.
162. Davidson RK, Amundsen H, Lie ON, Luyckx K, Robertson LJ, Verocai, et al. Sentinels in a climatic outpost: Endoparasites in the introduced muskox (*Ovibos moschatus wardi*) population of Dovrefjell, Norway. Int J Parasitol Parasites Wildl. 2014;3:154-160.
163. Nouri M, Karami M. Asymptomatic cryptosporidiosis in nomadic shepherds and their sheep. J Infect. 1991;23:331-333.
164. Xiao L, Herd RP, Rings DM. Diagnosis of *Cryptosporidium* on a sheep farm with neonatal diarrhea by immunofluorescence assays. Vet Parasitol. 1993;47:17-23.
165. Nouri M, Mahdavi Rad S. Effect of nomadic shepherds and their sheep on the incidence of cryptosporidiosis in an adjacent town. J Infect. 1993;24:105-106.
166. Rezaeian M, Shahmoradi A, Dalimi A. *Cryptosporidium* in sheep as a source for human infection. Med J Islam Repub Iran 1993;6:273-274.
167. Causapé AC, Quílez J, Sánchez-Acedo C, del Cacho E, López-Bernad F. Prevalence and analysis of potential risk factors for *Cryptosporidium parvum* infection in lambs in Zaragoza (northeastern Spain). Vet Parasitol. 2002;104:287-298.
168. Pilarczyk B, Balicka-Ramisz A. Prevalences of *Cryptosporidium* sp. in farm animals in Western Pomerania. Electron J Pol Agric Univ, Anim Husb. 2002;5:08.
169. Ulutas B, Voyvoda H. Cryptosporidiosis in diarrhoeic lambs on a sheep farm. Turkiye Parazitologi Bergisi. 2004;28:15-17.
170. Alonso-Fresan MU, Garcıa-Alvarez A, Salazar-Garcıa F, Vazquez-Chagoyan JC, Pescador-Salas N, Saltijeral-Oaxaca J. Prevalence of *Cryptosporidium* spp. in asymptomatic sheep in family flocks from Mexico State. J Vet Med B 2005;52:482-483.
171. Ryan UM, Bath C, Robertson I, Read C, Elliot A, Mcinnes L, Traub R, Besier B. Sheep may not be an important zoonotic reservoir for *Cryptosporidium* and *Giardia* parasites. Appl Environ Microbiol. 2005;71:4992-4997.
172. Sevinc F, Uslu U, Derinbay O. The Prevalence of *Cryptosporidium* *parvum* in Lambs around Konya. Turk J Vet Anim Sci. 2005;29:1191-1194.
173. Alonso-Fresan MU, Saltijeral Oaxaca J, Velázquez Ordoñez V. *Cryptosporidium spp.* prevalence in lambs and ewes from the northern region in state of Mexico. Symposium of International Society for Animal Hygiene, Saint-Malo, France. 2004.
174. Santin M, Trout JM, Fayer R. Prevalence and molecular characterization of *Cryptosporidium* and *Giardia* species and genotypes in sheep in Maryland. Vet Parasitol. 2007;146:17-24.
175. Mueller-Doblies D, Giles M, Elwin K, Smith RP, Clifton-Hadley FA, Chalmers RM. Distribution of *Cryptosporidium* species in sheep in the UK. Vet Parasitol. 2008;154:214-219.
176. Quilez J, Torres E, Chalmers RM, Hadfield SJ, del Cacho E, Sanchez-Acedo C. *Cryptosporidium* genotypes and subtypes in lambs and goat kids in Spain. Appl Environ Microbiol. 2008;74:6026-6031.
177. Pritchard GC, Marshall JA, Giles M, Muller-Doblies D, Sayers AR, Marshall RN, et al. *Cryptosporidium* species in lambs submitted for diagnostic postmortem examination in England and Wales. Vet Rec. 2008;163:688-689.
178. Panousis N, Diakou A, Giadinis N, Papadopoulos E, Karatzias H, Haralampidis S. Prevalence of *Cryptosporidium* infection in sheep flocks with a history of lambs’ diarrhoea. Revue Med Vet. 2008;159:528-531.
179. Ozdal N, Tanritanir P, Goz Y, Deger S, Kozat S. Parasitic protozoans (*Eimeria*, *Giardia*, and *Cryptosporidium*) in lambs with diarrhoea in the Van Province (Turkey). Bulletin Vet Institute Pulawy 2009;53:47-51.
180. Yang R, Jacobson C, Gordon C, Ryan U. Prevalence and molecular characterisation of *Cryptosporidium* and *Giardia* species in pre-weaned sheep in Australia. Vet Parasitol. 2009;161:19-24.
181. Sari B, Arslan MO, Gicik Y, Kara M, Tasci GT. The prevalence of *Cryptosporidium* species in diarrhoeic lambs in Kars province and potential risk factors. Trop Anim Health Prod. 2009;41:819-826.
182. Wang Y, Feng Y, Cui B, Jian F, Ning C, Wang R, Zhang L, Xiao L. Cervine genotype is the major *Cryptosporidium* genotype in sheep in China. Parasitol Res. 2010;106:341-347.
183. Fiuza VR, Cosendey RI, Frazão-Teixeira E, Santín M, Fayer R, Oliveira FC. Molecular characterization of *Cryptosporidium* in Brazilian sheep. Vet Parasitol. 2011;175:360-362.
184. Sweeny JP, Ryan UM, Robertson ID, Yang R, Bell K, Jacobson C. Longitudinal investigation of protozoan parasites in meat lamb farms in southern Western Australia. Prev Vet Med. 2011;101:192-203.
185. Ahamed I, Yadav A, Katoch R, Godara R, Saleem T, Nisar NA. Prevalence and analysis of associated risk factors for *Cryptosporidium* infection in lambs in Jammu district. J Parasit Dis. 2013;39:414-417.
186. Imre K, Luca C, Costache M, Sala C, Morar A, Morariu S, et al. Zoonotic *Cryptosporidium* *parvum* in Romanian newborn lambs (*Ovis aries*). Vet Parasitol 2013;191:119-122.
187. Gharekhani J, Heidari H, Youssefi M. Prevalence of *Cryptosporidium* Infection in Sheep in Iran. Turkiye Parazitol Derg. 2014;38:22-25.
188. Yang R, Jacobson C, Gardner G, Carmichael I, Campbell AJD, Ng-Hublin J, et al. Longitudinal prevalence, oocyst shedding and molecular characterisation of *Cryptosporidium* species in sheep across four states in Australia. Vet Parasitol. 2014;200:50-58
189. Paz e Silva FM, Lopes RS, Saraiva Bresciani KD, Talamini Amarante AF, Araujo Jr JP. High occurrence of *Cryptosporidium ubiquitum* and *Giardia duodenalis* genotype E in sheep from Brazil. Acta Parasitol. 2014;59:193-196.
190. Zucatto AS, Aquino MCC, Inácio SV, Figueiredo RN, Pierucci JC, Perri SHV, et al. Molecular characterisation of *Cryptosporidium* spp. in lambs in the South Central region of the State of São Paulo. Arq Bras Med Vet Zootec. 2015;67:441-446.
191. Yang R, Gardner GE, Ryan U, Jacobson C. Prevalence and pathogen load of *Cryptosporidium* and *Giardia* in sheep faeces collected from saleyards and in abattoir effluent in Western Australia. Small Ruminant Res. 2015;130:216-2220.
192. Li P, Cai J, Cai M, Wu W, Li C, Lei M, et al. Distribution of *Cryptosporidium* species in tibetan sheep and yaks in Qinghai, China. Vet Parasitol. 2016;215:58-62.
193. Zhou C, He G, Zhang L. Investigation on *Cryptosporidium* infection in yaks. Chin J Zoonoses 2009;25:389-390.
194. Ma L, Lu Y, Cai Q, Wang G, Niu X, Ye X, et al. Serological investigation of yak cryptosporidiosis in Qinghai Province. J Domest Anim Ecol. 2011;32:47-49.
195. Mi R, Wang X, Li C, Huang Y, Zhou P, Li Z, et al. Prevalence and genetic characterization of *Cryptosporidium* in yaks in Qinghai Province of China. PLoS One 2013;8:e74985.
196. Ma J, Cai J, Ma J, Feng Y, Xiao L. Occurrence and molecular characterization of *Cryptosporidium* spp. in yaks (*Bos grunniens*) in China. Vet Parasitol. 2014;202:113-118.
197. Qin SY, Zhang XX, Zhao GH, Zhou DH, Yin MY, et al. First report of *Cryptosporidium* spp. in white yaks in China. Parasit Vectors 2014;7:230.
198. Qi M, Cai JZ, Wang RJ, Li JQ, Jian FC, Huang JY, et al. Molecular characterization of *Cryptosporidium* spp. and *Giardia duodenalis* from yaks in the central western region of China. BMC Microbiol. 2015;15:108.
199. Li P, Cai J, Cai M, Wu W, Li C, Lei M, et al. Distribution of *Cryptosporidium* species in Tibetan sheep and yaks in Qinghai, China. Vet Parasitol. 2016;215:58-62.
200. Starkey SR, Johnson AL, Ziegler PE, Mohammed HO. An outbreak of cryptosporidiosis among alpaca crias and their human caregivers. J Am Vet Med Assoc. 2007;231:1562-1567.
201. Waitt LH, Cebra CK, Firshman AM, McKenzie EC, Schlipf Jr JW. Cryptosporidiosis in 20 alpaca crias. J Am Vet Med Assoc. 2008;233:294-8.
202. Twomey DF, Barlow AM, Bell S, Chalmers RM, Elwin K, Giles M, et al. Cryptosporidiosis in two alpaca (*Lama pacos*) holdings in the South‐West of England. Vet J. 2008;175:419-422.
203. López-Urbina MT, González AE, Gomez-Puerta LA, Romero-Arbizu MA, Rojo-Vázquez FA, Xiao L, et al. Prevalence of Neonatal Cryptosporidiosis in Andean Alpacas (*Vicugna pacos*) in Perú. Parasitol Jorl. 2009;3:9-13.
204. Burton AJ, Nydam DV, Mitchell KJ, Bowman DD. Fecal shedding of *Cryptosporidium* oocysts in healthy alpaca crias and their dams. J Am Vet Med Assoc. 2012;241:496-498.
205. Gomez-Couso H, Ortega-Mora LM, Aguado-Martinez A, Rosadio-Alcantara R, Maturrano-Hernandez L, Luna-Espinoza L, et al. Presence and molecular characterisation of *Giardia* and *Cryptosporidium* in alpacas (*Vicugna pacos*) from Peru. Vet Parasitol. 2012;187:414-420.
206. Wessels J, Wessels M, Featherstone C, Pike R. Cryptosporidiosis in eight-month-old weaned alpacas. Vet Rec. 2013;173:426-427.
207. Rojas M, Manchego A, Rocha CB, Fornells LA, Silva RC, Mendes GS, et al. Outbreak of diarrhea among preweaning alpacas (*Vicugna pacos*) in the southern Peruvian highland. J Infect Dev Ctries. 2016;10:269-274.
208. Fayer R, Phillips L, Anderson BC, Bush M. Chronic cryptosporidiosis in a bacterial camel (*Camelus bactrianus*). J Zoo Wildl Med. 1991;22:228-232.
209. Borji H, Razmi GH, Movassaghi AR, Naghibi AG, Maleki M. Prevalence of *Cryptosporidium* and *Eimeria* infections in dromedary (*Camelus dromedarius*) in abattoir of Mashhad, Iran. J Camel Pract Res. 2009;16:167-170.
210. Razavi SM, Oryan A, Bahrami S, Mohammadalipour A, Gowhari M. Prevalence of *Cryptosporidium* infection in camels (*Camelus dromedarius*) in a slaughterhouse in Iran. Trop Biomed. 2009;26:267-273.
211. Nazifi S, Behzadi MA, Haddadi SH, Raayat Jahromi A, Mehrshad S, Amadon A. Prevalence of *Cryptosporidium* isolated from dromedary camels (*Camelus dromedarius*) in Qeshm Island, Southern Iran. Comp Clin Path. 2010;19:311-314.
212. Yakhchali M, Moradi T. Prevalence of *Cryptosporidium*-like infection in onehomped camels (*Camelus dromedarius*) of Northwestern Iran. Parasite 2012;19:71-75.
213. Sazmand A, Rasooli A, Nouri M, Hamidinejat H, Hekmatimoghaddam S. Prevalence of *Cryptosporidium* spp. in camels and involved people in Yazd Province, Iran. Iran J Parasitol. 2012;7:80-84.
214. Liu X, Zhou X, Zhong Zh, Deng J, Chen W, Cao S, et al. Multilocus genotype and subtype analysis of *Cryptosporidium andersoni* derived from a Bactrian camel (*Camelus bactrianus*) in China. Parasitol Res. 2014;113:2129-2136.
215. Rulofson FC, Atwill ER, Holmberg CA. Fecal shedding of *Giardia duodenalis*, *Cryptosporidium parvum*, *Salmonella* organisms, and *Escherichia coli* O157:H7 from llamas in California. Am J Vet Res. 2001;62:637-642.
216. Cebra CK, Mattson DE, Baker RJ, Sonn RJ, Dearing PL. Potential pathogens in feces from unweaned llamas and alpacas with diarrhea. J Am Vet Med Assoc. 2003;223:1806-1808.
217. Deng MQ, Cliver DO. Improved immunofluorescence assay for detection of *Giardia* and *Cryptosporidium* from asymptomatic adult cervine animals. Parasitol Res. 1999;85:733-736.
218. Siefker C, Rickard LG, Pharr GT, Simmons JS, O'Hara TM. Molecular characterization of *Cryptosporidium* sp. isolated from northern Alaskan caribou (*Rangifer tarandus*). J Parasitol. 2002;88:213-216.
219. Kemper N, Aschfalk A, Höller C. *Campylobacter* spp., *Enterococcus* spp., *Escherichia coli*, *Salmonella* spp., *Yersinia* spp., and *Cryptosporidium* oocysts in semi-domesticated reindeer (*Rangifer tarandus tarandus*) in Northern Finland and Norway. Acta Vet Scand. 2006;48:7.
220. Hamnes, I.S., Gjerde, B.K., Robertson, L., Vikøren, T., Handeland, K., 2006. Prevalence of *Cryptosporidium* and *Giardia* in free-ranging wild cervids in Norway. Vet Parasitol. 141, 30-41.
221. Johnson D, Jane Harms N, Larter NC, Elkin BT, Tabel H, Wei G. Serum biochemistry, serology, and parasitology of boreal caribou (*Rangifer tarandus caribou*) in the Northwest Territories, Canada. J Wildl Dis. 2010;46:1096-1107.
222. Majewska AC, Kasprzak W, Werner A. Prevalence of *Cryptosporidium* in mammals housed in Poznan zoological garden, Poland. Acta Parasitol. 1997;42:195-198.
223. Sturdee AP, Chalmers RM, Bull SA. Detection of *Cryptosporidium* oocysts in wild mammals of mainland Britain. Vet Parasitol. 1999;80:273-280.
224. Lourenço AM, Bruno de Sousa C, Matos O, Alves M, Pereira da Fonseca I, Antunes F. Estudo preliminar da criptosporidiose em gamos (*Dama dama L.*) da Tapada Nacional de Mafra (Portugal). Acta Parasitol Port 2000;7:29-31.
225. Wang R, Wang J, Sun M, Dang H, Feng Y, Changshen Ning C, et al. Molecular characterization of the *Cryptosporidium* cervine genotype from a sika deer (*Cervus nippon Temminck*) in Zhengzhou, China and literature review. Parasitol Res. 2008;103:865-869
226. Wells B, Shaw H, Hotchkiss E, Gilray J, Ayton R, Green J, et al. Prevalence, species identification and genotyping *Cryptosporidium* from livestock and deer in a catchment in the Cairngorms with a history of a contaminated public water supply. Parasites Vector. 2015;8:66.
227. Castro-Hermida JA, Garcia-Presedo I, Gonzalez-Warleta M, Mezo M. Prevalence of *Cryptosporidium* and *Giardia* in roe deer (*Capreolus capreolus*) and wild boars (*Sus scrofa*) in Galicia (NW, Spain). Vet Parasitol. 2011;179:216-219.
228. García-Presedo I, Pedraza-Díaz S, González-Warleta M, Mezo M, Gómez-Bautista M, Ortega-Mora LM, et al. The first report of *Cryptosporidium bovis*, *C. ryanae* and *Giardia duodenalis* sub-assemblage A-II in roe deer (*Capreolus capreolus*) in Spain. Vet Parasitol. 2013;197:658-664.
229. Fayer R, Fischer JR, Sewell CT, Kavanaugh DM, Osborn DA. Spontaneous cryptosporidiosis in captive white-tailed deer (*Odocoileus virginianus*). J Wildl Dis. 1996;32:619-622.
230. Rickard LG, Siefker C, Boyle CR, Gentz EJ. The prevalence of *Cryptosporidium* and *Giardia* spp. in fecal samples from free-ranging white-tailed deer (*Odocoileus virginianus*) in the southeastern United States. J Vet Diagn Invest. 1999;11:65-72.
231. Perz JF, Blancq SML. *Cryptosporidium parvum* Infection Involving Novel Genotypes in Wildlife from Lower New York State. Appl Environ Microbiol. 2001;67:1154-1162.
232. Ziegler PE, Wade SE, Schaaf SL, Stern DA, Nadareski CA, Mohammed HO. Prevalence of *Cryptosporidium* species in wildlife populations within a watershed landscape in southeastern New York State. Vet Parasitol. 2007;147:176-184.
233. Feng Y, Alderisio KA, Yang W, Blancero LA, Kuhne WG, Nadareski CA, et al. *Cryptosporidium* Genotypes in Wildlife from a New York Watershed. Appl Environ Microbiol. 2007;73:6475-6483
234. Santin M, Fayer R. *Enterocytozoon bieneusi*, *Giardia*, and *Cryptosporidium* infecting White-tailed Deer. J Eukaryot Microbiol. 2015;62:34-43.

# **Atwill ER, Sweitzer RA, Pereira MG, Gardner IA, Van VD, Boyce WM. Prevalence of and associated risk factors for shedding *Cryptosporidium parvum* oocysts and *Giardia* cysts within feral pig populations in California. Appl Environ Microbiol. 1997;63:3946-3949.**

1. Hampton J, Spencer PBS, Elliot AD, Thompson RCA. Prevalence of zoonotic pathogens from feral pigs in major public drinking water catchments in Western Australia. EcoHealth 2006;3:103-108.
2. [Quílez J](https://www.ncbi.nlm.nih.gov/pubmed/?term=Quílez J%5BAuthor%5D&cauthor=true&cauthor_uid=9011017), [Sánchez-Acedo C](https://www.ncbi.nlm.nih.gov/pubmed/?term=Sánchez-Acedo C%5BAuthor%5D&cauthor=true&cauthor_uid=9011017), [Clavel A](https://www.ncbi.nlm.nih.gov/pubmed/?term=Clavel A%5BAuthor%5D&cauthor=true&cauthor_uid=9011017), [del Cacho E](https://www.ncbi.nlm.nih.gov/pubmed/?term=del Cacho E%5BAuthor%5D&cauthor=true&cauthor_uid=9011017), [López-Bernad F](https://www.ncbi.nlm.nih.gov/pubmed/?term=López-Bernad F%5BAuthor%5D&cauthor=true&cauthor_uid=9011017). Prevalence of *Cryptosporidium* infections in pigs in Aragón (northeastern Spain). [Vet Parasitol.](https://www.ncbi.nlm.nih.gov/pubmed/9011017) 1996;67:83-8.
3. Guselle N, Olson ME. Human pathogens in Alberta Hog Operations. Report to Alberta Hog Industry Development Fund, Alberta Pork Producers. 1999.
4. Izumiyama S, Furukawa I, Kuroki T, Yamai S, Sugiyama H, Yagita K, et al. Prevalence of *Cryptosporidium parvum* infections in weaned piglets and fattening porkers in Kanagawa Prefecture, Japan. Rev Soc Bras Med Trop. 2001;54:23-26.
5. Wieler LH, Iliefe A, Herbst W, Bauer C, Vieler E, Bauerfeind R, et al. Prevalence of enteropathogens in suckling and weaned piglets with diarrhoea in Southern Germany. J Vet Med B 2001;48:151-159.
6. Ryan UM, Samarasinghe B, Read C, Buddle JR, Robertson ID, Thompson RCA. Identification of a novel *Cryptosporidium* genotype in pigs. Appl Environ Microbiol. 2003;69:3970-3974.
7. Misic ZB, Katic-Radivojevic S, Kulisic Z. *Cryptosporidium* infection in nursing, weaning and post-weaned piglets and sows in the Belgrade district. Acta Vet Beograd. 2003;53:361-366.
8. Yu JR, Seo M. Infection status of pigs with *Cryptosporidium parvum*. Korean J Parasitol. 2004;42:45-47.
9. Vitovec J, Hamadejova K, Landova L, Kvac M, Kvetonova D, Sak B. Prevalence and pathogenicity of *Cryptosporidium* *suis* in pre- and post-weaned pigs. J Vet Med B. 2006;53:239-243.
10. Zintl A, Neville D, Maguire D, Fanning S, Mulcahy G, Smith HV, et al. Prevalence of *Cryptosporidium* species in intensively farmed pigs in Ireland. Parasitology 2007;134:1575-1582.
11. Yatswako S, Faleke OO, Gulumbe ML, Daneji AI. *Cryptosporidium* oocysts and *Balantidium coli* cysts in Pigs reared semi-intensively in Zuru, Nigeria. Pakistan J Bio Sci. 2007;10:3435-3439.
12. Johnson J, Buddle R, Reid S, Armson A, Ryan U. Prevalence of *Cryptosporidium* genotypes in pre and post-weaned pigs in Australia. Exp Parasitol. 2008;119:418-421.
13. Kváč M, Sak B, Hanzlíková D, Kotilová J, Květoňová D. Molecular characterization of *Cryptosporidium* isolates from pigs at slaughterhouses in South Bohemia, Czech Republic. Parasitol Res. 2009;104:425-428.
14. Maikai BV, Umoh JU, Kwaga JKP, Maikai VA, Egege SC. Prevalence and risk factors associated with faecal shedding of *Cryptosporidium* oocysts in piglets, Kaduna, Nigeria. 2009;1:001-004.
15. Kváč M, Hanzlíková D, Sak B, Květoňová D. Prevalence and age-related infection of *Cryptosporidium suis*, *C. muris* and *Cryptosporidium pig* genotype II in pigs on a farm complex in the Czech Republic. Vet Parasitol. 2009;160:319-322.
16. Wang R, Qiu S, Jian F, Zhang S, Shen Y, Zhang L, et al. Prevalence and molecular identification of *Cryptosporidium* spp. in pigs in Henan, China. Parasitol Res. 2010;107:1489-1494
17. Featherstone CA, Marshall JA, Giles M, Sayers AR, Pritchard GC. *Cryptosporidium* species infection in pigs in East Anglia. Vet Rec. 2010;166:51-52.
18. Yin J, Shen Y, Yuan Z, Lu W, Xu Y, Cao J. Prevalence of the *Cryptosporidium* Pig Genotype II in Pigs from the Yangtze River Delta, China. PLoS ONE. 2011;6:e20738.
19. Fiuza VRS, Gallo SSM, Frazao-Teixeira E, Santı´n M, Fayer R., Oliveira FCR. *Cryptosporidium* pig genotype II diagnosed in Pigs from the State of Rio De Janeiro, Brazil J Parasitol. 2011;97:146-147
20. Siwila J, Mwape KE. Prevalence of *Cryptosporidium* spp. and *Giardia duodenalis* in pigs in Lusaka, Zambia. Onderstepoort J Vet Res. 2012;79:E1-5
21. Němejc K, Sak B, Květoňová D, Kernerová N, Rost M, Cama VA, Kváč M. Occurrence of *Cryptosporidium suis* and *Cryptosporidium scrofarum* on commercial swine farms in the Czech Republic and its associations with age and husbandry practices. Parasitol Res. 2013;112:1143-1154.
22. Zhang W, Yang F, Liu A, Wang R, Zhang L, Shen Y, Cao J, Ling H. Prevalence and genetic characterizations of *Cryptosporidium* spp. in pre-weaned and post weaned piglets in Heilongjiang Province, China. PLoS ONE. 2013;8:e67564.
23. Rzeżutka A, Kaupke A, Kozyra I, Pejsak Z. Molecular studies on pig cryptosporidiosis in Poland. Pol J Vet Sci**.** 2014;17:577-582.
24. Yui T, Nakajima T, Yamamoto N, Kon M, Abe N, Matsubayashi M, et al. Age-related detection and molecular characterization of *Cryptosporidium suis* and *Cryptosporidium scrofarum* in pre- and post-weaned piglets and adult pigs in Japan. Parasitol Res. 2014;113:359-365.
25. Yui T, Shaibahara T, Kon M, Yamamoto N, Kameda M, Taniyama H. Epidemiological studies on intestinal protozoa in pigs in Saitama, Japan. JPN AGR RES Q. 2014;48:87-93.
26. Petersen HH, Jianmin W, Katakam KK, Mejer H, Thamsborg SM, Anders Dalsgaard A, et al. *Cryptosporidium* and *Giardia* in Danish organic pig farms: seasonal and age-related variation in prevalence, infection intensity and species/genotypes. Vet Parasitol. 2015;214:29-39.
27. Lin Q, Wang XY, Chen JW, Ding L, Zhao GH. *Cryptosporidium suis* infection in post-weaned and adult pigs in Shaanxi Province, Northwestern China. Korean J Parasitol. 2015;53:113-117.
28. Němejc K, Sak B, Květoňová D, Hanzel V, Jenikova M. The first report on *Cryptosporidium suis* and *Cryptosporidium* pig genotype II in Eurasian wild boars (*Sus scrofa*) (Czech Republic). Vet Parasitol. 2012;184:122-125.
29. Laatamna AK, Wagnerová P, Sak B, Květoňová D, Xiao L, Rost M. et al. Microsporidia and *Cryptosporidium* in horses and donkeys in Algeria: Detection of a novel *Cryptosporidium hominis* subtype family (Ik) in a horse. Vet Parasitol. 2015;208:135-142.
30. Xiao L, Herd RP. Epidemiology of equine *Cryptosporidium* and *Giardia* infections. Equine Vet J. 1994;26:14-17.
31. Johnson E, Atwill ER, Filkins ME, Kalush J. The prevalence of shedding of *Cryptosporidium* and *Giardia* spp. based on a single fecal sample collection from each of 91 horses used for backcountry recreation. J Vet Diagn Invest. 1997;9:56-60.
32. Forde KN, Swinker AM, Traub-Dargatz JL, Cheney JM. The prevalence of *Cryptosporidium/Giardia* in the trail horse population utilizing public lands in Colorado. J Equine Vet Sci. 1998;18:38-40.
33. Bray RE, Wickler SJ, Cogger EA, Atwill ER, London C, Gallinol JL, et al. Endoparasite infection and *Cryptosporidium/Giardia* in feral horses on public land. J Equine Vet Sci. 1998;18:41-43.
34. Majewska AC, Werner A, Sulima P, Luty T. Survey on equine cryptosporidiosis in Poland and the possibility of zoonotic transmission. Ann Agric Environ Med. 1999;6:161-165.
35. Grinberg A, Learmonth JJ, Oliver L, Roe W, Leyland M, Pomroy W. Identification of *Cryptosporidium parvum* “cattle” genotype from a severe outbreak of neonatal foal diarrhoea. Vet Rec. 2003;153:628-630.
36. Majewska AC, Solarczyk P, Tamang L, Graczyk TK. Equine *Cryptosporidium parvum* infections in western Poland. Parasitol Res. 2004;93:274-278.
37. Chalmers AM, Thomas AL, Butler BA, Davies Morel MCG. Identification of *Cryptosporidium parvum* genotype 2 in domestic horses. Vet Rec. 2005;159:49-50.
38. Tavassoli M, Sodagar-Skandarabadi M, Soltanalinejad F. A survey on cryptosporidial infection in horse in Urmia area, northwestern Iran. Iran J Vet Res. 2007;8:86-90.
39. Gomes AD, Barretta C, Ziegler DPZ, Sausen L, Stoever N, Sangioni LA, et al. Prevalence of *Cryptosporidium* spp*.* and *Giardia* sp*.* infection in horses stabled in the Jockey Club of Santa Maria, Brazil. Ciênc Rural. 2008;38:2662-2665.
40. Grinberg A, Pomroy WE, Carslake HB, Shi Y, Gibson IR, Drayton BM. A study of neonatal cryptosporidosis of foals in New Zealand. New Zealand Vet J. 2009;57:284-289.
41. De Souza PNB, Bomfim, TCB, Huber F, Abboud LCS, Gomes RS. Natural infection by *Cryptosporidium* sp., *Giardia* sp. and *Eimeria leuckarti* in three groups of equines with different handlings in Rio de Janeiro, Brazil. Vet Parasitol. 2009;160:327-333.
42. Papazahariadou M, Papadopoulos E, Diakou A, Ptochos S. Gastrointestinal parasites of stabled and grazing horses in central and northern Greece. J Equine Vet Sci. 2009;29:233-236.
43. Mirian SJ, Asadi MR, Ferdowsi HR, Rezakhani AH. A survey on horse cryptosporidial infection in Tehran province. Arch Razi Inst. 2010;65:45-47.
44. Veronesi F, Passamonti F, Caccio S, Diaferia M, Piergili Fioretti D. Epidemiological survey on equine *Cryptosporidium* and *Giardia* infections in Italy and molecular characterization of isolates. Zoonoses Public Health. 2010;57:510-517
45. Marques SMT. Cryptosporidiosis in horses of urban areas of Porto Alegre, Rio Grande do Sul, southern Brazil. J Equine Vet Sci. 2010;30:356-358
46. Burton AJ, Nydam DV, Dearen TK, Mitchell K, Bowman DD, Xiao L. The prevalence of *Cryptosporidium*, and identification of the *Cryptosporidium* horse genotype in foals in New York State. Vet Parasitol. 2010;174:139-144.
47. Butty ET. Detection of *Cryptosporidium* and *Giardia doudenalis* in equines in Nineveh, Iraq. Iraq J Vet Sci. 2011;25:43-46.
48. Inácio SV, de Brito RLL, Zucatto AS, Coelho WM.D, de Aquino MCC, Aguirre AAR, et al. *Cryptosporidium* spp. infection in mares and foals of the northwest region of São Paulo State, Brazil. Rev Bras Parasitol Vet. 2012;21:355-358.
49. Laatamna AK, Wagnerová P, Sak B, Květoňová D, Aissi M, Rost M, et al. Equine cryptosporidial infection associated with *Cryptosporidium* hedgehog genotype in Algeria. Vet Parasitol. 2013;197:350-353
50. Guo PF, Chen TT, Tsaihong JC, Ho GD, Cheng PC, Tseng YC, et al. Prevalence and species identification of *Cryptosporidium* from fecal samples of horses in Taiwan. Southeast Asian J Trop Med Public Health. 2014;45:6-12.
51. Wagnerová P, Sak B, McEvoy J, Rost M, Matysiak AP, Ježková J, et al. Genetic diversity of *Cryptosporidium* spp. including novel identification of the *Cryptosporidium* *muris* and *Cryptosporidium tyzzeri* in horses in the Czech Republic and Poland. Parasitol Res. 2015;114:1619-24.
52. Galuppi R, Piva S, Castagnetti C, Iacono E, Tanel S, Pallaver F, et al. Epidemiological survey on *Cryptosporidium* in an Equine Perinatology Unit. Vet Parasitol. 2015;210:10-18.
53. Qi M, Zhou H, Wang H, Wang R, Xiao L, Arrowood MJ, Li J, Zhang L. Molecular identification of *Cryptosporidium* spp. and *Giardia duodenalis* in grazing horses from Xinjiang, China. Vet Parasitol. 2015;209:169-72.
54. Liu A, Zhang J, Zhao J, Zhao W, Wang R, Zhang L. The first report of *Cryptosporidium andersoni* in horses with diarrhea and multilocus subtype analysis. Parasit Vectors 2015;8:483.
55. Wagnerová P, Sak B, McEvoy J, Rost M, Sherwood D, Holcomb K, Kvac M. *Cryptosporidium parvum* and *Enterocytozoon bieneusi* in American Mustangs and Chincoteague ponies. Exp Parasitol. 2016;162:24-27
56. McKenzie DM, Diffay BC. Diarrhoea associated with cryptosporidial oocyst shedding in a Quarterhorse stallion. Aust Vet J. 2000;78:27-28.
57. Kodádková A, Kváč M, Ditrich O, Sak B, Xiao L. *Cryptosporidium muris* in a Reticulated Giraffe (*Giraffa camelopardalis reticulata*). J Parasitol. 2010;96:211-212.
